# Visualizing Reversible Uranyl (UO_2_^2+^) Capture and Release With Self‐Indicating Porous Organic Polymers

**DOI:** 10.1002/smsc.70416

**Published:** 2026-10-05

**Authors:** Nilojyoti Sahoo, Laraib Akhtar, Rameshwar Rai, Srinivasu Kancharlapalli, Venkata Suresh Mothika

**Affiliations:** ^1^ Department of Chemistry Indian Institute of Technology (IIT) Kanpur Kanpur India; ^2^ Chemistry Division Bhabha Atomic Research Centre Mumbai India; ^3^ Homi Bhabha National Institute Mumbai India

**Keywords:** POPs, rapid kinetics, recyclability, self‐indicating, uranium capture

## Abstract

Seawater uranium extraction is a promising sustainable energy pathway; however, practical application requires advanced adsorbents that combine reversible, high uranium‐uptake capacity with the ability to track sorption cycles. Herein, we report self‐indicating porous organic polymers (POPs), *Sal‐POP* and *Im‐POP*, containing uranyl (UO_2_^2+^) ion‐coordinating salphen and *o*‐hydroxy imine cores with UO_2_^2+^ binding energies up to −71.94 kcal mol^−1^ as uranium adsorbents, and demonstrate their reversible, high uranium extraction capacities up to 0.114 g g^−1^ h^−1^. Enabled by the extended conjugation of salphen unit and strong UO_2_^2+^ binding, *Sal*‐*POP* readily changes color at uranium saturation from yellow to black and allows visual tracking of uranium adsorption/desorption cycles. With a high density of UO_2_^2+^ binding sites, both *Sal*‐*POP* and *Im*‐*POP* demonstrate rapid, reversible, and high uranium uptake capacity, with distribution coefficients (*K*_d_) of 1.88 × 10^5^ and 2.94 × 10^5^ mL g^−1^, and removal efficiencies up to 70% and 78%**.** The uranyl capture is highly reversible, and the POP color changes from black to yellow upon simple elution with Na_2_CO_3_, thus acting as a self‐indicating POP adsorbent.

## Graphical abstract

Uranium capture from seawater is a promising sustainable strategy, and the adsorbents showing high uptake capacity and sorption trackability are essential. The work describes self‐indicating porous organic polymers that exhibit visible color changes during uranium adsorption and desorption cycles allow tracking of uranium‐free, saturated states without the need for sophisticated tools, and are suitable for real‐world practical deployment.

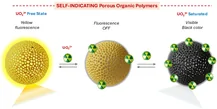

## 1 Introduction

Nuclear energy is widely regarded as a promising low‐carbon power source, and the vast uranium reserves (ca. 4.5 billion tons) in seawater make it a truly sustainable alternative for future energy demands [1, 2, 3, 4, 5]. In contrast to current industrial uranium ore mining and fuel processing methods, which inevitably risk releasing uranium into soil and aquatic environments, seawater extraction is a promising alternative [6, 7]. In seawater, uranium exists as a uranyl (UO_2_^2+^) carbonate at ultradilute concentrations (ca. 3–4 ppb) [8]; thus, the seawater extraction requires adsorbents that possess high binding energy and are selective to UO_2_^2+^ [9, 10, 11], particularly in the presence of common competing ions such as vanadium (V^5+^) [12, 13, 14, 15, 16]. Moreover, adsorbent materials that exhibit reversible visual color changes during uranium capture and release cycles are advantageous as they allow real‐time tracking of the uranium sorption process, circumvent the need for sophisticated tools, and are suitable for practical applications.

Porous organic polymers (POPs) [17, 18, 19] have recently emerged as a highly promising platform for both uranium detection and capture [20, 21]. Unlike metal–organic frameworks [22, 23, 24], POPs offer undoubtedly high chemical stability through robust C─C/C─N linkages and a rigid aromatic skeleton, enabling reliable performance under harsh conditions (acidic, basic, saline) [25, 26, 27]. POPs possess high accessible surface areas and a rigid, three‐dimensional porous structure that facilitates rapid uranyl transport and superior capture efficiencies [28, 29, 30]. Furthermore, their structural tunability allows integration of chromophoric units for intense color changes, while the extended π‐conjugation renders an amplified transduction signal response, even for ultratrace chemical perturbations [31, 32, 33, 34, 35]. Relying on high selectivity of uranyl ions to amidoxime (AO) in the presence of a complex matrix of interfering ions of seawater, several AO‐POPs have been reported for uranium capture from seawater; however, high‐temperature synthesis and laborious postsynthetic modification raise the cost of the process, and also the pore blockage by large AO groups restricts the access of uranyl cations to the bulk of the POPs and limit uptake [36, 37, 38, 39]. In this context, POPs containing intrinsic uranyl‐binding motifs avoid pore occlusion or extra steps, and postsynthetic modifications are significant for efficient UO_2_^2+^ capture but require a rational design strategy [40, 41, 42].

Here, we report self‐indicating POP uranium adsorbents, *Sal‐POP* and *Im‐POP*, containing salphen and *o*‐hydroxy imine uranyl binding units for rapid, reversible uranyl (UO_2_^2+^) extraction and exhibit visible color changes during the uranium capture‐release cycles essential for practical deployment (Scheme 1). Both POPs exhibit strong coordination interactions with uranyl cations and showed exceptionally high uranium uptake capacities of 0.114 g g^−1^ within 1 h under a 10 ppm UO_2_^2+^ concentration of simulated seawater. The UO_2_^2+^ uptake capacities have significantly increased with increasing UO_2_^2+^ concentration, with the highest uptake of ca. 1.05 g g^−1^ d^−1^ UO_2_^2+^ at 100 ppm in 24 h of POPs. These UO_2_^2+^ uptake values are higher than those reported to date (Scheme 1). Such high uptakes were also corroborated with the high *K*_d_ and removal efficiencies of 1.88 × 10^5^, 2.94 × 10^5^ mL g^−1^ and 70% and 78% for *Sal‐POP* and *Im‐POP*, respectively. Of the POPs, *Sal‐POP* also displays a striking reversible colorimetric change (yellow ⇌ black) during uranium adsorption–desorption cycles due to uranyl binding and subsequent changes in the optical absorption of the POP. Such complexation was confirmed by the Stern–Volmer quenching analysis at a lower concentration of UO_2_^2+^. Both POPs are easily regenerated after uranium saturation by simple elution with Na_2_CO_3_ and retain high uptake capacity over multiple cycles.

**SCHEME 1:**
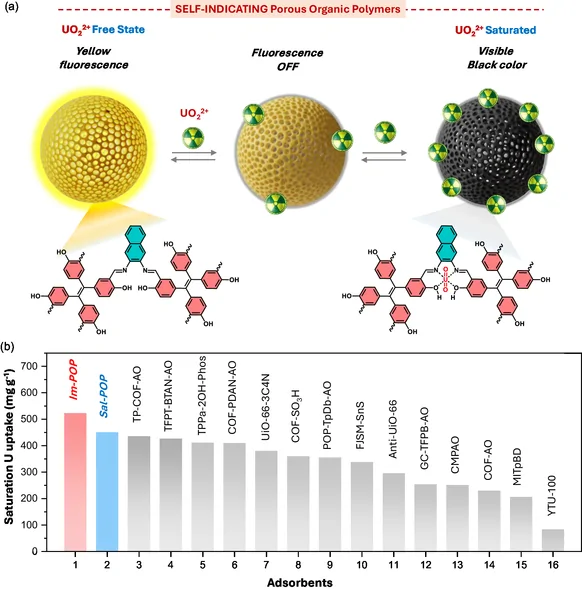
Top: illustration of self‐indicating uranium adsorbing POP that allows tracking of the initiation of uranium adsorption (by fluorescence turn off), saturation (by colorimetric changes), and the desorption process (both colorimetric and fluorescence turn on). Bottom: comparison of uranyl uptake properties of *Sal‐POP* and *Im‐POP* with previously reported POPs.

## 2 Results and Discussion

### 2.1 Structural Characterization

Both POPs, *Sal‐POP* and *Im‐POP*, were synthesized via a Schiff‐base condensation, yielding high‐density salphen and *o*‐hydroxy imine coordinating sites within a three‐dimensional porous network for UO_2_^2+^ capture. *Sal‐POP* and *Im‐POP* were synthesized by the condensation reaction of *M2* with naphthalene‐2,3‐diamine (*M1*) or *p*‐phenylenediamine (*M3*), respectively, in a solvent mixture of *n*‐butanol/*o*‐dichlorobenzene under solvothermal conditions (Figure 1 and Scheme S1) (see Supporting Information for details). The successful formation of imine‐linked porous networks was initially supported by Fourier transform infrared (FT‐IR) spectroscopy. *Sal‐POP* showed C═N stretching vibration (*ν*_C═N_) at 1610 cm^−1^ and a broad band at 3046 and 3592 cm^−1^ corresponding to stretching vibration of OH (*ν*_O─H_) [43]. Similarly, *Im‐POP* showed a C═N stretching vibration (ν_C═N_) at 1616 cm^−1^, indicating the formation of the POPs via the imine linkages (Figures 2a, S1, and S2) [44]. Moreover, both POPs showed no carbonyl or amine stretching vibrations related to monomers, indicating the absence of any monomer impurities in the polymers. Formation of the POPs was further strengthened by solid‐state ^13^C CP/MAS NMR, as shown in Figure 2b. *Sal‐POP* and *Im‐POP* displayed signals at 160 and 159 ppm, corresponding to imine (C═N) carbon, confirming the formation of the imine linkage. Similarly, both POPs showed signals at 152–156 and 144–148 ppm that correspond to phenolic carbons. The other peaks in the 105–136 ppm range are ascribed to aromatic carbons of POPs [13, 31, 45]. X‐ray photoelectron spectroscopy (XPS) offered additional insight into the bonding environments of the POPs. For *Sal‐POP*, the C 1s spectrum was fitted into multiple peaks with binding energies of 284.30, 285.24, and 286.49 eV, corresponding to the C═C, C═N, and phenolic C─O bonding environment (Figure 2c). The relative percentage of C, N, and O atom are 76.36%, 6.07%, and 17.57%, respectively, indicating a high density of accessible O/N‐containing binding sites in the POPs (Table S1), while the N 1s and O 1s deconvoluted spectra showed peaks at 398.54, 400.34 eV and 532.26, 533.31 eV corresponding to C═N, C─N and ─OH, C─O bonding environment related to the salphen unit (Figure 2d,e). These binding energies are consistent with literature‐reported salen‐based polymers [46]. Similarly, *Im‐POP* exhibited similar C 1s, N 1s, and O 1s XPS spectra, confirming the presence of an imine bonding environment in the POP and consistent with the literature values (Figure 2f–h) [44]. Powder X‐ray diffraction patterns of both POPs displayed broad diffraction signals in the range of 5°–50°, suggesting their amorphous nature (Figure S3) [47]. Thermogravimetric analysis indicated high thermal stability; *Sal‐POP* exhibited negligible weight loss up to 350 °C, while *Im‐POP* exhibited relatively low thermal stability (Figure S4). The POPs were degassed at 120 °C prior to performing the nitrogen (N_2_) adsorption measurements. N_2_ adsorption isotherms at 77 K up to a relative pressure (*P*/*P*_0_) of 1,revealed type‐II uptake profiles for both *Sal‐POP* and *Im‐POP* with a total N_2_ uptake of 16.22 and 29.90 mL g^−1^, respectively (Figures S5 and S6). The type‐II profile indicates the nonporous/macroporous nature of POPs to N_2_. In this case, N_2_ adsorption is not due to micropore filling but rather interparticle pore filling. Application of nonlinear DFT yielded a Brunauer–Emmett–Teller (BET) surface area of 24.15 and 45.61 m^2^ g^−1^ for *Sal‐POP* and *Im‐POP*, respectively. Field emission scanning electron microscope image analysis indicated irregularly fuzed micron‐sized spherical particles for both POPs, which were also observed for literature‐reported POPs (Figures 2i,j and S7) [13, 31]. Energy‐dispersive spectroscopy (EDS) mapping showed the distribution of C, N, and O throughout the polymer network structure (Figures S8–S11). Elemental analysis further confirmed the successful incorporation of nitrogen functionalities within the polymeric frameworks, with *Sal‐POP* and *Im‐POP* exhibiting nitrogen contents of 6.95% and 9.26%, respectively (Table S2). High‐resolution transmission electron microscope images showed aggregated particles at low magnification; however, at higher magnification, irregularly stacked POP layer‐like structures were observed (inset Figures 2i,j and S12).

**FIGURE 1:**
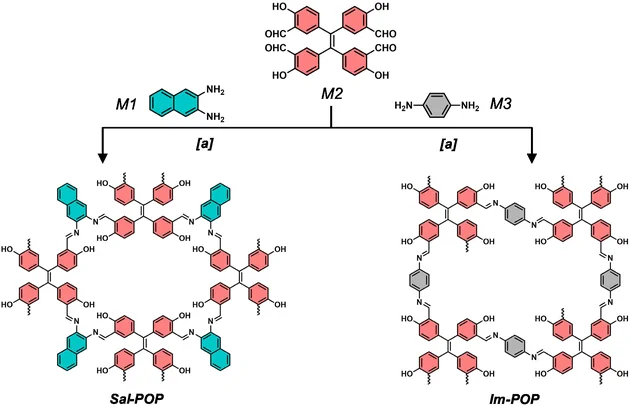
Synthetic route to *Sal‐POP* and *Im‐POP* from corresponding monomers. Reaction conditions: [*a*] *n*‐BuOH/*o*‐DCB/AcOH (1:1:0.2), 120 °C, 96 h.

**FIGURE 2:**
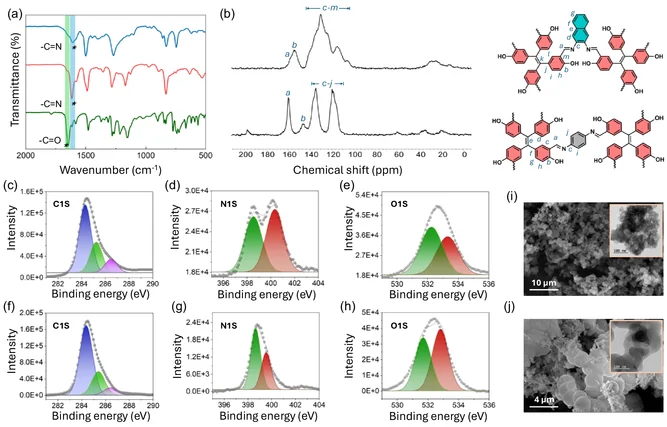
POP characterization: (a) FT‐IR spectra of *Sal‐POP* (blue), *Im‐POP* (red), and *M2* (green), (b) solid‐state ^13^C CP/MAS NMR spectra of *Sal‐POP* (top) and *Im‐POP* (bottom). Deconvoluted XPS spectra of C 1s, N 1s, and O 1s for *Sal‐POP* (c–e) and *Im‐POP* (f–h). SEM image of *Sal‐POP* (i) and *Im‐POP* (j) with corresponding HRTEM images shown in the inset.

### 2.2 Uranium Capture of *Sal‐POP* and *Im‐POP*

Interestingly, both POPs showed remarkable UO_2_^2+^ capture properties under simulated seawater conditions across a wide concentration range (10–100 ppm) owing to the presence of high‐density coordinating salphen or *o*‐hydroxy imine sites. The uptake was monitored using a calibration curve obtained by titrating arsenazo dye III with known UO_2_^2+^ concentration, a well‐established method in literature (Figure S13) [28, 48, 49]. Uranium capture experiments were performed by dispersing 5 mg of POP in 400 mL of uranium‐spiked simulated seawater (pH maintained at 2.5) and stirring the dispersion at 298 K for 24 h (Equations S1–S3, see Experimental Section for details). As shown in Figure 3a, at a uranyl concentration of 10 ppm, *Sal‐POP* exhibited uranium uptake capacity of 0.114 g g^−1^ within 1 h and reached a maximum of 0.18 g g^−1^ in 24 h. On the other hand, *Im‐POP* showed a uranium uptake capacity of 0.069 g g^−1^ within 1 h, reaching a maximum of 0.23 g g^−1^ after 24 h. Notably, uranium uptake capacities substantially increased upon systematically increasing the UO_2_^2+^ concentration from 10 to 100 ppm (Figure 3b). For instance, at 50 and 100 ppm, *Sal‐POP* exhibited uptake capacities up to 0.76 and 1.05 g g^−1^, respectively, while the *Im‐POP* exhibited uptake capacities up to 0.89 and 1.79 g g^−1^, respectively (Figures S14 and S15). Such high loading capacity of both POPs underscores their strong affinity for uranyl ions through coordination interactions, as well as the accessible porosity of the POPs. Here, it should be noted that the BET surface area determined from the type II N_2_ adsorption isotherm does not reflect the actual uranyl‐accessible surface area of the POPs. Also, it is reported in the literature that the BET surface area is not directly correlated with the small‐molecule uptake properties of POPs. For instance, the lowest BET surface area POPs are known to exhibit considerably high molecular iodine (I_2_) uptake values compared to high surface area POPs [50, 51, 52]. Interestingly, the color of the *Sal‐POP* changed from yellow to black upon uranium adsorption (Figures 3b inset and S16). The distribution coefficient (*K*_d_) reflecting the binding affinity between POP and UO_2_^2+^ was determined to be higher; for instance, *Sal‐POP* showed *K*_d_ values of 1.88 × 10^5^, 1.26 × 10^5^, and 0.53 × 10^5^ mL g^−1^ at 10, 50, and 100 ppm uranyl ion concentrations, respectively (Equation S4). On the other hand, *Im‐POP* exhibited *K*_d_ values of 2.94 × 10^5^, 1.56 × 10^5^, and 1.15 × 10^5^ mL g^−1^ at 10, 50, and 100 ppm uranyl ion concentrations, respectively (Figure 3c). The higher *K*_d_ values indicate stronger binding affinity between POP and UO_2_^2+^ and enhanced removal efficiency. *Im‐POP* showed relatively better *K*_d_ values compared to *Sal‐POP* at all UO_2_^2+^ concentrations, indicating its better binding affinity to UO_2_^2+^. This is in conjunction with uranium removal efficiencies, where *Im‐POP* and *Sal‐POP* showed 78%, 66%, 59% and 70%, 61%, and 40% of UO_2_^2+^ removal efficiencies at 10, 50, and 100 ppm UO_2_^2+^, respectively (Figure 3d, Equation S5). Kinetic analysis of the uranium adsorption process was also investigated; the plot of *t/q*_*t*_ versus *t* fitted well with the *pseudo‐second‐order* equation model (Equations S6 and S7) for the UO_2_^2+^ adsorption process [28], indicating that chemisorption is the rate‐limiting step (Figures 3e, S17 and, S18). Moreover, the adsorption isotherms fitted well with the *Langmuir* adsorption model (Equations S8 and S9), consistent with monolayer adsorption behavior and a uniform distribution of active binding sites (Figure S19) [30, 53]. The uranyl uptake properties were further compared with structurally similar pTPE‐Salphen [30]. As shown in Figure S20, the pTPE‐Salphen exhibited a uranium uptake value of 119.3 mg g^−1^ for 10 ppm, which is lower than *Sal‐POP* and *Im‐POP* over the same duration. These results clearly confirm that the uranyl uptake properties of the *Sal‐POP* porous network are not dictated solely by monomer *M2* but also by the 3D connectivity and pore topology resulting from the naphthyl‐diamine. Collectively, these results demonstrate that both *Sal‐POP* and *Im‐POP* exhibit rapid, high‐capacity uranium capture directed by the suitable salphen, *o*‐hydroxy imine chelating sites distributed on the three‐dimensionally accessible pore surface of a POP network [14, 37]. It is worth mentioning that the UO_2_^2+^ adsorption capacities are relatively higher than other POPs, including COFs containing pore‐blocking amidoxime groups reported in literature (Table S3, Scheme 1). The strategy used here avoids the postsynthetic modification procedure, and the coordinating sites are easily accessible to uranium for adsorption and desorption [54, 55].

**FIGURE 3:**
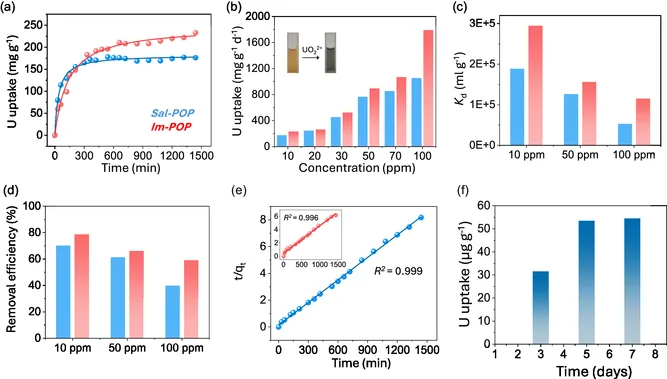
Uranyl cation capture behavior of *Sal‐POP* (blue) and *Im‐POP* (red): (a) time dependent UO_2_^2+^ uptake capacity of POPs with 10 ppm uranium solution (pH = 2.5), (b) uptake capacity of POPs as a function of UO_2_^2+^ concentration in the range of 10–100 ppm (pH = 2.5), inset shows the color changes of *Sal‐POP* upon UO_2_^2+^ adsorption. (c) Distribution coefficient (*K*_d_) at varying uranyl concentrations for *Sal‐POP* (blue) and *Im‐POP* (red) and (d) corresponding removal efficiencies. (e) Time‐dependent uranium uptake by POPs fitted to *pseudo‐second‐order* kinetic model for *Sal‐POP* (blue) and *Im‐POP* (red, inset). (f) Uranium uptake performance of *Sal‐POP* at 6.3 ppb uranyl‐spiked simulated seawater.

To further demonstrate the practical applicability of the POPs for uranium extraction under practical conditions, the uranyl uptake experiments were conducted at ppb level. For this, 5 mg of *Sal‐POP* was continuously stirred in 400 mL of 6.3 ppb uranyl in simulated seawater for 7 days, and the uranyl uptake was monitored by inductively coupled plasma mass spectrometry analysis. As seen in Figure 3f, the *Sal‐POP* showed an uptake of 31.6 μg g^−1^ in 3 days, increasing to a maximum of 54.48 μg g^−1^ in 7 days. These values are comparable to those reported in the literature [49, 56],where experiments were performed with smaller volumes of seawater. These results confirmed that the POPs can exhibit uranyl‐extraction properties even at ppb levels of uranyl ions under seawater conditions. After this, the uranyl uptake capacities of POPs were assessed using natural seawater collected from Digha Beach, West Bengal, India. After continuous stirring of *Im‐POP* in 400 mL of natural seawater for 8 days, the uranyl concentration decreased from 0.666 to 0.349 ppb, corresponding to a uranium uptake capacity of 25.36 μg g^−1^ (Figure S21).

### 2.3 Ground–State Complex Formation of POP With UO_2_^2+^

Ground–state complex formation between POP and UO_2_^2+^ is essential to achieve visual color changes during UO_2_^2+^ capture and release cycles with POPs. The ground‐state color changes are generally studied by UV absorption spectroscopy; unfortunately, the POPs under study are generally insoluble in common organic solvents and do not exhibit appropriate UV absorption spectra in their dispersed state. Therefore, to demonstrate ground‐state complexation experimentally, Stern–Volmer quenching analysis combined with the excited‐state lifetimes of uranyl‐bound POPs was used. As shown in Figure S22, *Sal‐POP* exhibited a strong yellow emission ($\lambda_{\mathrm{em}}^{\max}$ = 578 nm) in DMF and hypsochromically shifted in nonpolar solvents potentially due to ESIPT. Upon addition of aqueous UO_2_^2+^ (250 μM) to the dispersion of *Sal‐POP* (0.5 mg mL^−1^), the fluorescence was quenched significantly with a quenching efficiency >80%, potentially due to UO_2_^2+^ binding to the coordinating salphen units of *Sal‐POP* (Figure 4a). The fluorescence titration experiments were carried out by gradual addition of aq. UO_2_^2+^ (0–250 μM) also resulted in gradual fluorescence quenching (up to 85%) (Equation S10). The plot of *F*_0_ */F* versus [UO_2_^2+^] revealed a nonlinear Stern–Volmer response at higher UO_2_^2+^ concentrations indicating combined static and dynamic quenching pathway [57] where the UO_2_^2+^ binds to the salphen‐unit present in the POP, forming a dark ground state complex (Figure 4b). The optical response was also evident to the naked eye under 365 nm UV illumination, where the characteristic yellow emission of *Sal‐POP* visibly faded in presence of UO_2_^2+^ ions. Interestingly, linear Stern–Volmer response was observed at low concentrations static quenching pathway at the lower UO_2_^2+^ concentrations. The static quenching pathway can be represented by the Stern–Volmer Equation (1) [58],

**FIGURE 4:**
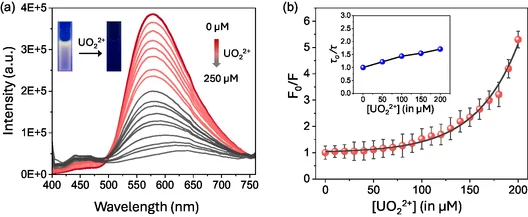
Uranyl‐induced fluorescence quenching of *Sal‐POP*: (a) Emission spectra of *Sal‐POP* recorded upon incremental addition of UO_2_^2+^ (*λ*_ex_ = 390 nm) and (b) variation of *F*_0_ */F* with increasing UO_2_^2+^ concentration, revealing a nonlinear Stern–Volmer response; inset shows changes in excited state lifetime with the addition of UO_2_^2+^ (0–200 μM).

(1) $$F_{0}/F=1+K_{\mathrm{sv}}\left[Q\right]$$

where *F*_0_, *F* represent the fluorescence intensities before and after the addition of UO_2_^2+^, and *K*_SV_ represents the Stern–Volmer constant. The linear fitting of *F*_0_ */F* versus [UO_2_^2+^] at low UO_2_^2+^ concentration revealed a relatively high *K*_SV_ value of 9.88 × 10^6^ M^−1^, indicating a strong interaction between the uranyl ions and the salphen coordination sites within the POP network (Figure S23). Furthermore, the Benesi–Hildebrand plot yielded an association constant (*K*_a_) of 1.05 × 10^5^ M^−1^ for the UO_2_^2+^–POP complex (Figure S24), underscoring the high binding affinity and effective host–guest interaction between uranyl and POP. Excited‐state lifetime measurements further confirmed the static quenching pathway via UO_2_^2+^ complexation to the Salphen unit of POP. As shown in the *τ*_0_ */τ* versus [UO_2_^2+^] plot, the excited‐state lifetime of *Sal‐POP* upon addition of UO_2_^2+^ ions (0–200 μM) did not change significantly at lower concentrations, indicating the formation of a dark‐ground state complex (inset Figures 4b and S25–S27); however, a positive deviation from linearity was observed at higher concentrations. These results suggest that at lower concentrations, fluorescence quenching is primarily governed by static complexation between UO_2_^2+^and the salphen binding sites, whereas at higher concentrations, a combination of static and dynamic quenching [36, 59] occurs potentially due to interchain excited‐state energy transfer from fluorescent TPE‐salphen units to the dark UO_2_^2+^‐bound salphen units within the polymeric framework. Interestingly, the *Sal‐POP* showed minimal PL quenching with other metal ions, except Cu^2+^ (Figures S28 and S29). The PL quenching of salphen‐based POPs in the presence of Cu^2+^ was also observed in our previously reported POP [31]. These results confirm that UO_2_^2+^ indeed binds with the salphen unit of the POP and is responsible for the visual color changes. The fluorescence quenching response of *Sal‐POP* in simulated seawater was also appreciable for UO_2_^2+^ among other metal ions. Furthermore, *Sal‐POP* showed appreciable quenching response to UO_2_^2+^ in natural seawater (Figure S30). Unlike *Sal‐POP*, *Im‐POP* is nonfluorescent and not suitable for fluorescence sensing of UO_2_^2+^. The fluorescence spectra and the images of *Im‐POP* in different solvents under UV light are shown in Figures S31 and S32.

### 2.4 Validation of U^VI^ State in UO_2_^2+^@POP

To validate the U^VI^ state of UO_2_^2+^@POP, XPS, EDS, and FT‐IR measurements were performed on the uranium‐loaded POPs, namely, UO_2_^2+^@*Sal‐POP* and UO_2_^2+^@*Im‐POP*. The binding of UO_2_^2+^ was initially confirmed by XPS analysis (Figures 5a, S31, and S32); both UO_2_^2+^@*Sal‐POP* and UO_2_^2+^@*Im‐POP* showed peaks corresponding to uranium. The deconvoluted U 4f spectra showed peaks corresponding to U 4f_7/2_ and U 4f_5/2_ at ~381.39 and ~392.05 eV, respectively, confirming the successful adsorption of uranium in its +6 oxidation state [28]. Similar U 4f spectra were observed for UO_2_^2+^@*Im‐POP* [13]. Further, the deconvoluted O 1s spectra revealed UO_2_^2+^ peaks at 530.63 and 531.6 eV for UO_2_^2+^@*Sal‐POP* and UO_2_^2+^@*Im‐POP*, respectively (Figures S33 and S34). Further, the shifted O 1s and N 1s peaks of POPs indicated coordination of UO_2_^2+^ with salphen‐unit and *o*‐hydroxy imine unit of the corresponding POPs [13, 30]. These results confirm chemisorption of uranium on the pore surface of the POPs via chelating coordinating interactions [28]. Also, the EDS analysis revealed a uniform distribution of uranium throughout the POPs, supporting the uniform distribution of the chelating sites present in the POP material (Figures 5b,c, S35, and S36). FT‐IR spectra of UO_2_^2+^@*Sal‐POP* and UO_2_^2+^@*Im‐POP* exhibited characteristic asymmetric stretching vibration of the O═U═O at 905 and 894 cm^−1^, confirming the presence of coordinated UO_2_^2+^ in both POPs (Figures 5d, S37, and S38). These values are consistent with those of other uranium‐adsorbed POPs reported in the literature [30].

**FIGURE 5:**
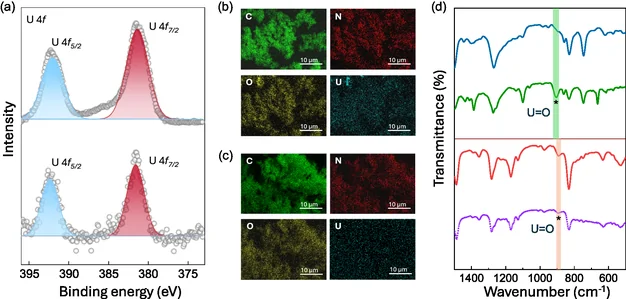
Characterization of UO_2_^2+^ adsorbed POPs: (a) U 4f XPS spectra for UO_2_^2+^@*Sal‐POP* (top) and UO_2_^2+^@*Im‐POP* (bottom). EDS mapping of (b) UO_2_^2+^@*Sal‐POP* and (c) UO_2_^2+^@*Im‐POP*. (d) FT‐IR spectra of *Sal‐POP* (blue), UO_2_^2+^@*Sal‐POP* (green), and *Im‐POP* (red), UO_2_^2+^@*Im‐POP* (violet).

### 2.5 Computational Analysis of UO_2_^2+^ Binding

To support the UO_2_^2+^ chemisorption on the pore surface of POPs via coordination interactions, density functional theory (DFT) calculations were performed by optimizing the elementary unit of both POPs. As seen in Figure S39, both *Sal‐POP* and *Im‐POP* exhibit intramolecular H‐bonding between the corresponding phenolic OH and ─C═N moiety of salphen and *o*‐hydroxy imine units present, respectively. The calculated binding energy of the UO_2_^2+^ to the salphen unit in UO_2_^2+^@*Sal‐POP* and to the *o*‐hydroxy imine in UO_2_^2+^@*Im‐POP* were calculated to be −67.10 and −71.94 kcal mol^−1^, respectively (Figure 6a). The relatively higher exergonic binding energy for UO_2_^2+^@*Im‐POP* signifies superior thermodynamic stability and stronger coordination interactions between the uranyl cation and *o*‐hydroxy imine moiety. These computational findings further corroborate the experimental observations, specifically the enhanced uranium uptake capacity observed for *Im‐POP* relative to *Sal‐POP*. Charge density difference iso‐surfaces further elucidate the nature of this coordination, revealing significant charge accumulation around the bound uranyl center and a corresponding delocalized charge depletion across the POP frameworks (Figures 6b and S40). Furthermore, the band decomposed charge density surfaces for the frontier molecular orbitals (HOMO − 1, HOMO, LUMO, and LUMO + 1), as depicted in Figures 6c–f and S41, highlight a robust *f*–π conjugation [60]. This electronic overlap confirms the strong covalent character and orbital interactions that drive the efficient sequestration of uranium within these systems.

**FIGURE 6:**
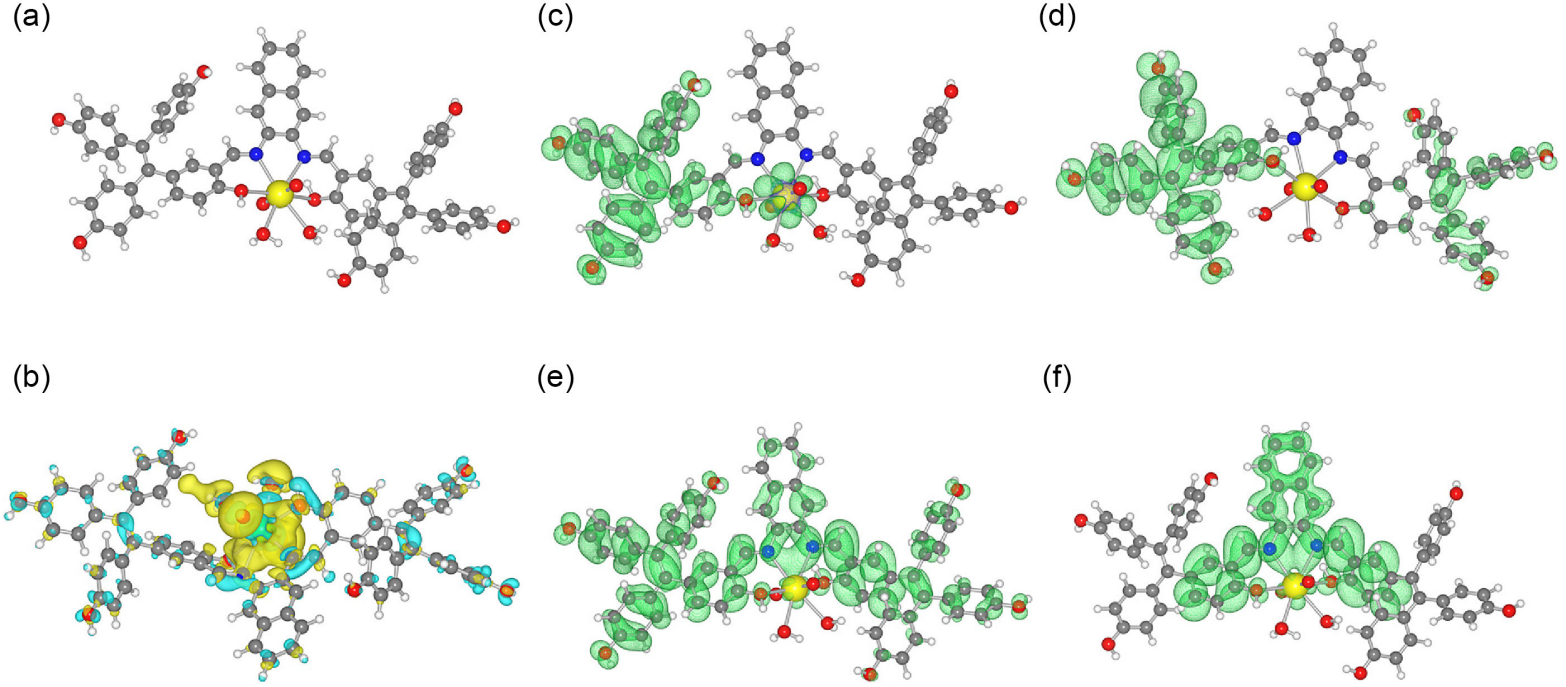
DFT analysis of uranium binding in *Sal‐POP*: (a) Optimized geometry of UO_2_^2+^@*Sal‐POP*, (b) charge density difference iso‐surface of UO_2_^2+^@*Sal‐POP* (yellow: positive and cyan: negative). (c–f) Band decomposed charge density iso‐surface of HOMO − 1, HOMO, LUMO and LUMO + 1, respectively, for UO_2_^2+^@*Sal‐POP*.

### 2.6 pH Dependency and Selectivity

To understand the effect of pH on the uranyl uptake properties, pH‐dependent uranyl capture properties have been studied. As shown in Figure 7a, the uranyl uptake (*q*_*t*_) was higher at a low pH of 2.5 and gradually decreased with increasing pH to 9. These results indicate that the uranyl‐uptake properties of POPs are strongly pH‐dependent, with the lowest pH optimal for achieving high uptake capacities. Such high uptake in a low‐pH environment of the *Sal‐POP* indicates the applicability of these POPs for uranium extraction from highly acidic nuclear wastewater (pH < 4) [61]. To further evaluate the impact of uranyl uptake properties of *Sal‐POP and Im‐POP* at 10 ppm in the presence of competing ions, uranium uptake experiments were conducted in simulated seawater containing representative coexisting metal ions of Cu^2+^, VO_3_^−^, Fe^2+^, Ni^2+^, Ca^2+^, and Mg^2+^ at concentration ratios representative of natural seawater [8]. As shown in Figures 7b, S42, and S43, both *Sal‐POP* and *Im‐POP* exhibited appreciable UO_2_^2+^ uptake in the presence of interfering ions. Among the ions, Cu^2+^ showed the highest interference, while Mg^2+^ showed the lowest. Similar interference results were also reported for other POPs in the literature [62].

**FIGURE 7:**
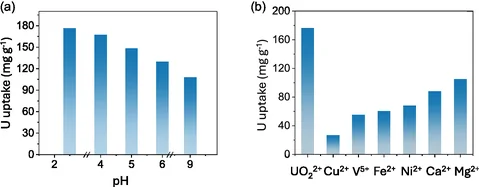
(a) pH dependent uranyl (UO_2_^2+^) uptake properties of *Sal‐POP*. (b) Uranyl (UO_2_^2+^) uptake of *Sal‐POP* in presence of interfering metal ions.

### 2.7 Saturation Indication, and Recyclability

Notably, *Sal‐POP* enables naked‐eye visualization of uranyl adsorption/desorption cycles with distinct colorimetric changes. Upon exposure to UO_2_^2+^ at 250 μM, the characteristic yellow color of *Sal‐POP* gradually fades and ultimately transforms to deep black upon saturation (Figure 8a). This reversible color change arises from strong ground–state complex formation between UO_2_^2+^ and the salphen units, providing a clear visual endpoint that directly signals uranyl saturation without the need for instrumental analysis. The color changes of *Sal‐POP* specific to UO_2_^2+^ were also evident from the daylight images of *Sal‐POP* with different metal ions (Figure S16). No such color changes were observed with *Im‐POP* due to a differing electronic environment from *Sal‐POP* (Figure S44). Moreover, the initiation of the UO_2_^2+^ adsorption process was trackable by simple fluorescence turn‐off and its highly reversible. To evaluate the operational stability of the POPs, recyclability experiments were carried out under simulated seawater conditions [35]. After each adsorption run, the uranium‐loaded materials, that is, UO_2_^2+^@*Sal‐POP* and UO_2_^2+^@*Im‐POP* were regenerated by treatment with 2 M Na_2_CO_3_ solution (60 mL) and gently stirred for 24 h. The suspensions were filtered, repeatedly washed with distilled water until the filtrate became neutral, and then dried under vacuum. The regenerated POPs were then subjected to a subsequent adsorption cycle under identical conditions. Both *Sal‐POP* and *Im‐POP* exhibited excellent cycling stability; over five consecutive adsorption–desorption cycles, *Sal‐POP* retained an adsorption efficiency exceeding 88%, while *Im‐POP* maintained an adsorption efficiency of approximately 86%, indicating minimal degradation of the coordinating sites and preserved pore accessibility (Figures 8b and S45). Similarly, the fluorescence response of *Sal‐POP* toward UO_2_^2+^ was also largely preserved across repeated cycles (Figure 8c). FT‐IR spectra collected before and after cycling showed no significant structural changes, further demonstrating the robustness of the POP frameworks under repeated chemical regeneration (Figures S46 and S47) [30, 36]. These results highlight the excellent reusability of both POPs and underscore the durability of the preorganized salphen and *o*‐hydroxy imine binding motifs. The ability to regenerate the POP adsorbents under mild carbonate washing conditions, without loss of adsorption capacity or photophysical response, is particularly advantageous for practical applications in continuous uranium monitoring and remediation. Such high reversibility makes a clear distinction between POPs and small‐molecule salen compounds. Although salen‐based uranyl complexes are known, they often exhibit 1:1 stoichiometry of uranyl to salen [63]. Therefore, they must be used in large quantities for uranyl extraction, which makes these small‐molecule salens inefficient compared to POPs. Also, the molecular salen‐uranyl complexes are soluble in aqueous media and require specialized methods for recycling, thereby increasing the operational and regeneration costs. In contrast, POPs are nanoporous, high‐surface‐area materials containing a high density of uranyl‐binding sites distributed uniformly throughout the 3D network, thereby significantly increasing uranyl uptake capacity in relatively small POP adsorbent materials (ca. 5 mg) compared with stoichiometric small‐molecular salen complexes. POPs are chemically robust due to strong C─C/N linkages; thus, removal of uranyl from the POP network is simple. They are highly recyclable due to their insolubility in seawater or organic solvents, highlighting their potential practical applications in large‐scale seawater uranium extraction.

**FIGURE 8:**
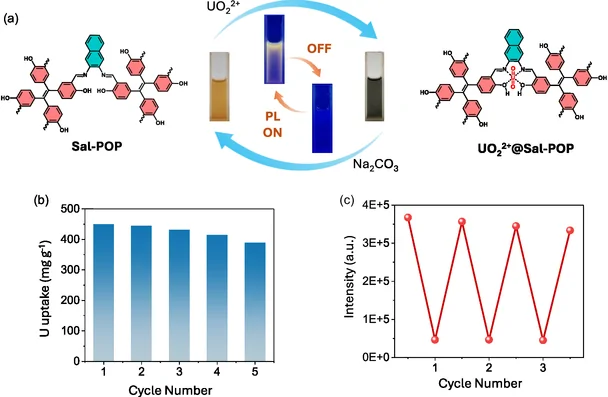
(a) Schematic illustration of *Sal‐POP* regeneration and uranyl extraction, (b) UO_2_^2+^ adsorption capacity of *Sal‐POP* in five successive adsorption–desorption cycles, and (c) reusable performance of *Sal‐POP* for selective uranyl sensing and recovery.

## 3 Conclusion

In summary, the study demonstrated postsynthetic modification‐free, self‐indicative POPs as a unique type of uranium adsorbents that exhibit high‐capacity uranyl capture. *Sal‐POP* showed a remarkable uranyl uptake capacity of 0.114 g g^−1^ h^−1^ with a high distribution coefficient, *K*_d_ = 1.88 × 10^5^ mL g^−1^ at 10 ppm UO_2_^2+^. Comparison studies with *Im‐POP* confirmed the role of hydroxy‐imine units in UO_2_^2+^ binding to the POP frameworks. Interestingly, the uptake capacity was 0.069 g g^−1^ h^−1^ with *K*_d_ of 2.94 × 10^5^ mL g^−1^ at 10 ppm UO_2_^2+^. Computational analysis also revealed the strong binding affinity of POPs toward UO_2_^2+^. Both POPs exhibited remarkable recyclability of the uranium capture and release process. Their visible color changes allowed tracking of uranium adsorption initiation, saturation, and desorption, making them unique among POP‐based uranium adsorbents and providing a new platform of self‐indicative uranium adsorbents that are highly suitable for practical, real‐world uranium monitoring and extraction applications.

## Funding

This study was supported by Anusandhan National Research Foundation (SRG/2023/000062) and Indian Institute of Technology Kanpur (IITK/CHM/2022233).

## Conflicts of Interest

The authors declare no conflicts of interest.

## Supporting information

Supplementary Material

## Data Availability

The data that support the findings of this study are available in the Supporting Information of this article.
