# Supplementary material for: Visualizing Reversible Uranyl (UO2 2+) Capture and Release With Self‐Indicating Porous Organic Polymers
Source: Small Sci. 2026 Oct 5;6(10):e70416. doi: 10.1002/smsc.70416 (PMC13639394; doi:10.1002/smsc.70416)
Supplement: Supplementary file 1 [file SMSC-6-e70416-s001.pdf]

Supporting Information

Visualizing Reversible Uranyl (UO_2^{2+}) Capture and Release with Self-Indicating Porous Organic Polymers

*Nilojyoti Sahoo†, Laraib Akhtar†, Rameshwar Rai, Srinivasu Kancharlapalli, and Venkata Suresh Mothika**

†NS and LA contributed equally to this work

N. Sahoo, L. Akhtar, R. Rai, V. S. Mothika

Department of Chemistry, Indian Institute of Technology (IIT) Kanpur, Kanpur 208016, India

*E-mail: smothika@iitk.ac.in

S. Kancharlapalli

Chemistry Division, Bhabha Atomic Research Centre, Mumbai, 400085, India

Homi Bhabha National Institute, Mumbai, 400094, India

1. Experimental methods

1.1 Materials

Naphthalene-2,3-diamine, *p*-phenylenediamine, 4,4′-dihydroxybenzophenone, titanium tetrachloride ($TiCl_4$), boron tribromide (BBr_3), and hexamethylenetetramine (HMTA) were procured from Spectrochem. *n*-Butanol, *o*-dichlorobenzene (*o*-DCB), bromoethane (EtBr) and anhydrous triethylamine (Et_3N) were also obtained from Spectrochem, while zinc dust, potassium carbonate (K_2CO_3), trifluoroacetic acid (TFA), hydrochloric acid (HCl) and acetic acid (CH_3COOH) were purchased from Rankem. Chloride salts of Fe^{2+}, Hg^{2+}, Co^{2+}, Mg^{2+}, Ca^{2+}, Zn^{2+}, Mn^{2+}, Cd^{2+}, Ni^{2+}, and Cu^{2+}, V^{5+} and uranyl nitrate hexahydrate [$UO_2(NO_3)_2 \cdot 6H_2O$] were obtained from SRL Chemicals. Arsenazo III dye was obtained from TCI Chemicals. All chemicals, reagents, and solvents were of analytical grade and used as received without further purification. The anhydrous solvents, including *N,N′*-dimethylformamide (DMF), dichloromethane (CH_2Cl_2), tetrahydrofuran (THF), were freshly distilled under nitrogen following standard purification procedures. TPE-based monomer *M2* was synthesised by adopting the literature procedure.[1–4]

1.2 Characterisations

Stock solutions of metal salts were prepared in distilled water using the chloride or nitrate salts of Na^+, K^+, Mg^{2+}, Ca^{2+}, V^{5+}, Co^{2+}, Hg^{2+}, Cu^{2+}, Mn^{2+}, Cd^{2+}, Ni^{2+}, Fe^{2+}, $Zn^{2+,}$ and UO_2^{2+}, with subsequent dilutions carried out using distilled water. Solution-state ^1H NMR spectra were recorded on a JEOL ECX 400 MHz spectrometer using deuterated chloroform ($CDCl_3$) or dimethyl sulfoxide (DMSO-d^6) as solvents and tetramethylsilane (TMS) as an internal reference. Solid-state ^{13}C CP/MAS NMR spectra were obtained on a Bruker Avance NEO 400 MHz spectrometer and analysed using MestReNova software. FT-IR spectra were measured in the 500–4000 cm^{-1} range on a PerkinElmer 1000 UATR instrument operated in ATR mode. Thermogravimetric analysis (TGA) was performed under a nitrogen atmosphere using a Mettler Toledo TGA/DSC 1 Star System from 25 to 800 °C at a heating rate of 10 °C min^{-1}. Powder X-ray diffraction (PXRD) patterns were collected with a PANalytical X'Pert diffractometer. The surface morphology was examined by field-emission scanning electron microscopy (FE-SEM, FEI NOVA NANO SEM 450) after drop-casting ethanol dispersions of the POPs onto 'Al' foil. Transmission electron microscopy (TEM) images were obtained using an FEI Tecnai G2 12 Twin 120 kV microscope by drop-casting ethanolic dispersions of POPs onto carbon-coated copper grids. pH analysis were performed using Eutech pH 700 instrument. Metal ion concentrations were determined by inductively coupled plasma mass spectrometry (ICP–MS) using an iCAP Q instrument. The elemental content (C, H, and N) was quantified on a

UNICUBE organic elemental analyzer. Nitrogen adsorption–desorption isotherms were recorded at 77 K on an AUTOSORB-iQ analyser after degassing the samples at 120 °C for 12 h. Photoluminescence (PL) spectra were collected using a Horiba Fluoromax-4 spectrofluorometer in dispersed phase. Time-correlated single photon counting (TCSPC) measurements were performed on a custom-built setup equipped with a 405 nm picosecond pulsed diode laser (DeltaFlex, HORIBA Scientific). Solid-state diffuse reflectance UV–vis spectra were recorded on a JASCO V-770 spectrophotometer, while solution-state UV–vis absorption spectra were obtained using a JASCO UV–vis–NIR double-beam spectrometer. X-ray photoelectron spectroscopy (XPS) measurements were performed on a Thermo Scientific NEXSA system, equipped with a monochromatic Al *K*α radiation source (*hv* = 1486.69 eV). All graphical analyses and plots, including fluorescence, adsorption, spectroscopic, and thermal data, were prepared using OriginPro 2022 software.

2. Monomer and POP synthesis.

Monomer *M2* was synthesized according to the literature procedure.[1]

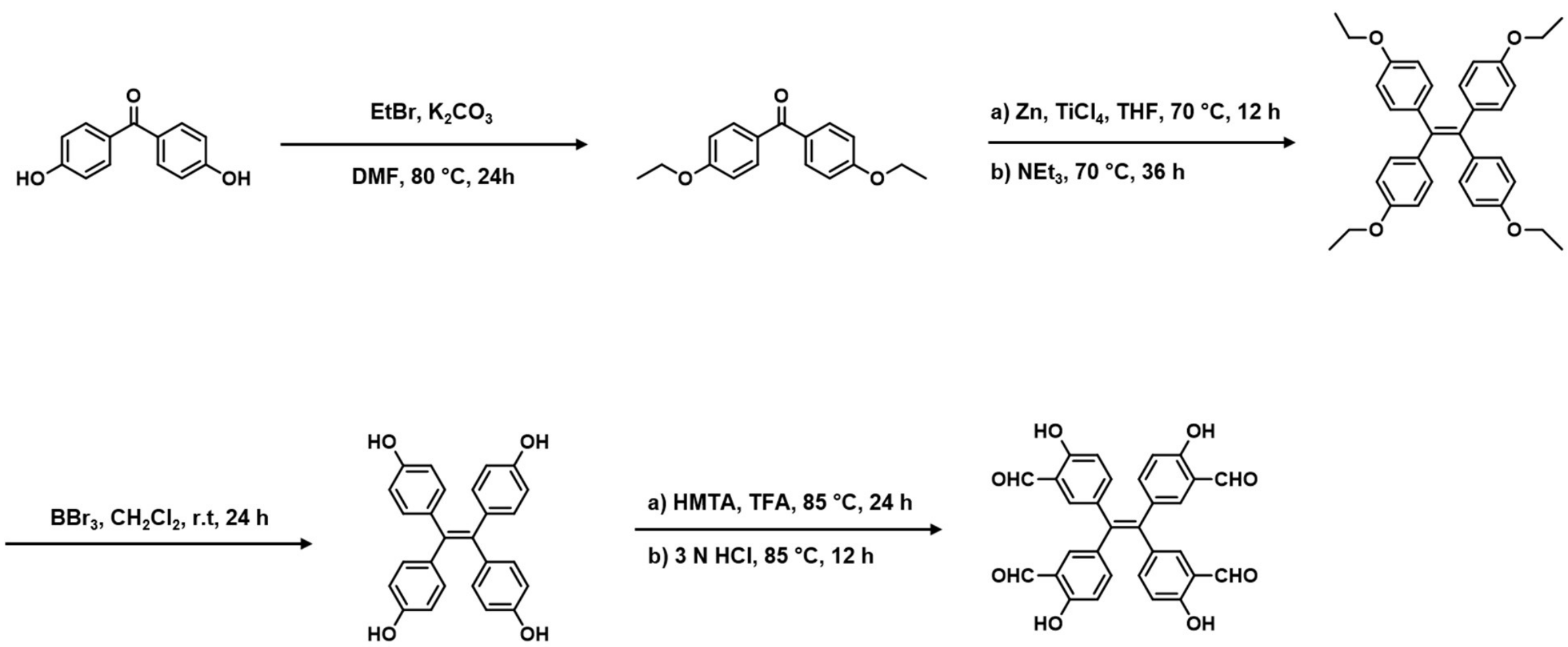

Scheme S1. Synthesis of compound *M2*.[1]

Synthesis of *Sal-POP*: In a *J. Young* Schlenk tube, monomer *M2*[1] (200 mg, 0.39 mmol, 1 eq) and naphthalene-2,3-diamine, *M1* (125 mg, 0.78 mmol, 2 eq) were dissolved in 6 mL of 1:1 (v/v) mixture of *n*-butanol and *o*-dichlorobenzene and added with aq. CH_3COOH (6 M, 0.6 mL). The resulting mixture was sonicated for 20 min at room temperature and subjected to degassing using three freeze-pump-thaw cycles. The reaction mixture was then heated at 120 °C for 4 days. After cooling to room temperature, the precipitated solids were collected by filtration, thoroughly washed with acetone and methanol, and further purified by Soxhlet

extraction with dichloromethane for 24 hours. The solids were further dried at room temperature to obtain the desired POP as a yellow powder. Yield: 78 %

Synthesis of *Im-POP*: In a *J. Young* Schlenk tube, monomer, *M2* (200 mg, 0.393 mmol, 1 eq) and benzene-1,4-diamine, *M3* (85 mg, 0.786 mmol, 2 eq) were dissolved in 6 mL of a 1:1 (v/v) mixture of *n*-butanol and *o*-dichlorobenzene and then added with aq. CH_3COOH (6 M, 0.6 mL). The resulting mixture was sonicated for 20 min at room temperature and subjected to degassing using three freeze-pump-thaw cycles. The reaction mixture was then heated at 120 °C for 4 days. The resulting solid was filtered, washed sequentially with acetone and methanol, and purified by Soxhlet extraction using dichloromethane for 24 h. The solids were further dried at room temperature to obtain the desired POP as an orange powder. Yield: 80 %

3. Uranium capture experiments

3.1 Uranium capture studies

Both *Sal-POP* and *Im-POP* showed excellent uranium capture properties. The stock solution of $UO_2(NO_3)_2{\cdot}6H_2O$ is prepared in distilled water by the addition of 3 M perchloric acid. Here, perchloric acid helps to dissolve the uranyl nitrate and maintain its solubility. From this, uranyl solutions at 10-100 ppm were prepared by adding the stock solution to simulated seawater containing 0.5 M NaCl and 2.3 mM $NaHCO_3$. It should be noted that the uranyl solutions prepared without the addition of perchloric acid resulted in light-green precipitation upon standing for a few hours. Notably, the addition of perchloric acid resulted in a pH of 2.5 for the uranyl-containing simulated seawater.

In a typical uranium capture experiment, 5 mg of each POP was dispersed in 400 mL of uranyl (10-100 ppm) containing simulated seawater, and the final pH was maintained at 2.5. The mixture was stirred for 24 hrs and at predetermined time intervals, aliquots of 1 mL were withdrawn and centrifuged. The filtrate was added to 2 mL of Arsenazo III dye in a cuvette, and UV was recorded. The peak at 651 nm, corresponding to the uranyl-Arsenazo III complex, was monitored to determine the uranyl concentration in the filtrate. During this, the dilution effect must be accounted for to determine the C_0 and C_t values. The uranium concentration of the solution was determined using a UV–visible spectrophotometer based on the Beer–Lambert law, as described by equation S1

$$A = \log\frac{1}{T} = \varepsilon c l \qquad \text{(eq S1)}$$

where A is the absorbance of the solution, T is the transmittance of the solution, ε is the molar absorption coefficient, l is the thickness, and c is the concentration.

The adsorption capacity of the POP samples was determined using equation S2

$$q_t = \frac{(C_0 - C_t) \times V}{m} \qquad \text{(eq S2)}$$

Where C_0 and C_t (mg L^{-1}) represent the initial and uranium concentrations at time t, respectively; V(L) is the volume of the solution; m(g) denotes the mass of the POP sample; and q_t(mg g^{-1}) corresponds to the adsorption capacity at time t.

The adsorption capacity of the POP samples at equilibrium was determined using equation S3

$$q_e = \frac{(C_0 - C_e) \times V}{m} \qquad \text{(eq S3)}$$

Where C_0 and C_e (mg L^{-1}) represent the initial and equilibrium uranium concentrations at time t, respectively; V(L) is the volume of the solution; m(g) denotes the mass of the POP sample; and q_e(mg g^{-1}) corresponds to the adsorption capacity at equilibrium.

3.2 Distribution coefficient

The UO_2^{2+} distribution coefficient was determined to evaluate the affinity of the POPs toward UO_2^{2+} under simulated seawater conditions. In a typical experiment, 5 mg of POP was dispersed in 400 mL of a uranium-containing solution (10 ppm) and stirred continuously for 24 hrs at 298 K. The aliquots (1 mL) were withdrawn at regular time intervals, and the residual UO_2^{2+} concentration in the supernatant was quantified using UV-vis absorption spectroscopy using Arsenazo III method.

The distribution coefficient was calculated according to the equation S4,

$$K_d = \frac{C_0 - C_e}{C_e} \times \frac{V}{m} \qquad \text{(eq S4)}$$

where C_0 and C_e (mg L^{-1}) represent the initial and equilibrium concentrations of UO_2^{2+} respectively; V (L) is the volume of the solution; and m (g) denotes the mass of the POP adsorbent.

The K_d values provide quantitative insight into the interaction strength between the active coordination sites of the POP and the UO_2^{2+} species. A relatively higher K_d value indicates a stronger binding affinity and better extraction UO_2^{2+} efficiency.

3.3 Removal efficiency percentage

To evaluate the uranium removal efficiency, 5 mg of the POP was added to uranium-spiked simulated seawater (400 mL, 10 ppm) and stirred vigorously at room temperature for 24 hours. Aliquots (5 mL) were withdrawn at regular time intervals, and the residual UO_2^{2+} present in the supernatant was determined by UV-vis spectroscopy using Arsenazo (III) dye method.

The uranium removal efficiency (%) was calculated according to the equation S5,

$$\text{Removal efficiency (\%)} = \frac{C_0 - C_e}{C_0} \times 100 \qquad \text{(eq S5)}$$

where C_0 and C_e represent the initial and equilibrium UO_2^{2+} concentrations (mg L^{-1}) in solution, respectively.

3.4 Uranium adsorption kinetics

To understand the adsorption kinetics, 5 mg of each POP was dispersed in 400 mL of simulated seawater containing ~10 ppm UO_2^{2+} ions. The suspension was stirred continuously at 298 K (pH 2.5), and aliquots (1 mL) were withdrawn at regular time intervals for spectrophotometric analysis using the Arsenazo III method based on the Beer–Lambert relationship (eq. S1).

The kinetic behaviour of uranium adsorption was modelled using *pseudo-first-order* and *pseudo-second-order* rate equations. The *pseudo-first-order* model can be expressed as:

$$\log(q_e - q_t) = \log q_e - \frac{k_1}{2.303} t \quad \text{(eq S6)}$$

Whereas the *pseudo-second-order* model can be represented by:

$$\frac{t}{q_t} = \frac{1}{k_2 q_e^2} + \frac{t}{q_e} \quad \text{(eq S7)}$$

where q_t (mg g^{-1}) denotes the amount of uranium adsorbed at time t (min), q_e (mg g^{-1}) is the equilibrium adsorption capacity, and k_1 and k_2 (g mg^{-1} min^{-1}) are the rate constants corresponding to the *pseudo-first order* and *pseudo-second order* models, respectively.

The experimental data were fitted to both kinetic models to elucidate the rate-determining mechanism governing UO_2^{2+} adsorption. In both POP systems, the *pseudo-second-order model* provided better fitting accuracy ($R^2 > 0.99$), indicating that chemisorption, involving surface complexation between UO_2^{2+} and the salphen/hydroxylamine coordination sites, was the dominant adsorption mechanism.

UO_2^{2+} adsorption of POPs at different equilibrium concentrations was fitted to the *Langmuir* and the *Freundlich* isotherm models.

The *Langmuir* isotherm can be expressed by equation S8:

$$\frac{C_e}{q_e} = \frac{1}{K_L q_{\max}} + \frac{C_e}{q_{\max}} \quad \text{(eq S8)}$$

And the *Freundlich* isotherm can be expressed by equation S9:

$$\ln q_e = \ln K_F + \frac{1}{n} \ln C_e \quad \text{(eq S9)}$$

where C_e (mg L^{-1}) represents the equilibrium concentration of UO_2^{2+}, q_e (mg g^{-1}) and $q_{\max}$ (mg g^{-1}) represents the equilibrium and maximum adsorption capacity of POP. The constants K_L and K_F (L mg^{-1}) represent the *Langmuir* and the *Freundlich* binding constants respectively.

3.5 Uranium extraction from natural seawater:

Natural seawater collected from the Digha Beach, West Bengal, India, was used to evaluate the uranium extraction performance of the POP adsorbents under real seawater conditions. The pH of the collected seawater was measured to be 8.23. Prior to the adsorption experiments, the seawater was filtered through a 0.22 µm membrane filter to remove suspended impurities and microorganisms. Subsequently, 5 mg of *Im-POP* was dispersed in 400 mL natural seawater and

stirred continuously at 400 rpm under ambient conditions for 8 days. The uranium concentration in seawater before and after adsorption was determined by ICP-MS analysis. The initial uranium concentration (C_0) in the collected natural seawater from the Digha Beach was determined to be 0.666 ppb by ICP-MS analysis. After continuous stirring with the *Im-POP* adsorbent for 8 days, the uranium concentration decreased to 0.349 ppb (C_t), corresponding to a uranium uptake capacity (q_t) of 25.36 μg g^{-1} under natural seawater conditions.

In addition, low-ppb uranium uptake studies were further carried out under similar experimental conditions using uranyl-spiked simulated seawater containing 6.3 ppb uranium to better mimic ultratrace seawater uranium conditions. *Sal-POP* exhibited appreciable uranium extraction performance under these low-concentration conditions. After continuous stirring for 7 days, the uranium uptake capacity reached 54.48 μg g^{-1}, demonstrating the efficient uranium-capture capability of both POPs even under ultralow ppb-level conditions.

3.6 Fluorescence sensing experiments

Sal-POP exhibited a yellowish-green fluorescence and demonstrated its capability for uranyl ion sensing. In fluorescence quenching experiments, the *Sal-POP* was dispersed and added with an aqueous solution of metal ions, including the uranyl cation (UO_2^{2+}). In a typical procedure, an aqueous solution of metal ion, e.g., UO_2^{2+} (250 μM final concentration), was exposed to *Sal-POP* (0.5 mg mL^{-1}) dispersed in DMF. The fluorescence spectra were recorded before and after the addition of metal ion, and the quenching efficiency (% QE) was calculated using the equation S10:

$$QE\ (\%) = [1 - (F/F_0)] \times 100 \qquad \text{(eq S10)}$$

where F_0 and F correspond to the fluorescence intensities of the *Sal-POP* before and after exposure to metal ion, respectively. The corresponding % QE values of all the metal ions were compared with those of UO_2^{2+} to assess the selectivity of *Sal-POP*.

Fluorescence titration experiments were conducted by gradually adding aqueous UO_2^{2+} solutions (0 – 250 μM) to the DMF dispersion of *Sal-POP* (0.5 mg mL^{-1}) and simultaneously recording the fluorescence spectra. To identify the nanomolar sensitivity of *Sal-POP* to the UO_2^{2+}, individual nanomolar solutions of UO_2^{2+} were exposed to *Sal-POP* and the recorded fluorescence spectra were plotted as F_0/F versus [UO_2^{2+}] to construct Stern–Volmer plots and determine the quenching constants and limits of detection. All fluorescence spectra of *Sal-POP* were obtained by exciting at 390 nm.

3.7 pH Experiments

To evaluate the influence of pH on uranium adsorption, 5 mg of POP sample was dispersed in 400 mL of uranium-spiked simulated seawater (~10 ppm UO_2^{2+}), and the pH of the medium was adjusted to 2.5, 4.0, 5.0, 6.0, and 9.0 using dilute nitric acid (HNO_3) or sodium hydroxide (NaOH) solutions. The suspensions were stirred continuously for 24 h at room temperature to attain adsorption equilibrium.

Subsequently, aliquots were withdrawn, centrifuged, and the residual uranium concentration in the supernatant was quantified by UV–vis spectrophotometry using Arsenazo III dye as the complexing agent. The adsorption capacity (q_t) was calculated using equation S2.

3.8 Selectivity experiments

Ion stock solutions (1 mM) were prepared from the corresponding chloride or nitrate salts of Na^+, K^+, Mg^{2+}, Ca^{2+}, V^{5+}, Co^{2+}, Hg^{2+}, Cu^{2+}, Mn^{2+}, Cd^{2+}, Ni^{2+}, Fe^{2+}, Zn^{2+}, and UO_2^{2+} in distilled water. A *Sal-POP* stock dispersion (0.5 mg mL⁻¹) was prepared in *N,N′*-dimethylformamide (DMF) solvent. Selectivity studies were performed by adding aqueous metal ion solutions (250 μM) to *Sal-POP*, and the resulting quenching efficiencies (%QE) were compared with that of UO_2^{2+}. The addition of UO_2^{2+} (250 μM) to *Sal-POP* (0.5 mg mL⁻¹) produced pronounced fluorescence quenching (up to 85%), attributed to coordination of UO_2^{2+} with salphen sites. Other metal ions exhibit negligible photoluminescence quenching, except Cu^{2+}. However, it is worth noting that the concentration of copper ions in nuclear waste is significantly lower than that of UO_2^{2+},[5,6] thus, *Sal-POP* is suitable for UO_2^{2+} detection in nuclear waste. The limit of detection (LoD) calculated from the linear F_0/F vs [UO_2^{2+}] plot using the equation LoD = 3σ/K, where σ is the standard deviation, indicated a LoD of 7.65 nM, which is notably below the World Health Organisation's permissible uranium concentration limit for drinking water (63 nM).[7]

To further investigate the effect of interfering ions on the UO_2^{2+} uptake capacities, uranyl uptake experiments were performed in simulated seawater containing various coexisting metal ions. In a typical experiment, 5 mg of POP sample was dispersed in 400 mL of simulated seawater containing UO_2^{2+} (10 ppm) along with Cu^{2+} (3 ppm), V^{5+} (5.5 ppm), Fe^{2+} (10 ppm), Ni^{2+} (15 ppm), Mg^{2+} (1290 ppm), or Ca^{2+} (413 ppm), and was stirred for 24 hrs. Here, the interfering ion concentrations were maintained at ratios representative of natural seawater compositions. The suspensions were stirred continuously for 24 h at room temperature, followed by separation of aliquots and determination of the residual uranium concentration by UV–vis spectrophotometry using arsenazo III dye as the complexing agent.

3.9 Recyclability experiments

Recyclability experiments were conducted under simulated seawater conditions using 5 mg of POPs in each adsorption cycle. After uranium adsorption, the uranyl-loaded materials (UO_2^{2+}@*Sal-POP* and UO_2^{2+}@*Im-POP*) were regenerated by treatment with an aqueous Na_2CO_3 solution (2.0 M, 60 mL) and gently stirred at room temperature for 24 h to desorb uranyl carbonate species. The concentration of uranium in the elution solution was quantified by UV–vis spectrophotometry using the Arsenazo dye III method. The regenerated POPs were subsequently collected by filtration and repeatedly washed

with ultrapure water until the supernatant became neutral. The materials were then dried under vacuum and reused for subsequent uranium adsorption experiments under identical conditions. Recycling experiments were performed for five consecutive adsorption–desorption cycles.

4. Computational details

Spin-polarized density functional theory (DFT) calculations were performed using the Vienna ab initio simulation package (VASP).[8–10] Interactions between the valence and core-shell electron were treated by the projector augmented wave (PAW) method.[11] Perdew-Burke-Ernzerhof (PBE) exchange-correlation energy density functional with Grimme's D3 semiempirical method (PBE-D3) was used.[12,13] Plane-wave basis sets with kinetic energy cut-off of 450 eV were used to expand the Kohn-Sham orbital of the valence electrons. For structural optimization, force convergence tolerances were set to 0.01 eV $Å^{-1}$. VESTA package was used to generate the reported figures.[14] Binding energy of the UO_2^{2+} to the *Sal-POP* and *Im-POP* models were measured as

Sal-POP/*Im-POP* + $UO_2^{2+}(H_2O)_5$ → $UO_2^{2+}(H_2O)_n$@*Sal-POP*/*Im-POP* + (5-n)H_2O

$\Delta E = E(UO_2^{2+}(H_2O)_n@Sal\text{-}POP/Im\text{-}POP)+(5-n)\,E(H_2O)-E(Sal\text{-}POP/Im\text{-}POP)-E(UO_2^{2+}(H_2O)_5)$

where $E(UO_2^{2+}(H_2O)_n@Sal\text{-}POP/Im\text{-}POP)$, $E(Sal\text{-}POP/Im\text{-}POP)$ correspond to the energy of $UO_2^{2+}(H_2O)_n$ bound *Sal-POP*/*Im-POP* and pure *Sal-POP*/*Im-POP,* respectively. $E(UO_2^{2+}(H_2O)_5)$ and $E(H_2O)$ are the energies of hydrated uranyl ion (with 5 water) and water molecule respectively. For all the calculations, sufficient vacuum in all the directions has been considered to avoid the image-image interactions and monopole correction to energy is implemented for ionic systems.

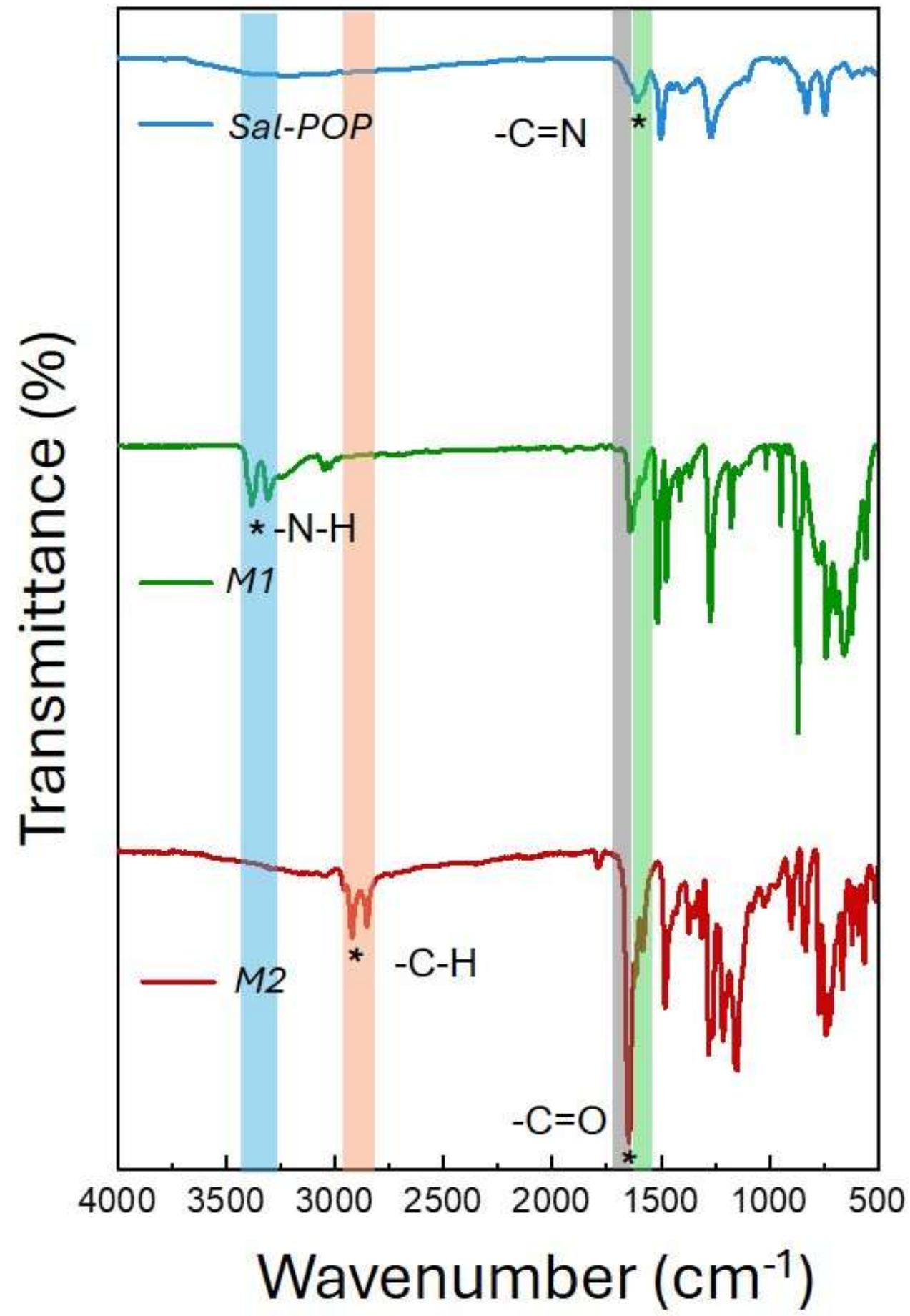

Figure S1. FTIR spectra of *Sal-POP* (blue), compared with *M1* (green) and *M2* (red).

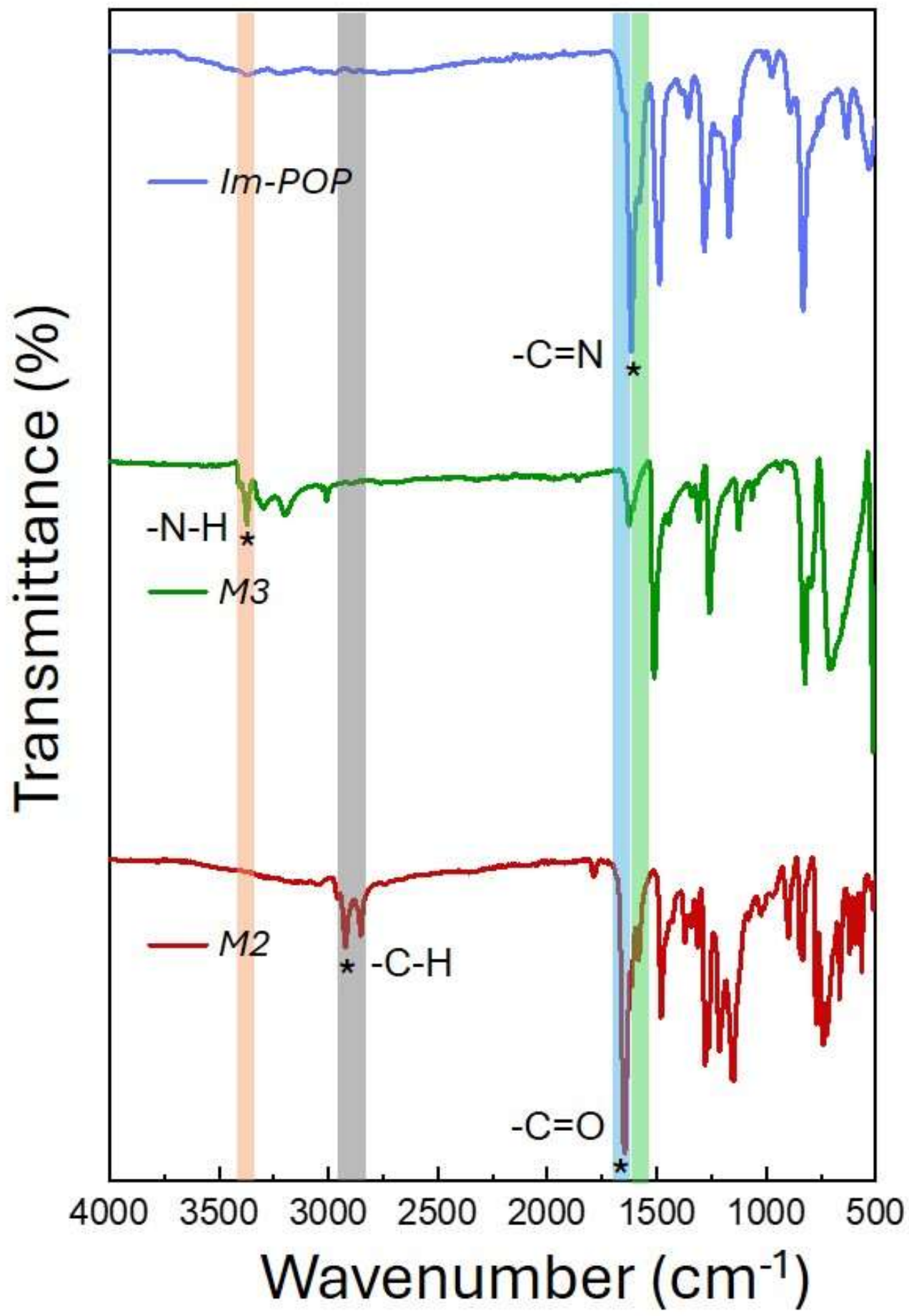

Figure S2. FTIR spectra of *Im-POP* (blue), compared with *M3* (green) and *M2* (red).

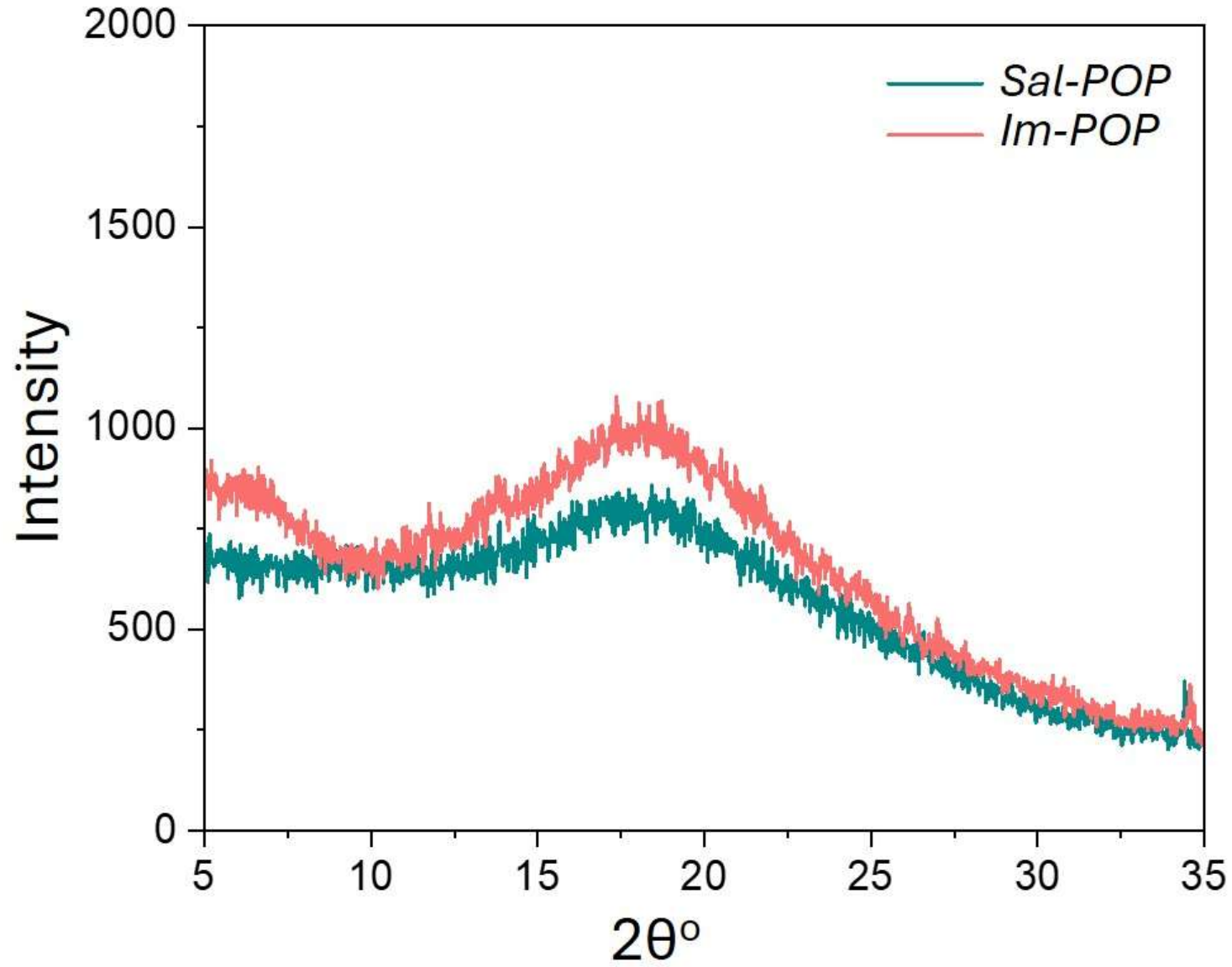

Figure S3. PXRD pattern of *Sal-POP* (green) and *Im-POP* (red).

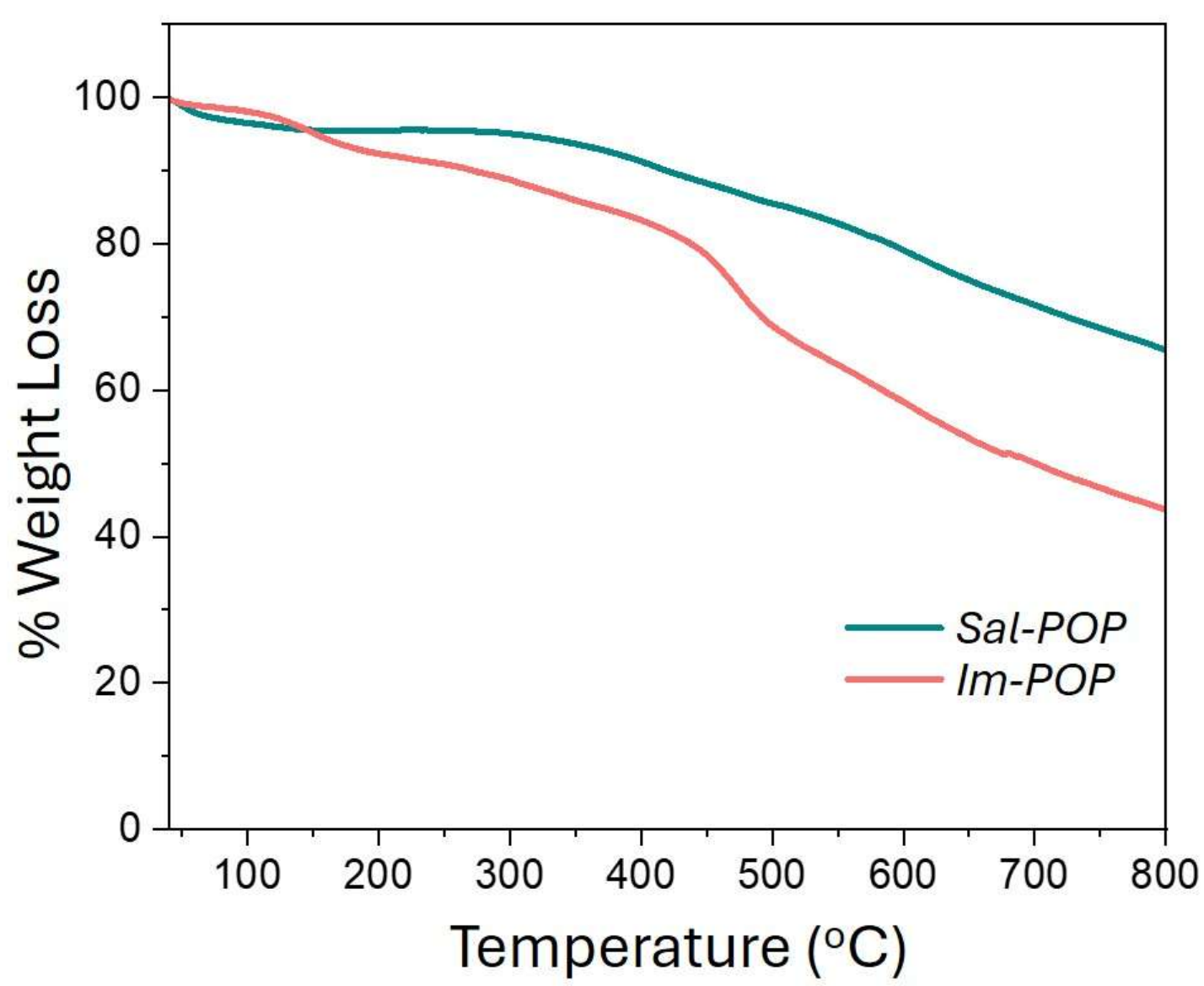

Figure S4. Thermogravimetric analysis (TGA) of *Sal-POP* (green) and *Im-POP* (red).

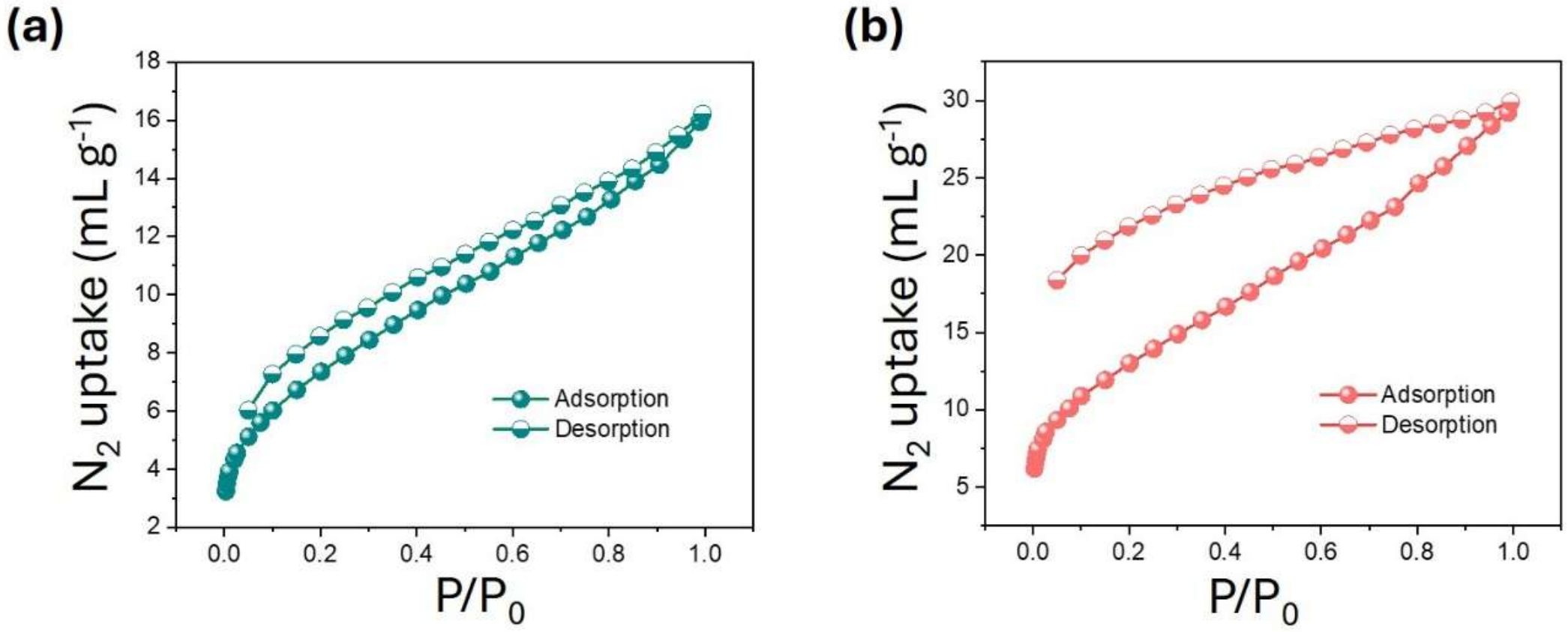

Figure S5. N_2 adsorption-desorption isotherms at 77 K of (a) *Sal-POP* and (b) *Im-POP*.

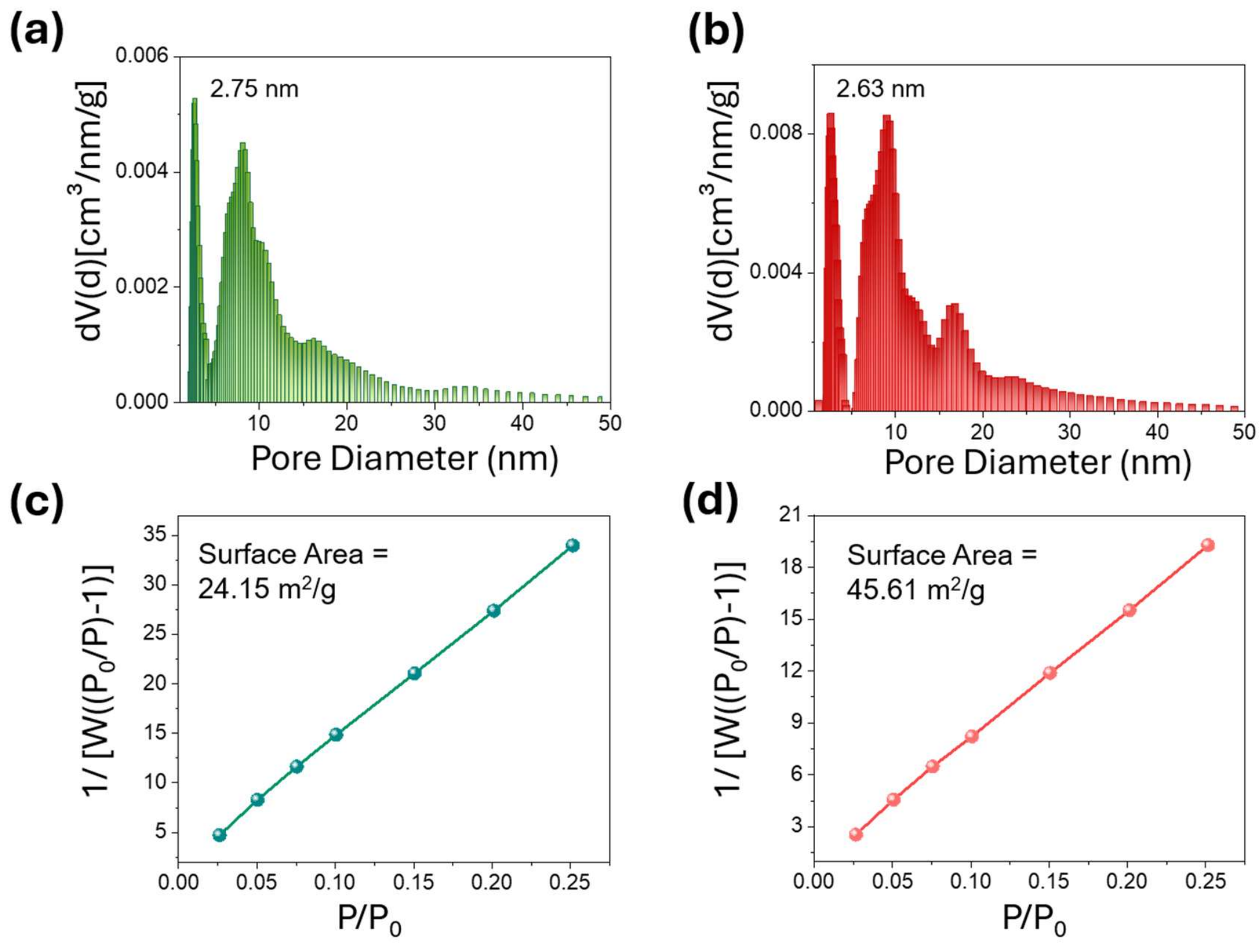

Figure S6. Pore size distribution of (a) *Sal-POP*, (b) *Im-POP*. Determination of BET surface area for (c) *Sal-POP*, and (d) *Im-POP*.

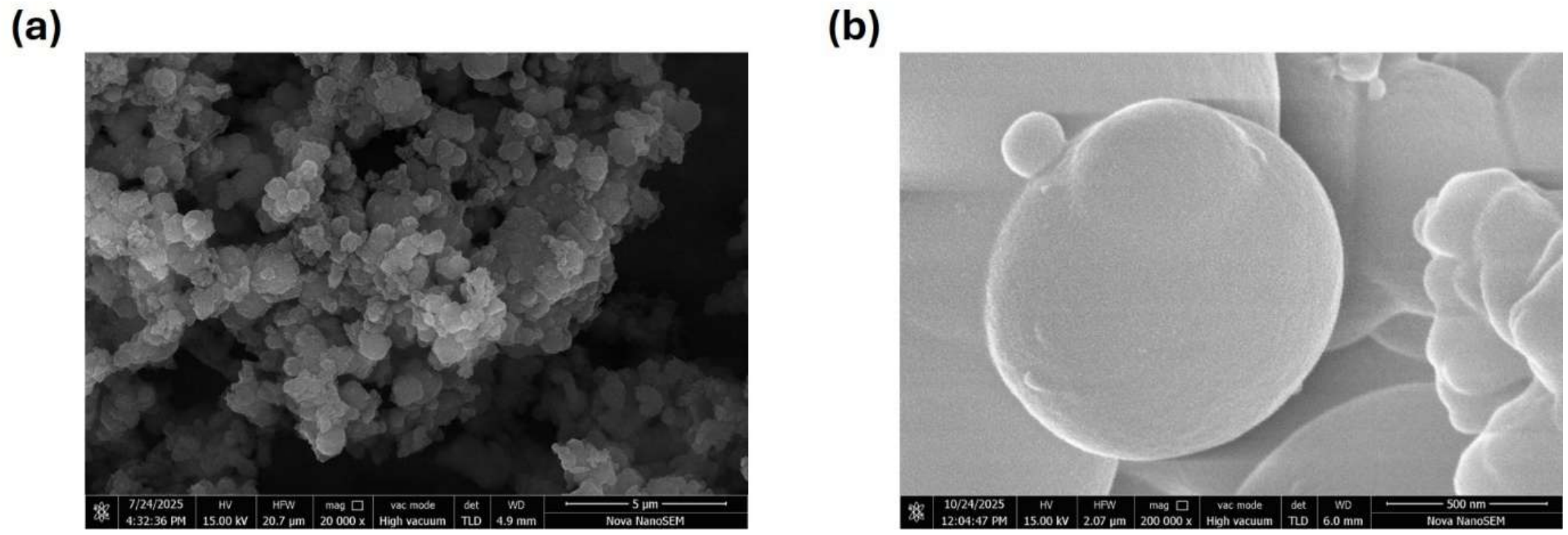

Figure S7. SEM images of (a) *Sal-POP* and (b) *Im-POP*.

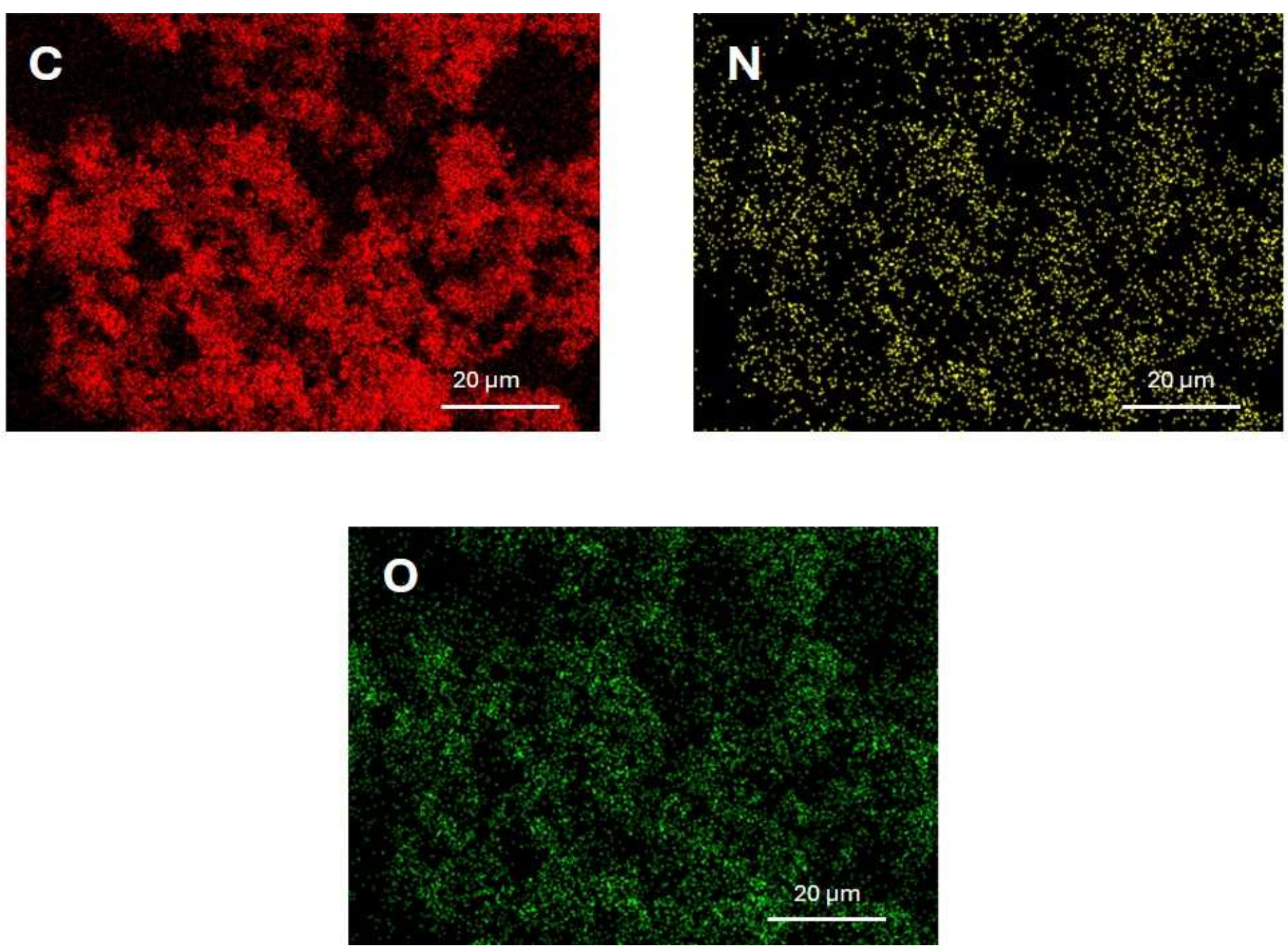

Figure S8. EDS elemental mapping of *Sal-POP* showing distribution of C, N, and O.

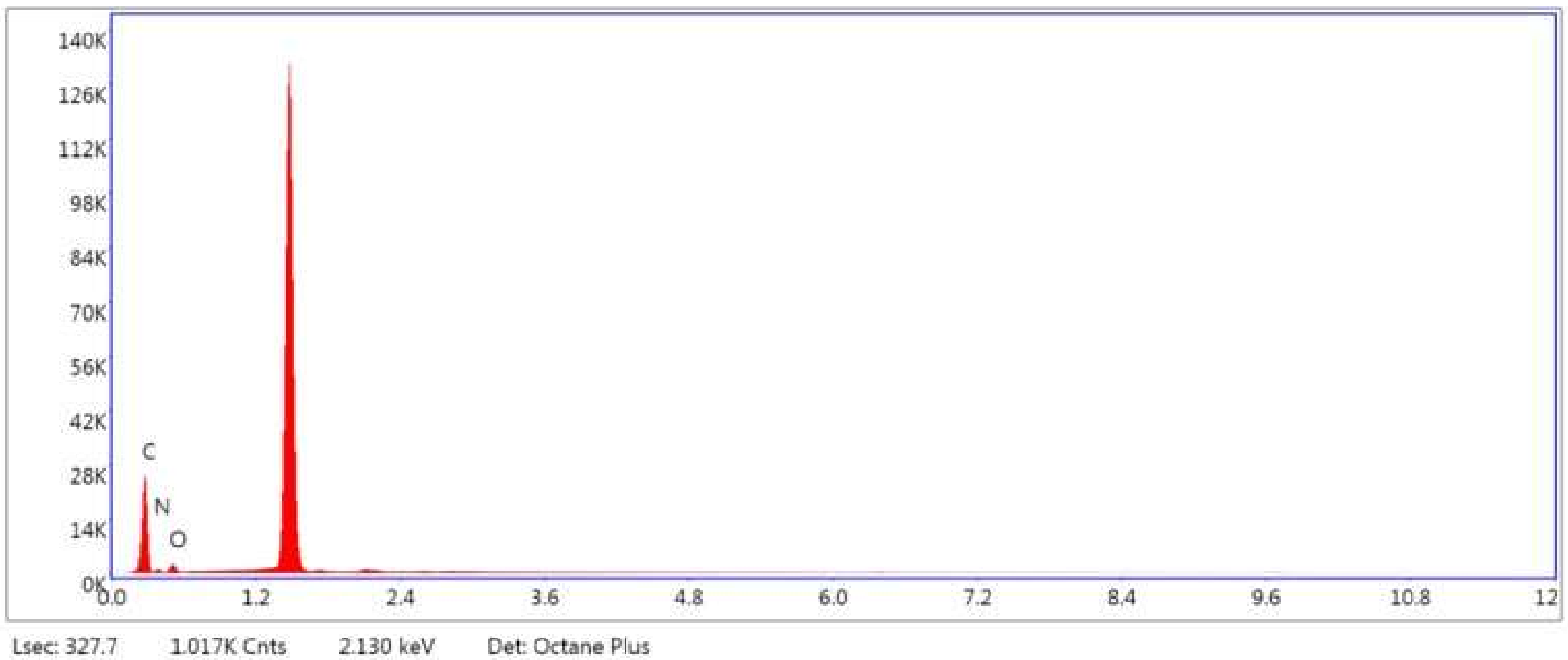

Figure S9. EDAX spectrum of *Sal-POP*. The sample was drop-cast onto an Al substrate for imaging.

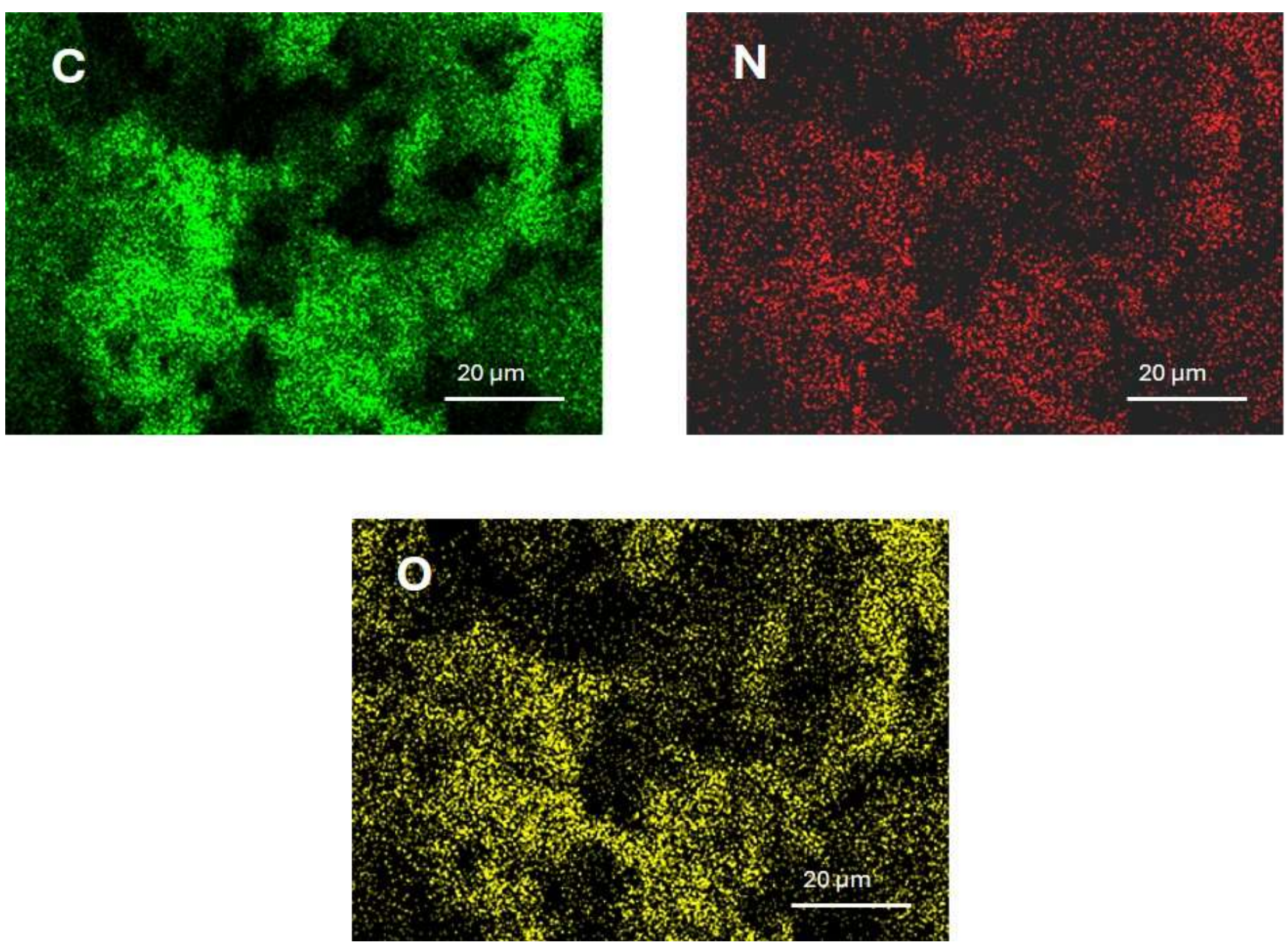

Figure S10. EDS elemental mapping of *Im-POP* showing distribution of C, N, and O.

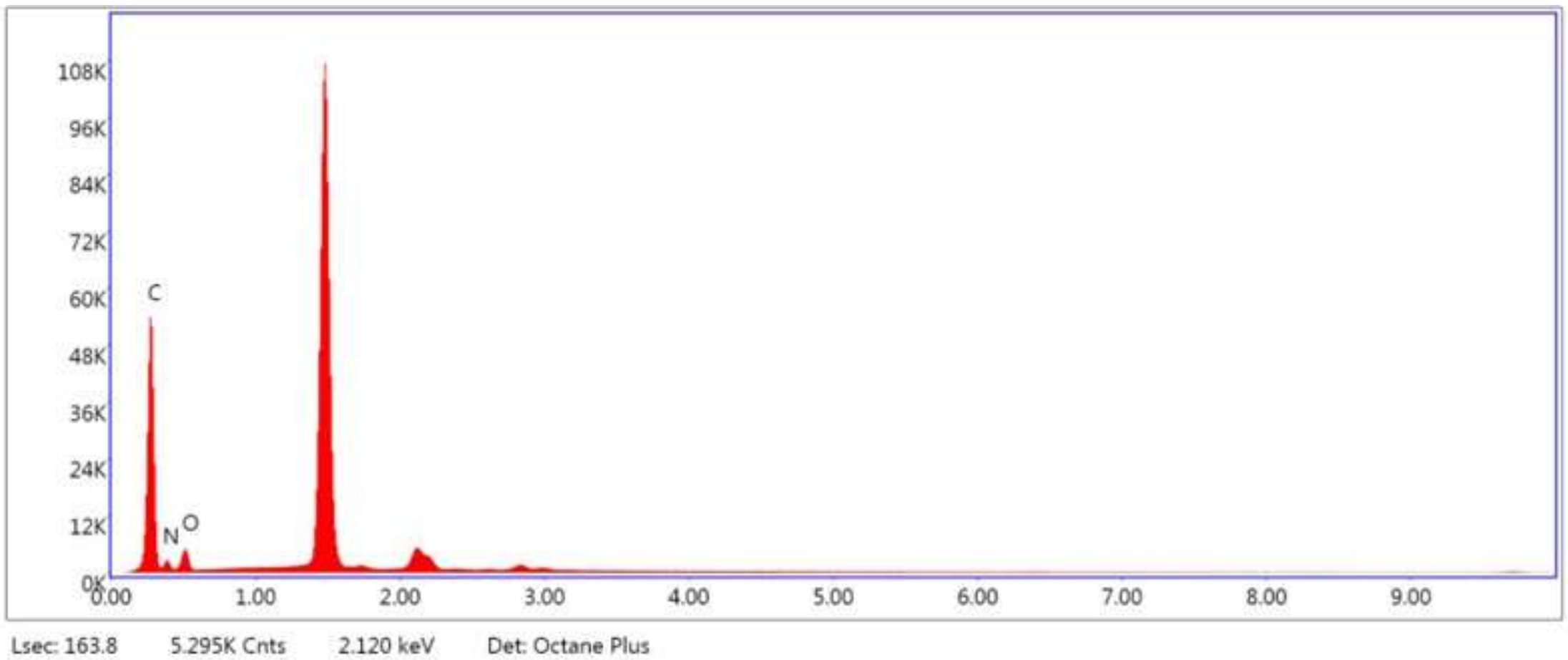

Figure S11. EDAX spectrum of *Im-POP*. The sample was drop-cast onto an Al substrate for imaging.

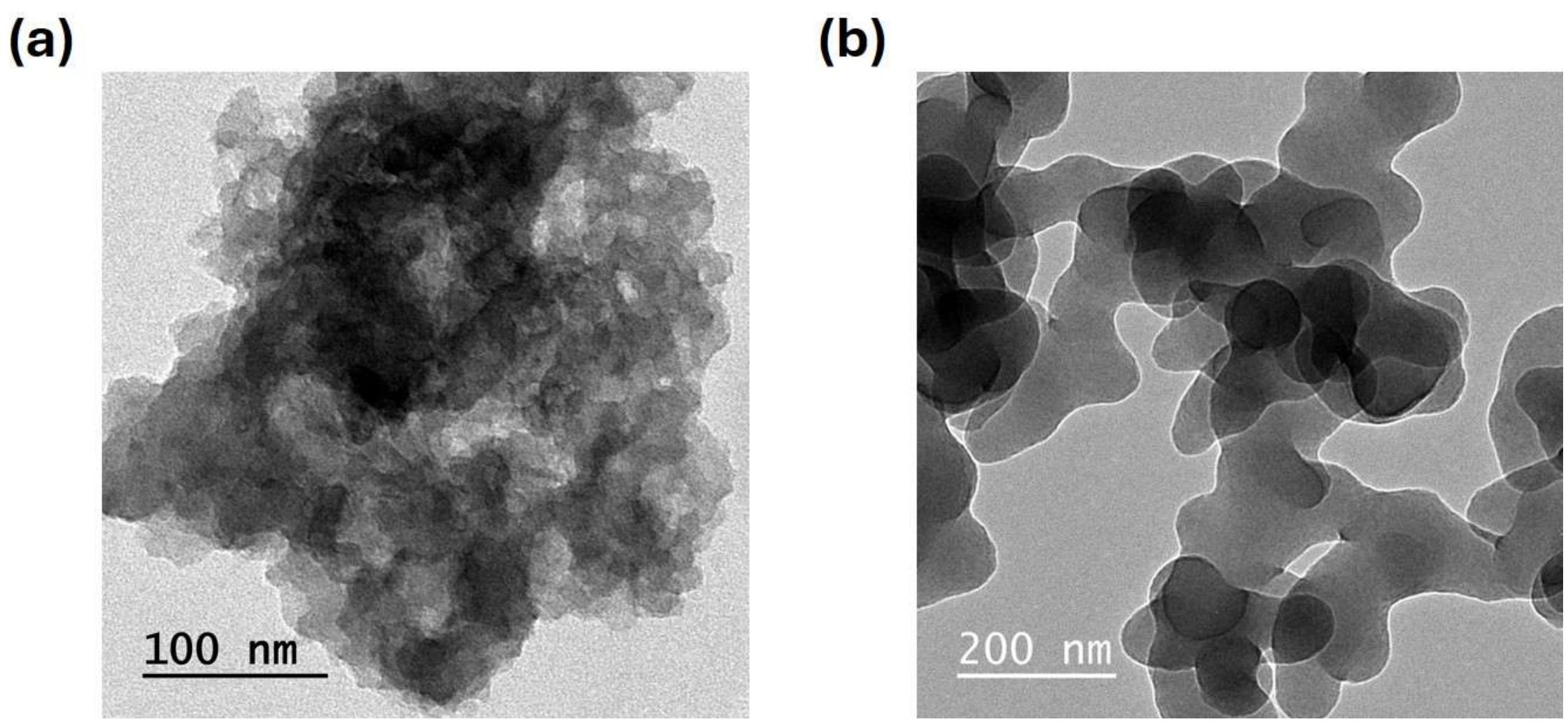

Figure S12. TEM analysis of (a) *Sal-POP* and (b) *Im-POP*.

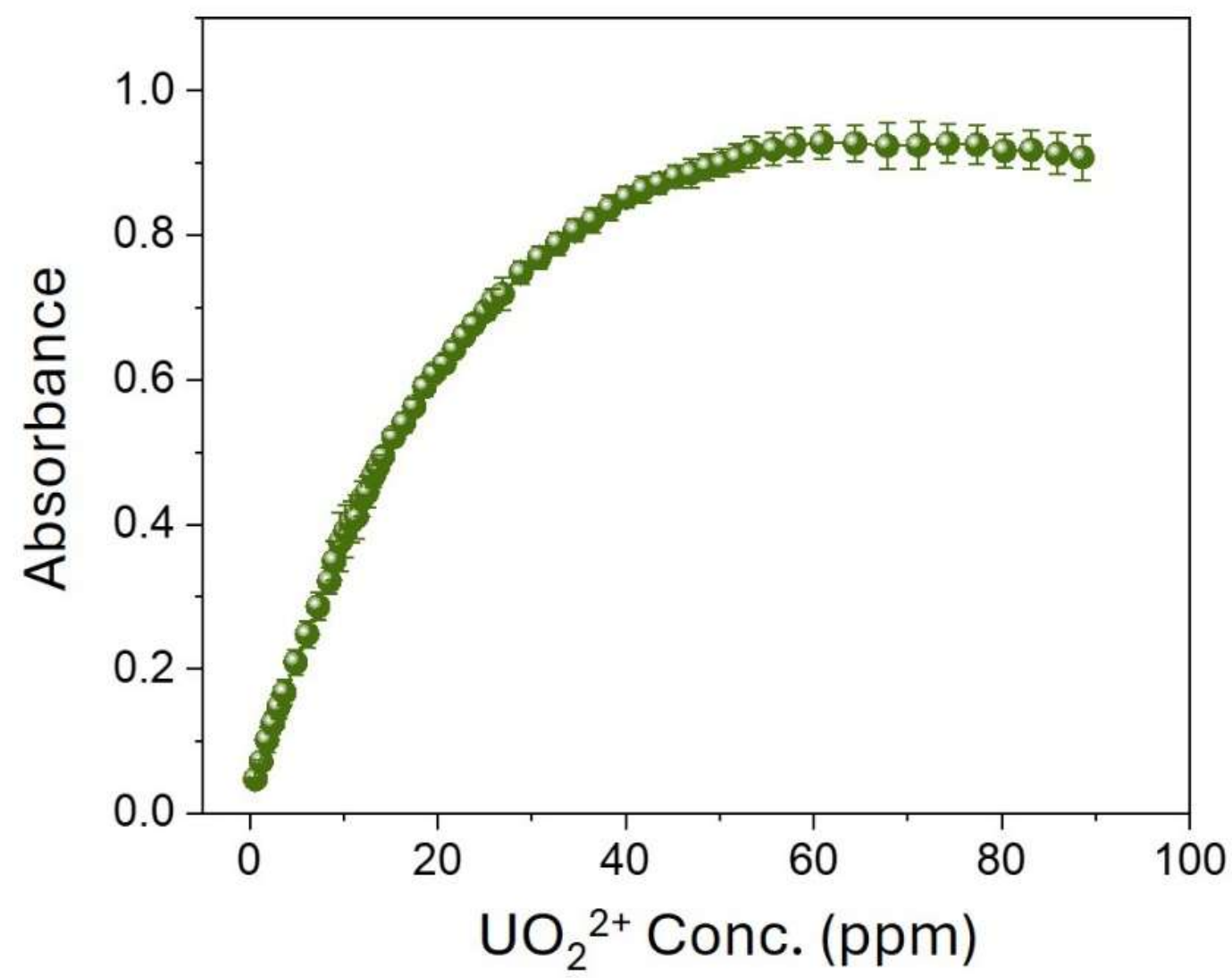

Figure S13. Calibration curve obtained from the titration of uranyl cation with Arsenazo dye III.

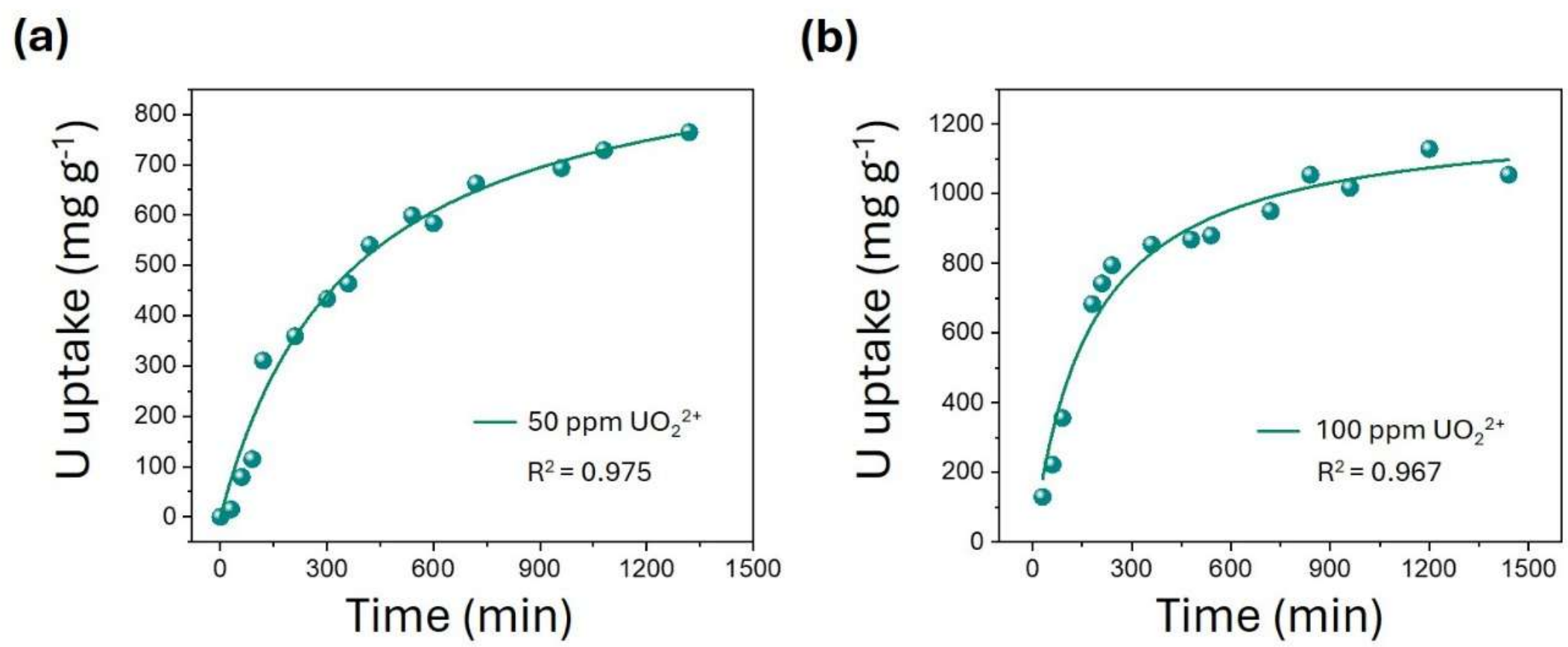

Figure S14. Uranium uptakes at a uranium concentration of (a) 50 ppm and (b) 100 ppm for *Sal-POP*.

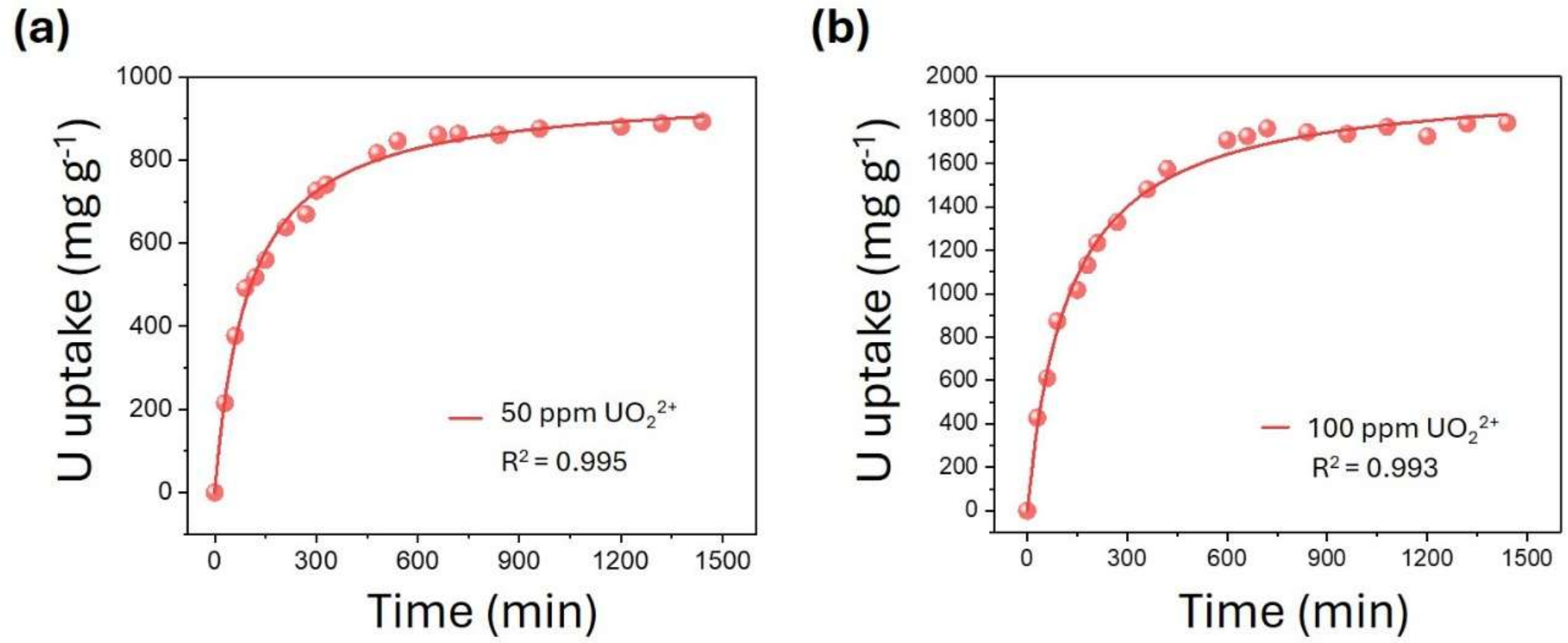

Figure S15. Uranium uptakes at a uranium concentration of (a) 50 ppm and (b) 100 ppm for *Im-POP*.

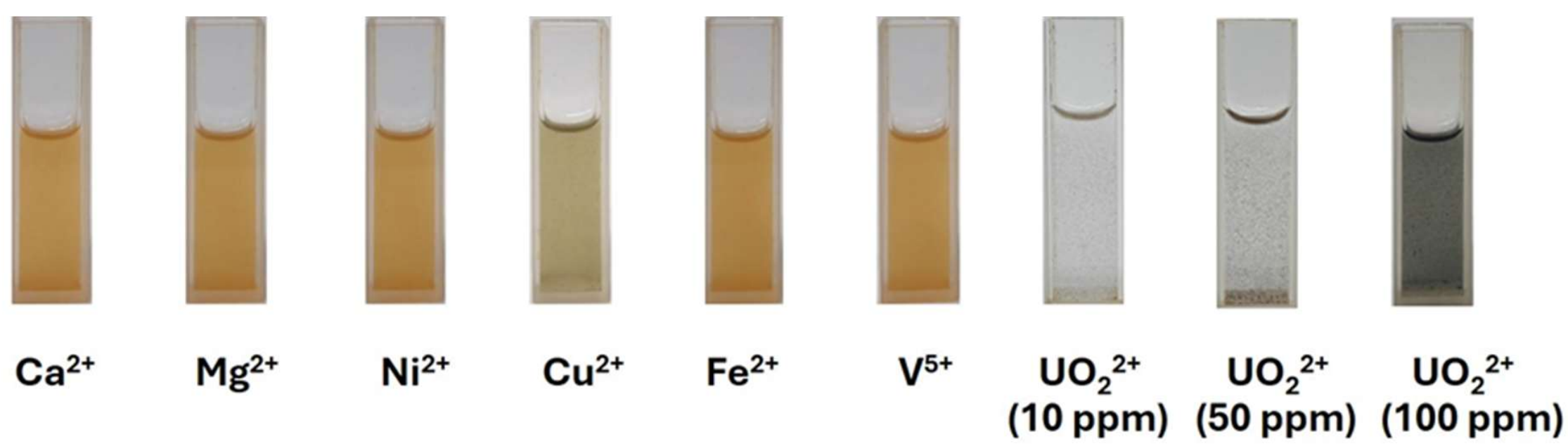

Figure S16. Daylight images of *Sal-POP* dispersions in the presence of different metal ions after 24 h.

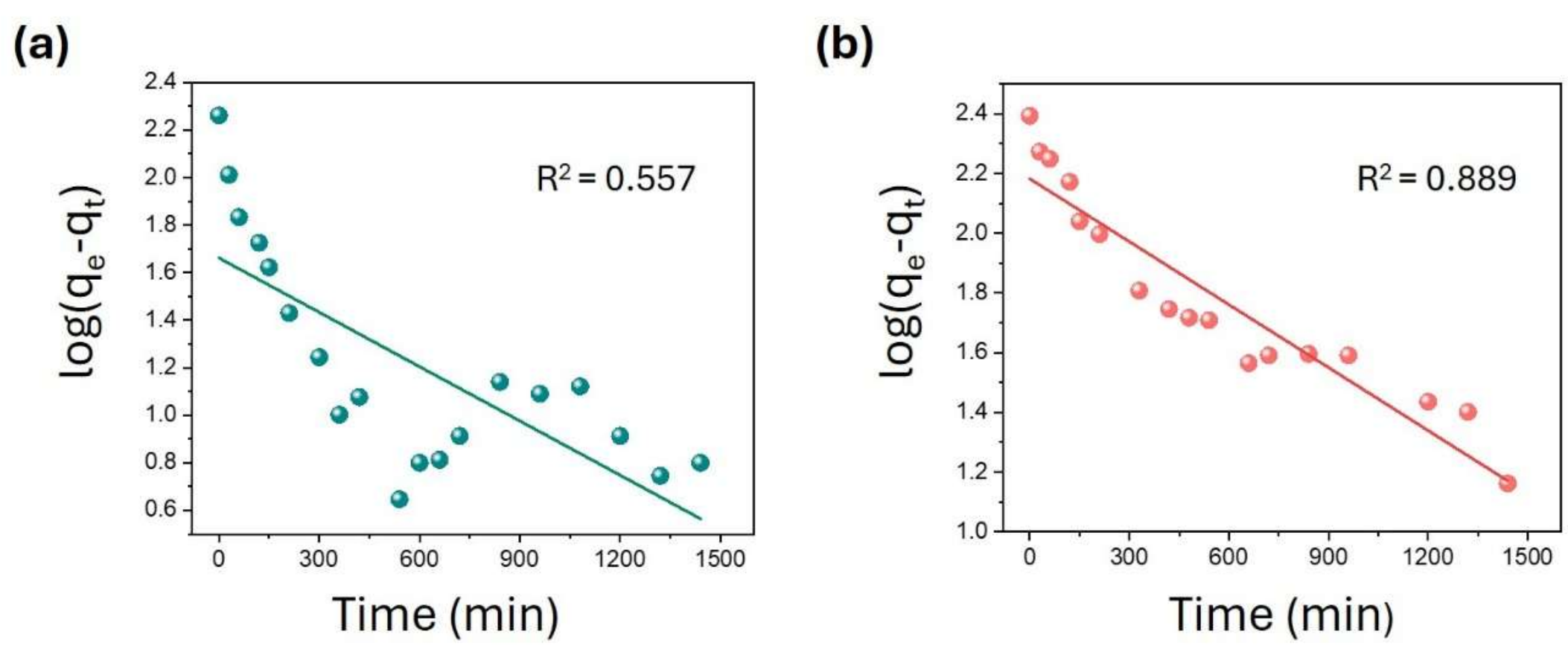

Figure S17. *Pseudo-first-order* kinetics (a) *Sal-POP* and (b) *Im-POP*.

Chemisorption of the UO_2^{2+} to the POP.

The adsorption of the UO_2^{2+} on the POP occurs via the chemisorption where the UO_2^{2+} coordinates with the *o*-hydroxy imine ligand units present on the POP surface. DFT analysis indicated seven coordination number for uranyl and pentagonal bipyramidal coordination geometry with water solvent molecules fulfilling the other coordination sites. The coordination environment around the UO_2^{2+} is shown below. This was also supported by the FT IR spectroscopy.

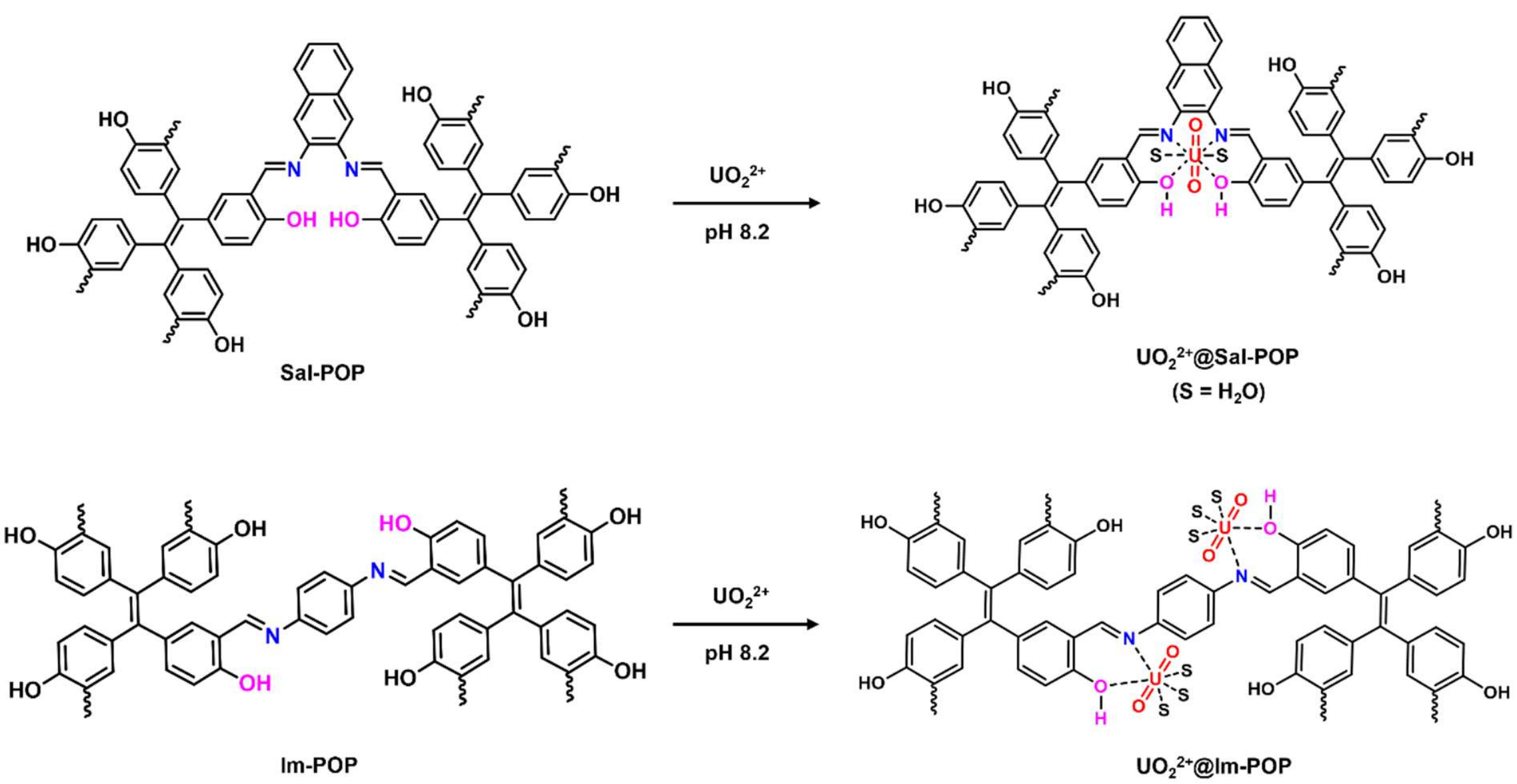

Figure S18. Chemical structures showing the coordination of uranyl cation to the *Sal-POP* (a) and *Im-POP* (b) under simulated seawater conditions. Here the coordinated solvent (S) is water molecules.

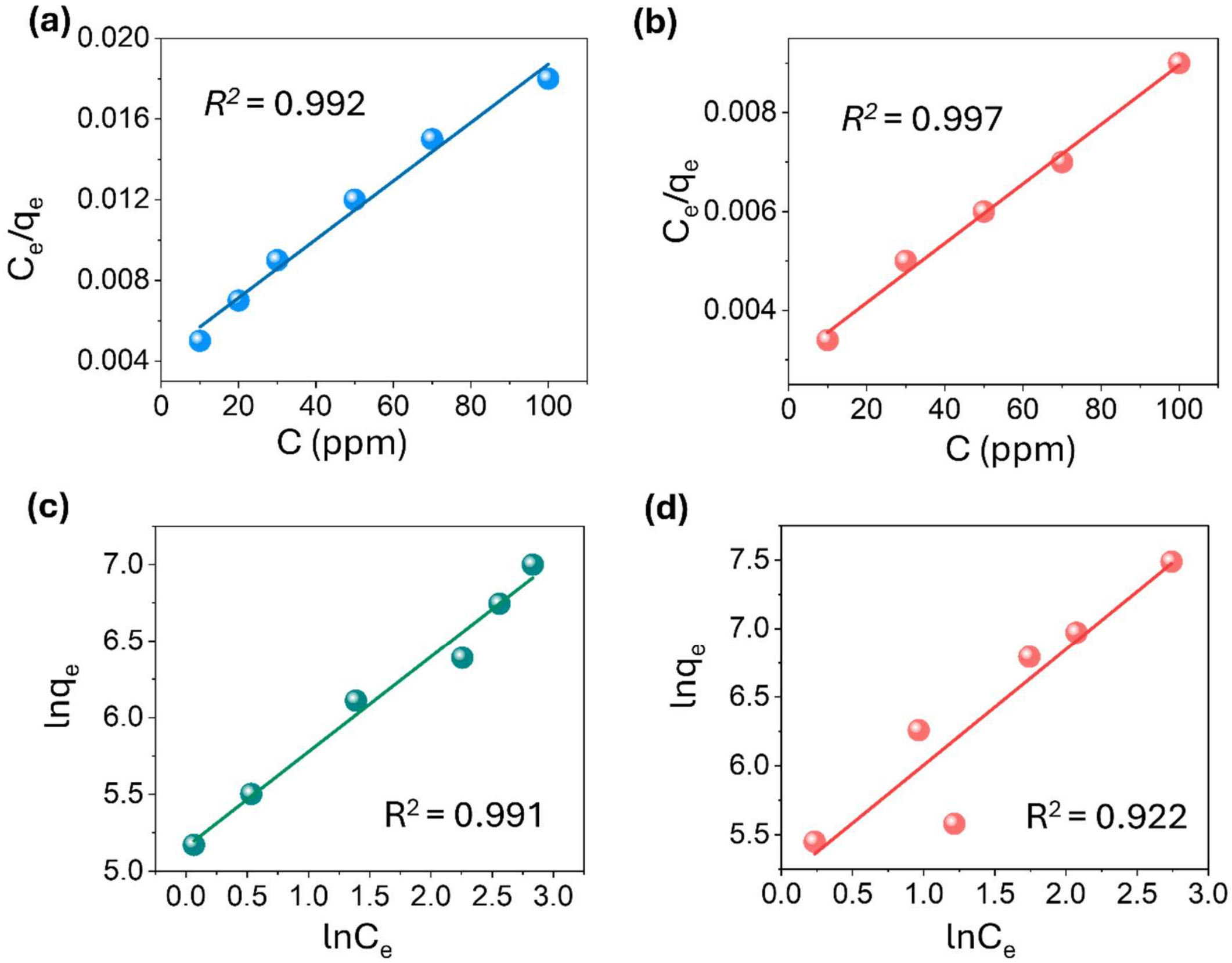

Figure S19. Top: Linear fitting of uranyl adsorption process to *Langmuir* adsorption isotherm model for (a) *Sal-POP* and (b) *Im-POP*. Bottom: *Freundlich* adsorption isotherms of (c) *Sal-POP* and (d) *Im-POP*.

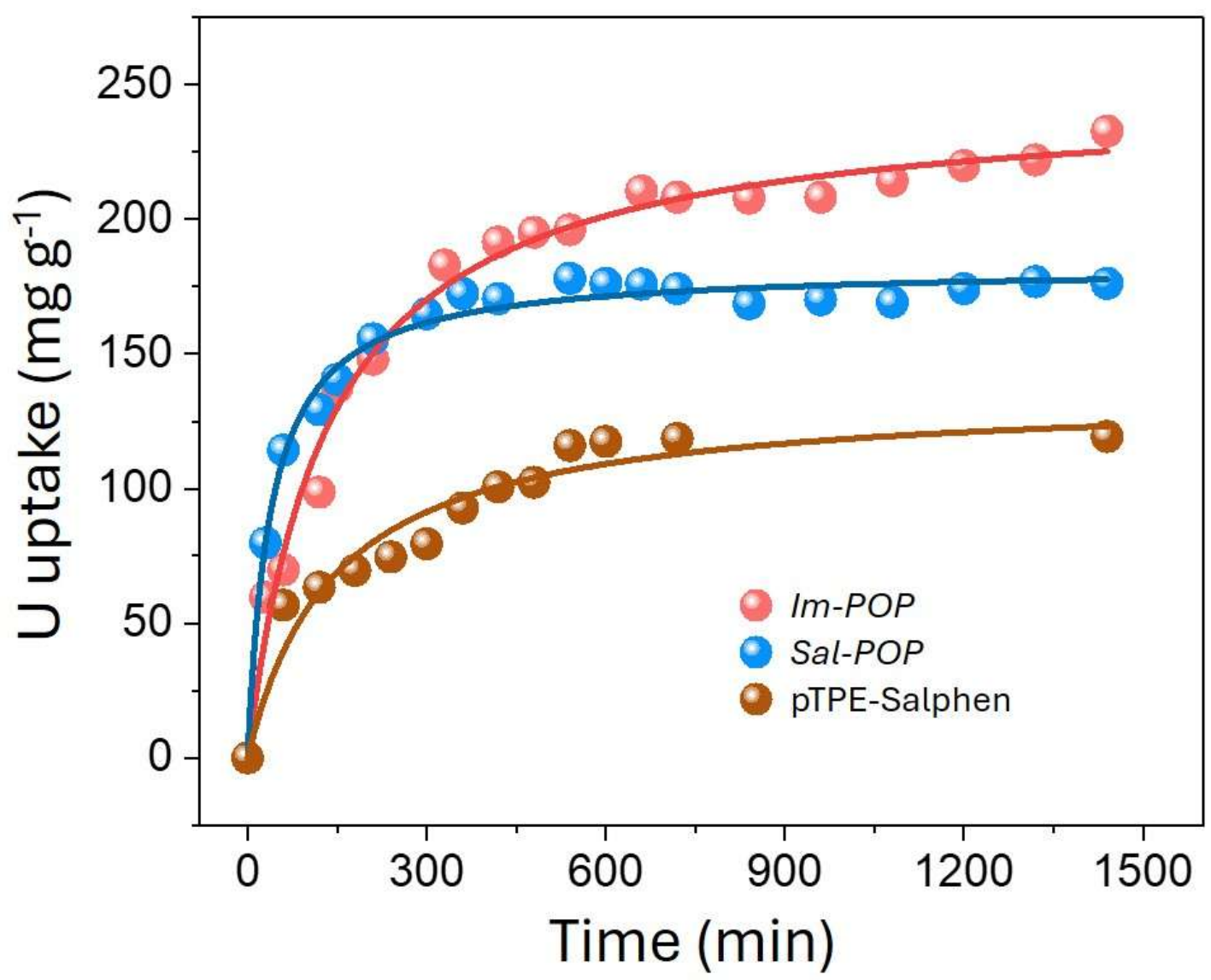

Figure S20. Time-dependent uranium uptake performance of *Im-POP* (red), *Sal-POP* (blue), and previously reported pTPE-Salphen[1] (brown) under simulated seawater conditions. Measured with a 10 ppm uranyl solution in simulated seawater.

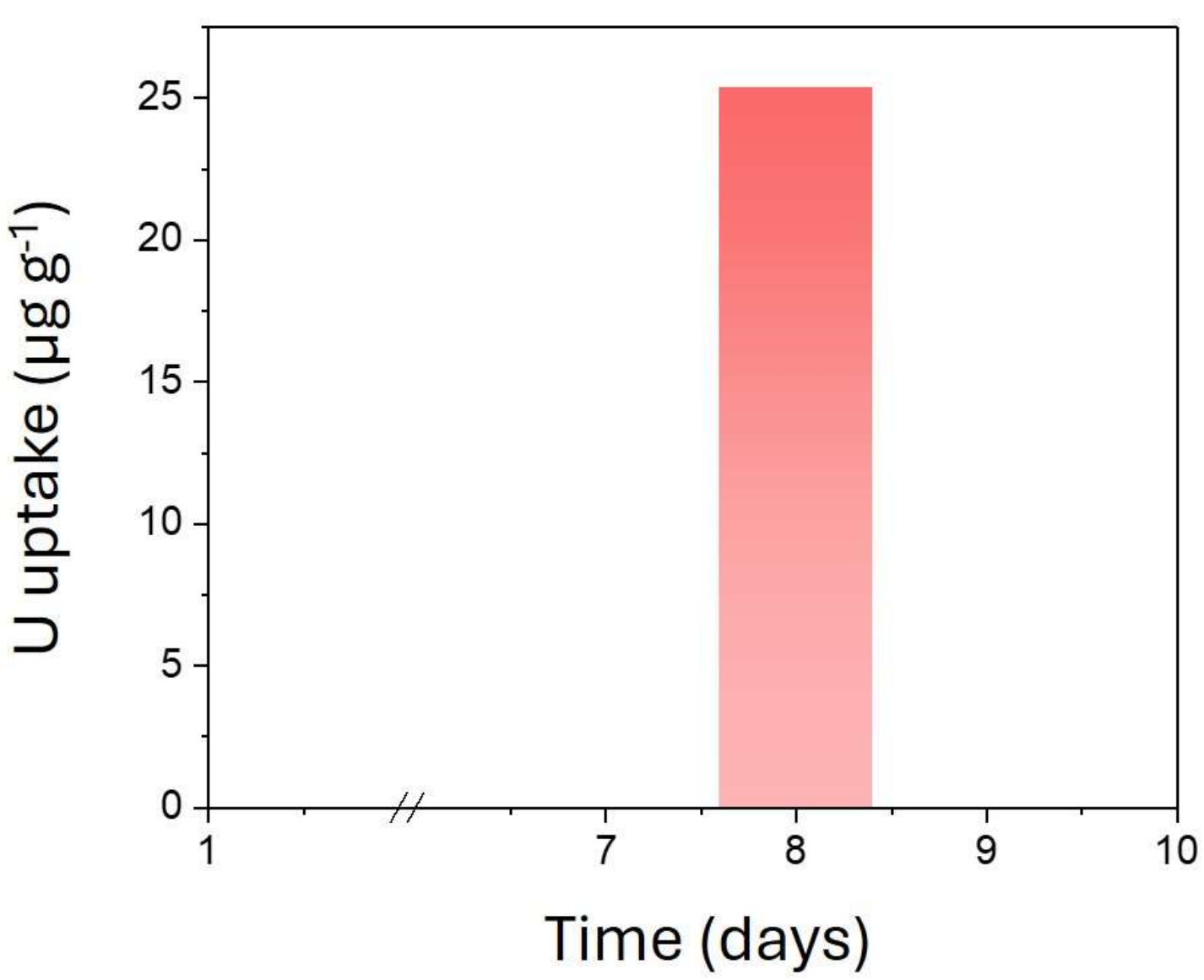

Figure S21. Uranium uptake performance of *Im-POP* from natural seawater.

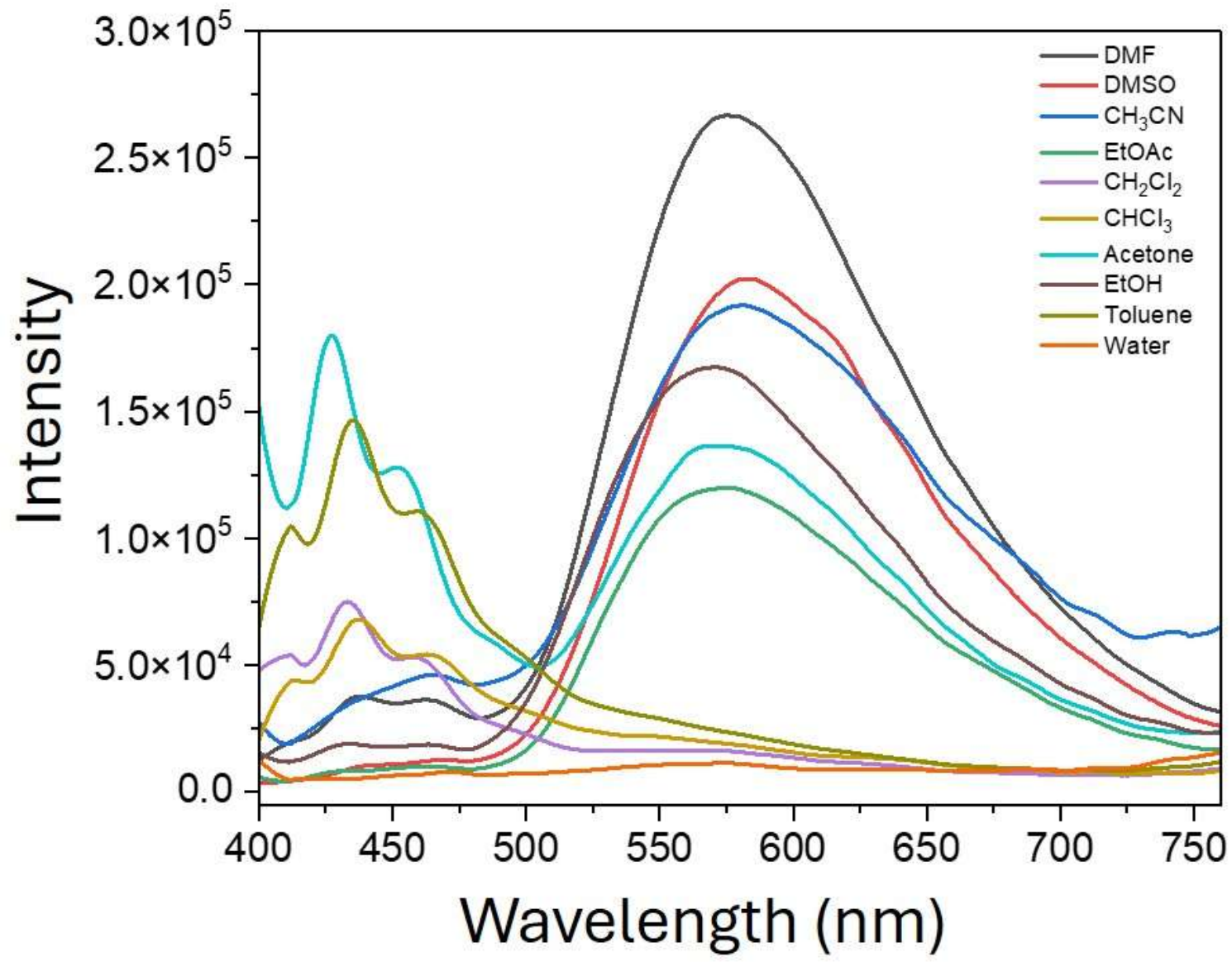

Figure S22. Photoluminescence emission spectra of *Sal-POP* (0.5 mg mL^{-1}) in different solvents.

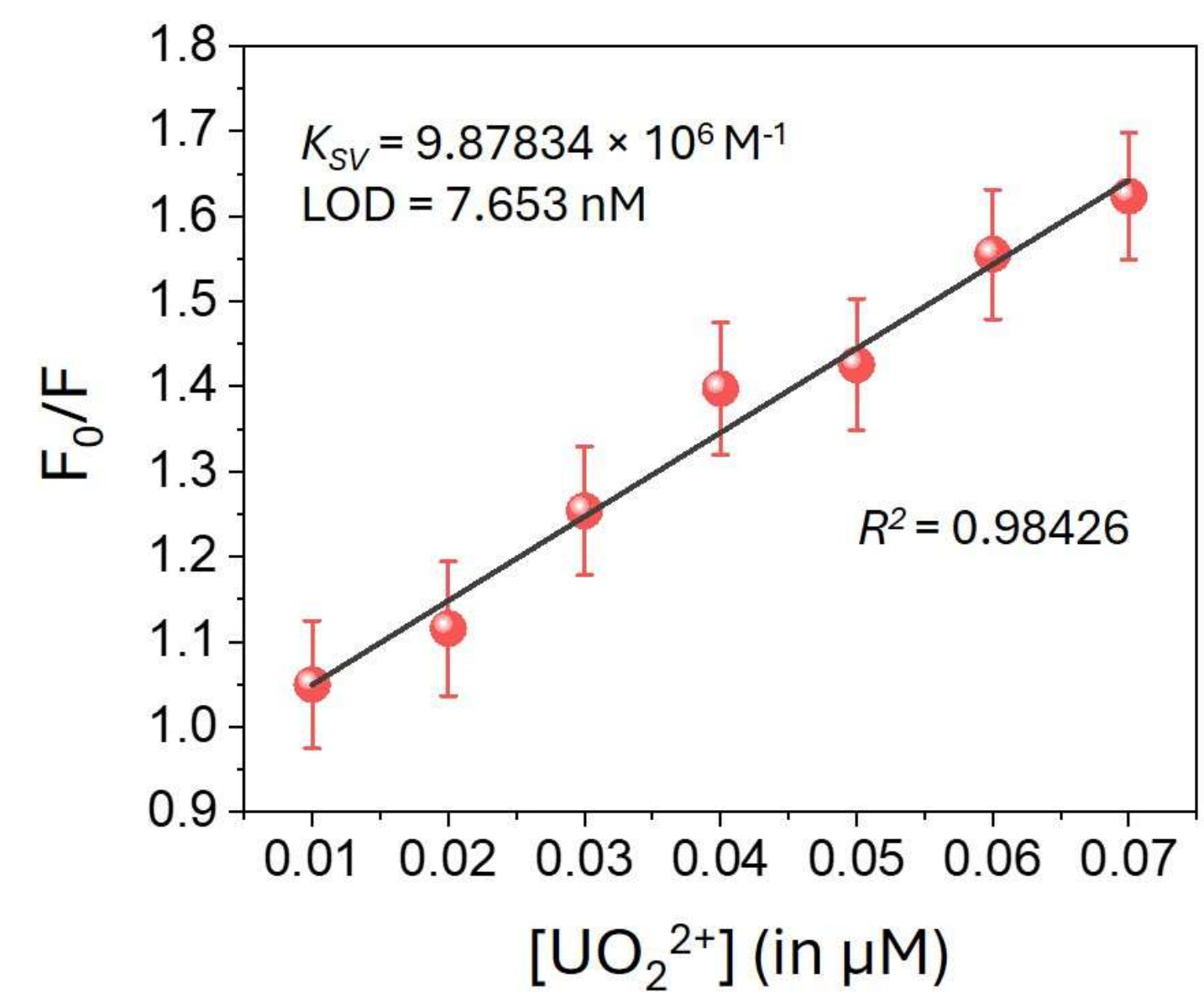

Figure S23. Linear fitting of Stern-Volmer plot, F_0/F vs [UO_2^{2+}] at low concentrations.

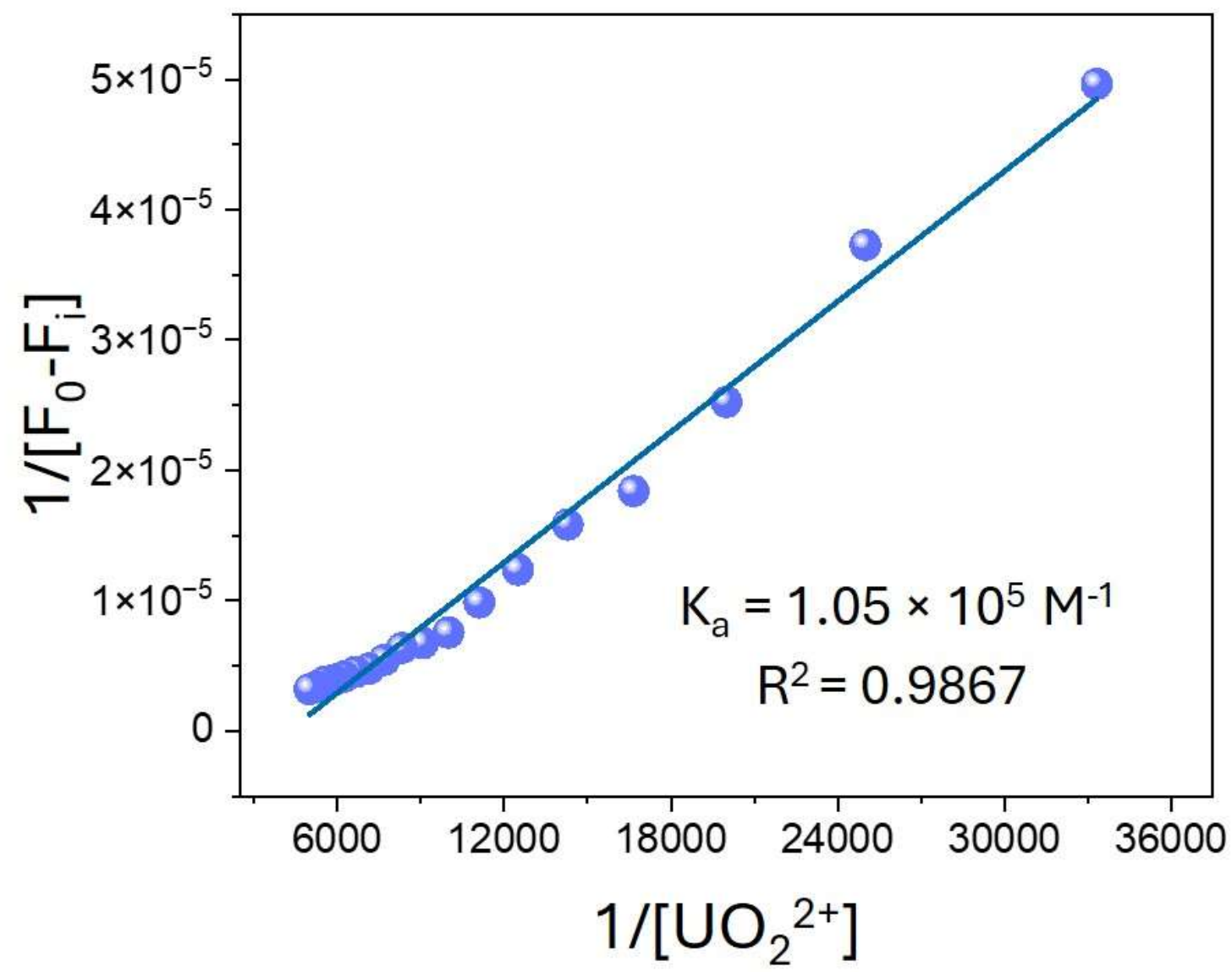

Figure S24. Benesi–Hildebrand plots for *Sal-POP* upon addition of UO_2^{2+}.

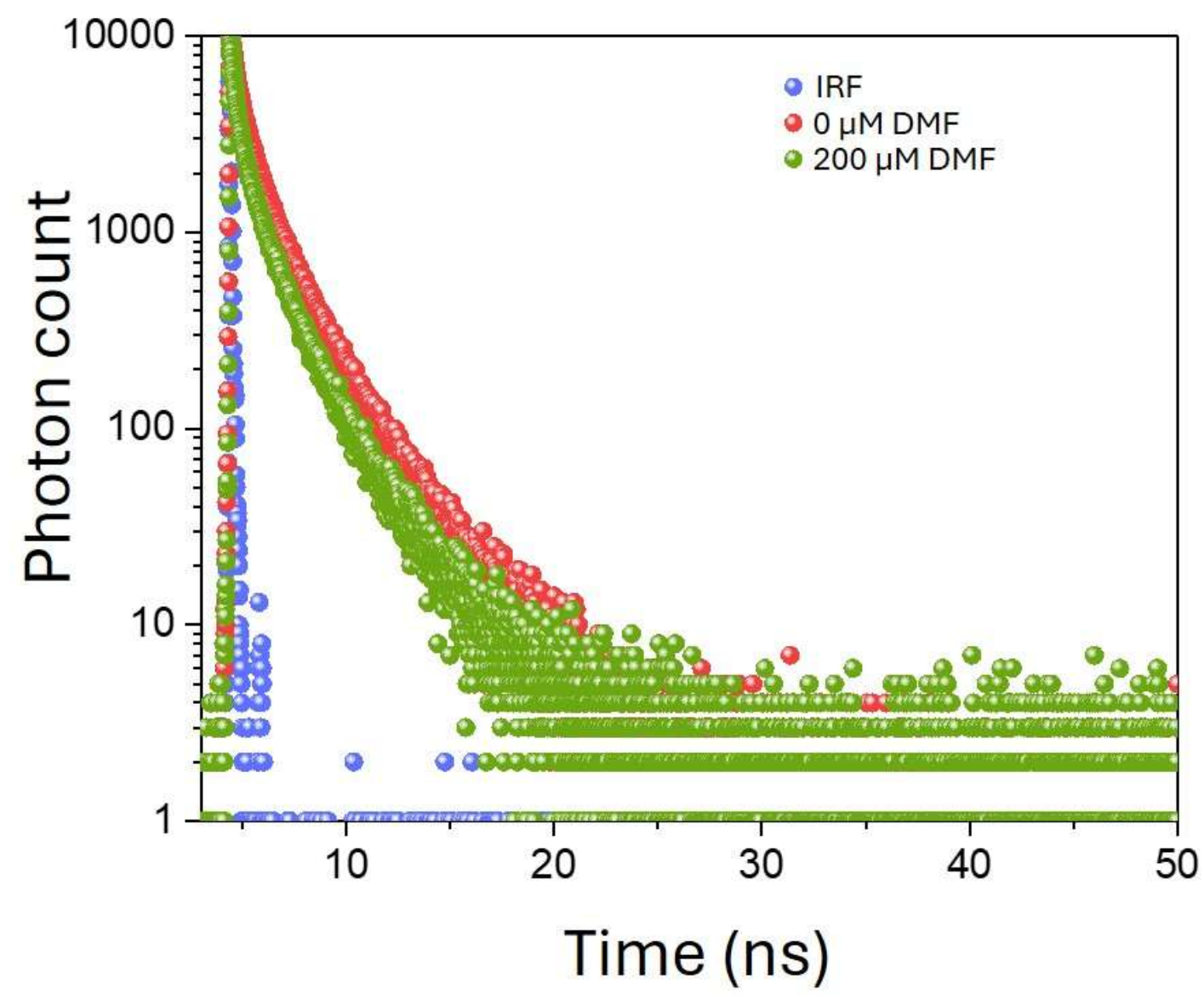

Figure S25. Fluorescence excited-state lifetime decay profiles of *Sal-POP* (red) and UO_2^{2+}@*Sal-POP* (green) in DMF dispersion. The instrument response factor (IRF) is shown in blue.

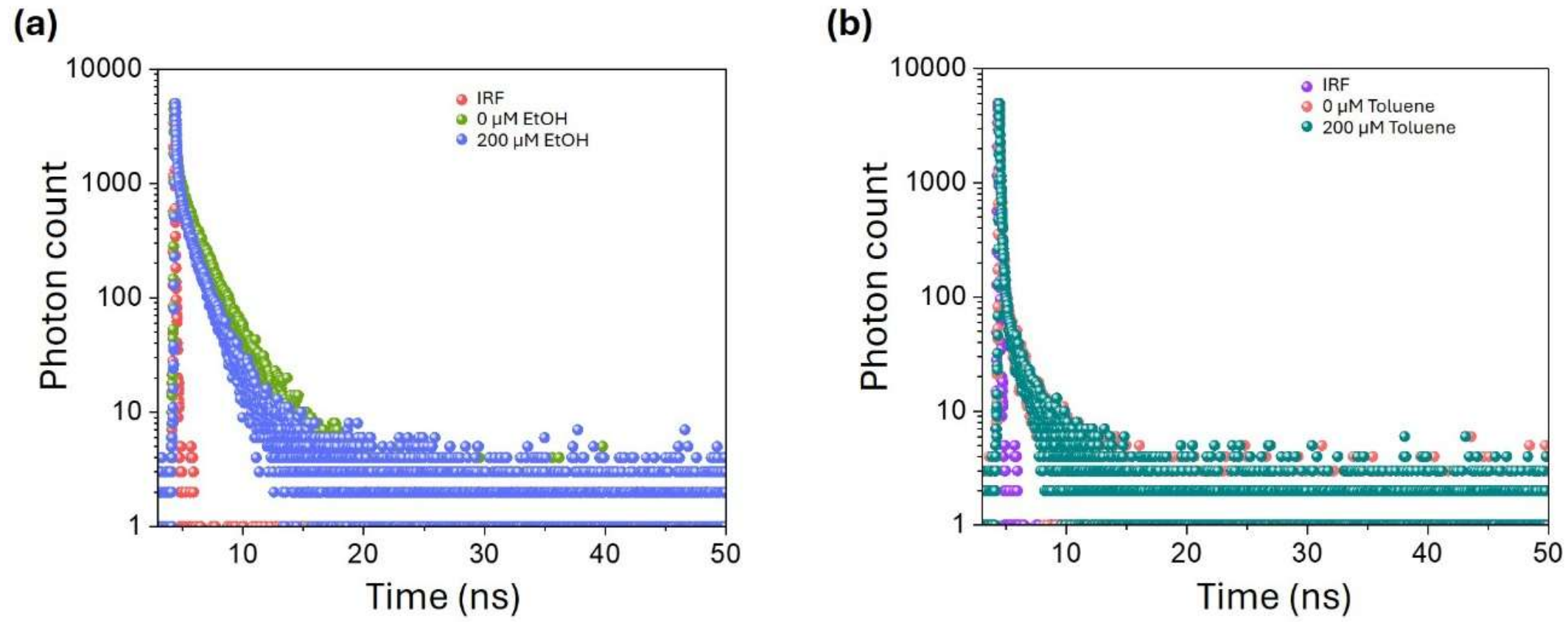

Figure S26. Fluorescence excited-state lifetime decay profiles of *Sal-POP* and UO_2^{2+}@*Sal-POP* in different solvents, (a) EtOH and (b)Toluene.

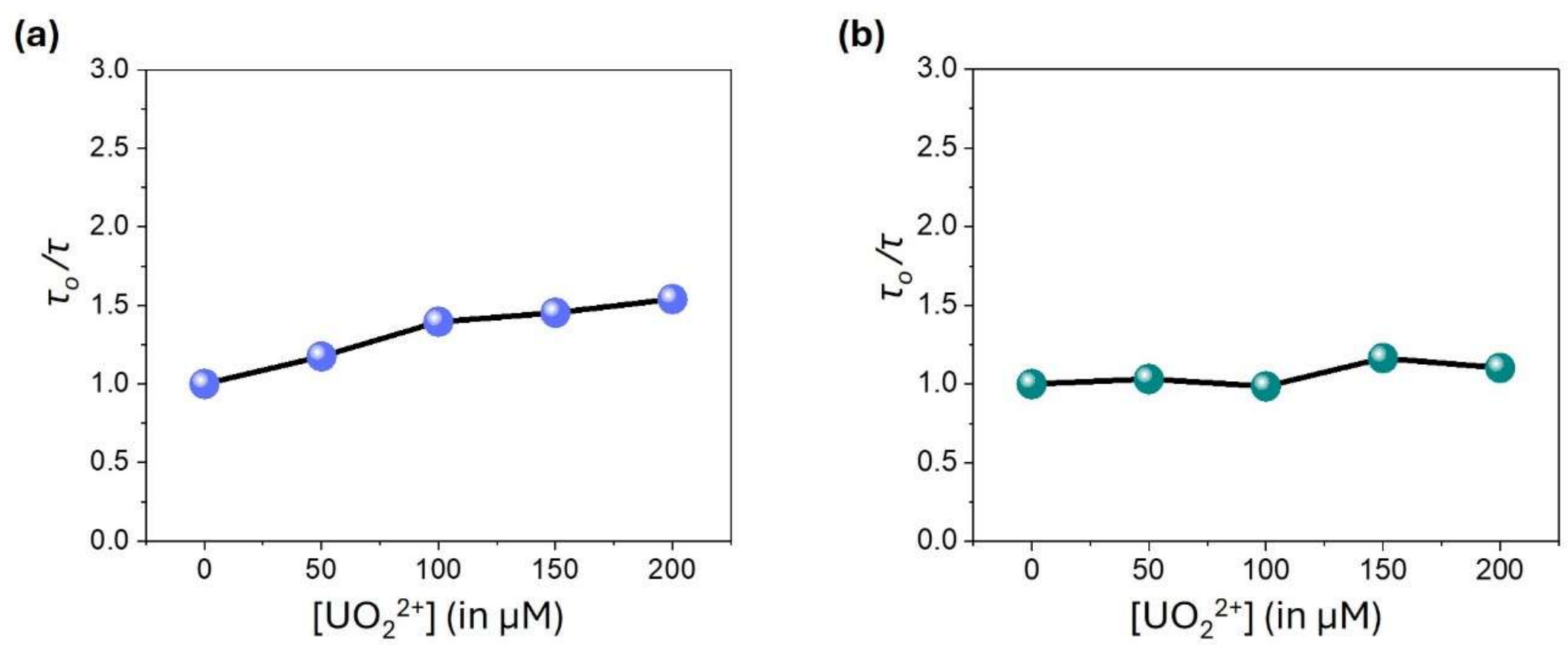

Figure S27. Excited-state lifetime changes of *Sal-POP* with increasing UO_2^{2+} concentration (0–200 μM) in (a) EtOH and (b) toluene.

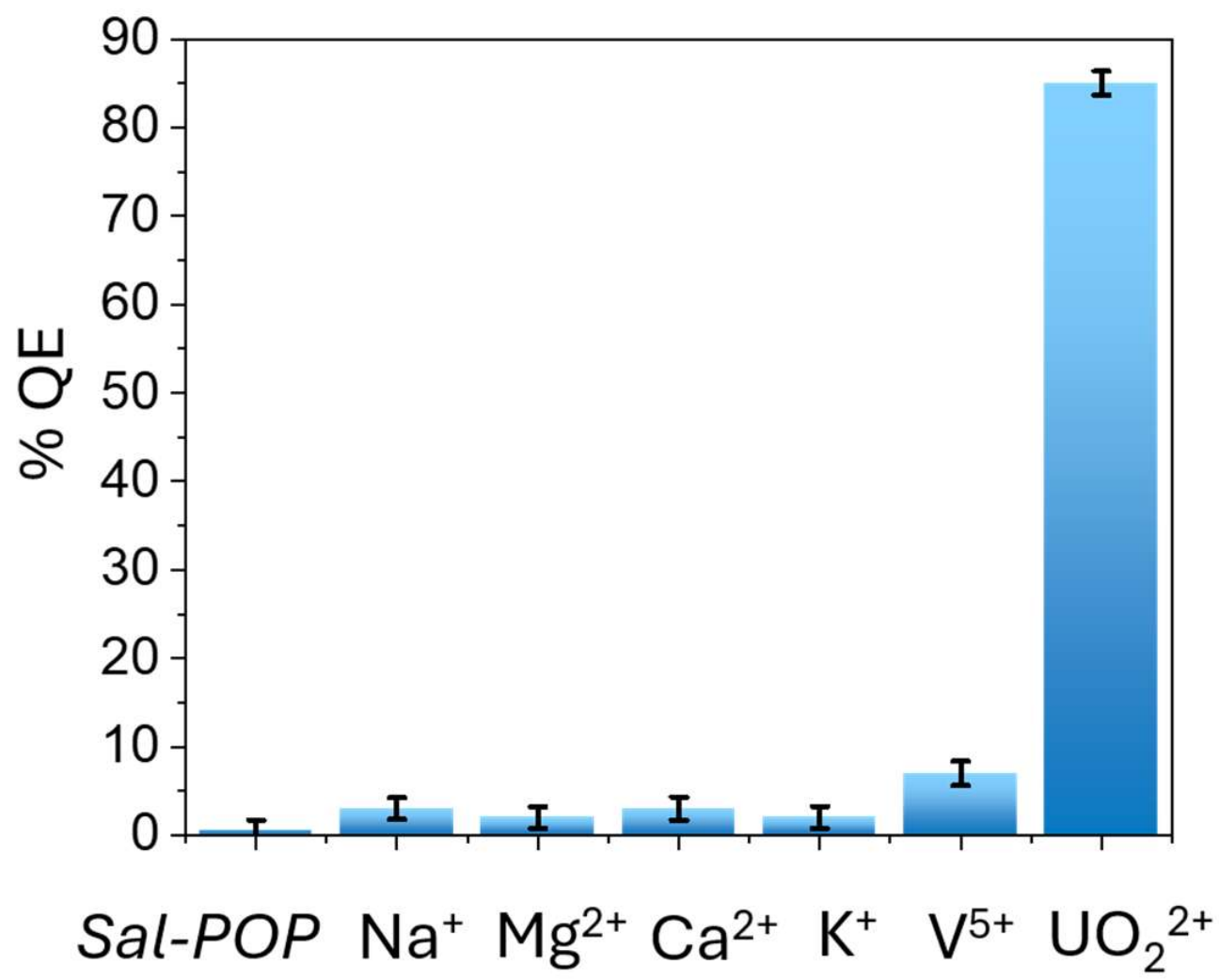

Figure S28. Comparison of the fluorescence quenching responses *of Sal-POP* toward UO_2^{2+} and competing metal ions commonly present in seawater.

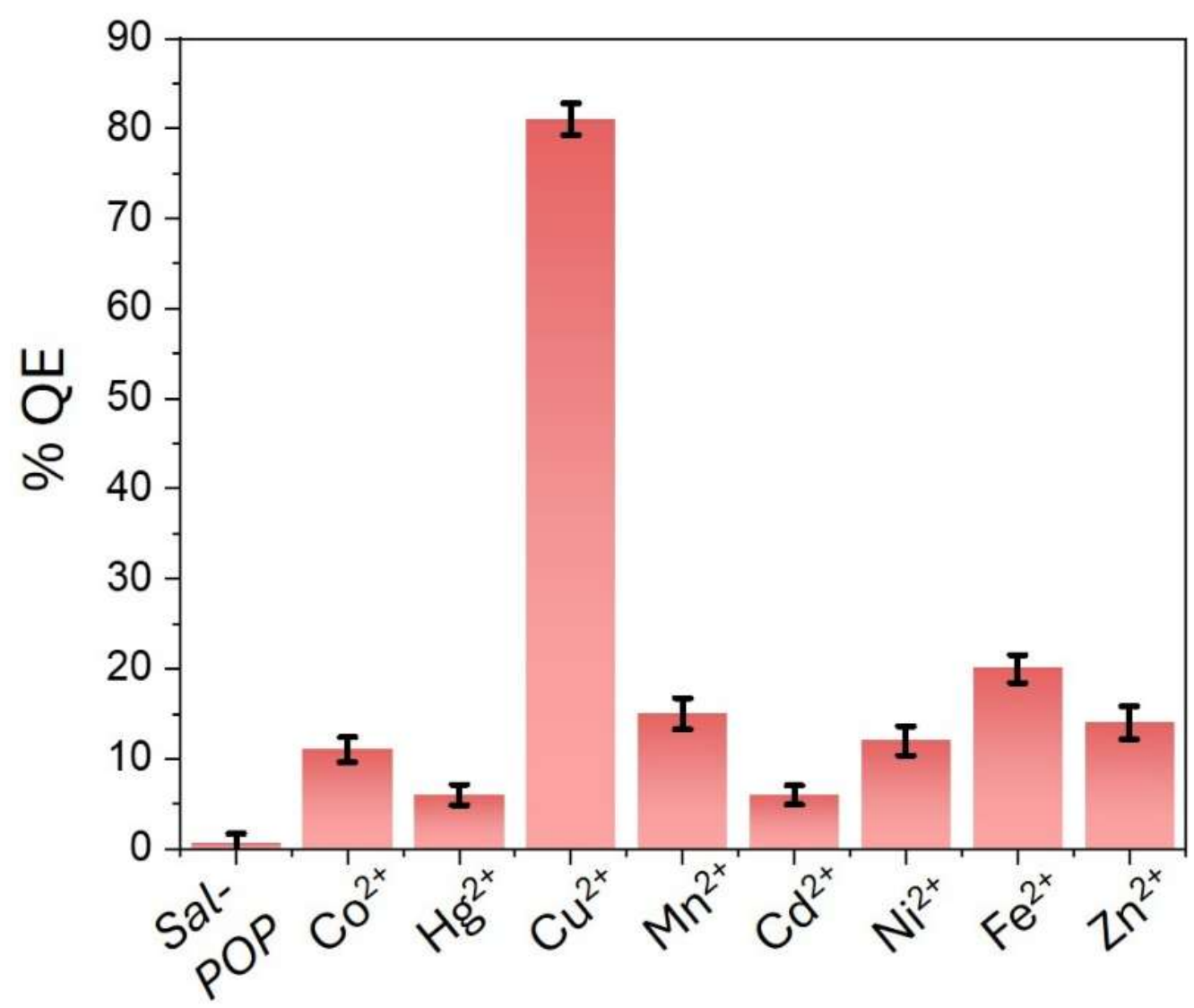

Figure S29. Comparison of fluorescence quenching efficiencies for *Sal-POP* in the presence of other metal ions.

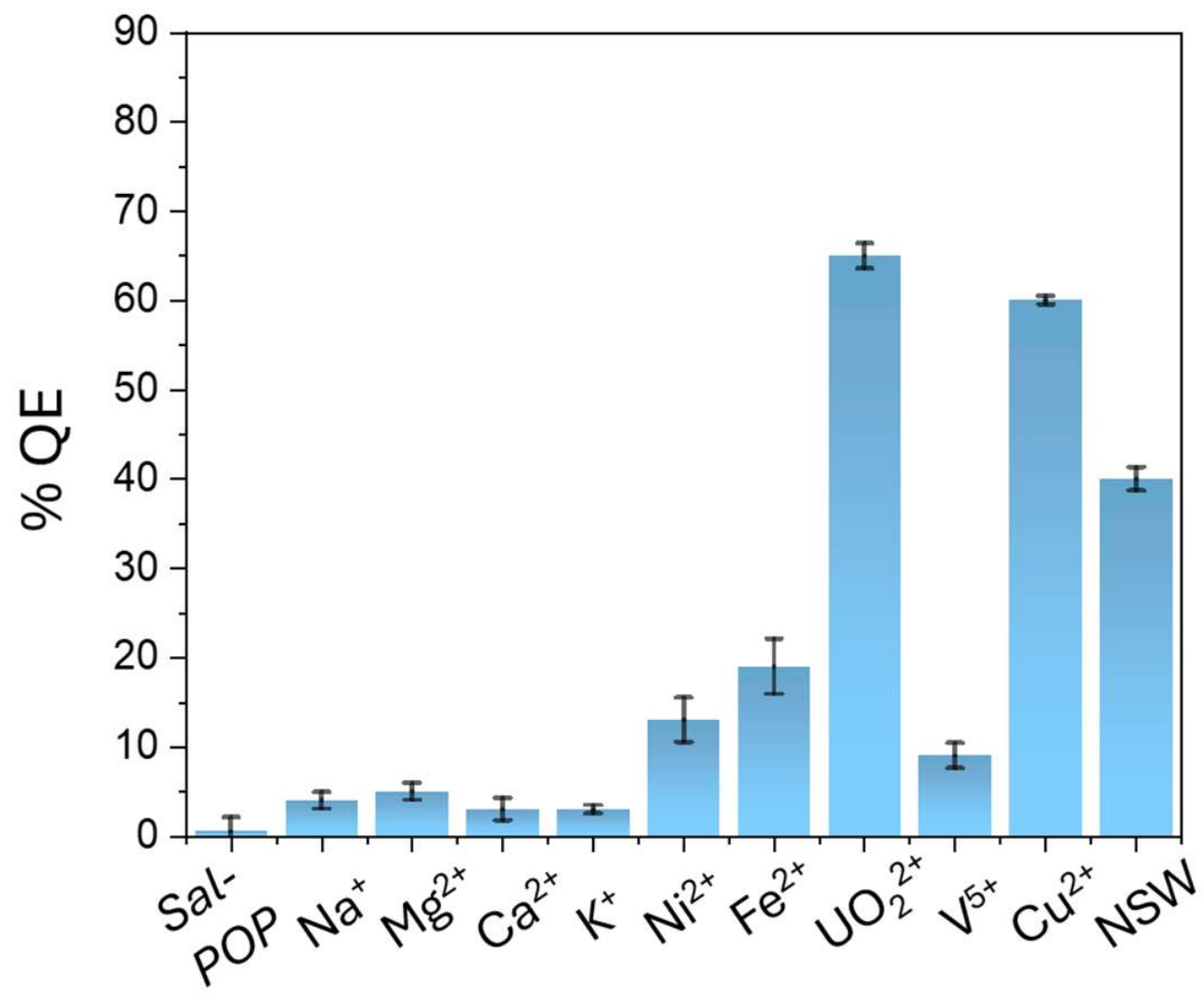

Figure S30. Comparison of the fluorescence quenching efficiency for *Sal-POP* in presence of various metal ions (50 ppm) in simulated seawater and natural seawater (NSW).

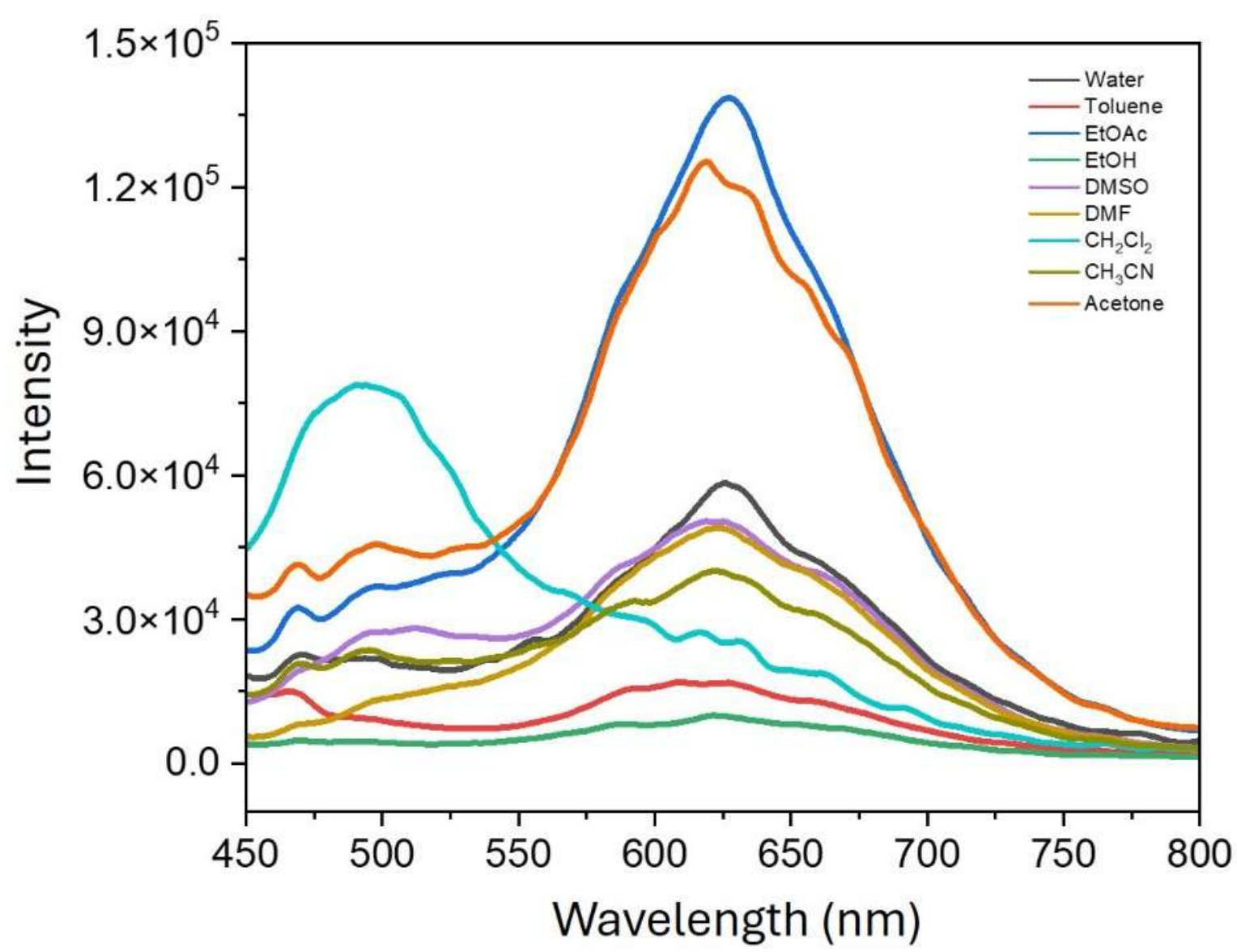

Figure S31. Photoluminescence spectra of *Im-POP* (0.5 mg mL^{-1}) in different solvents.

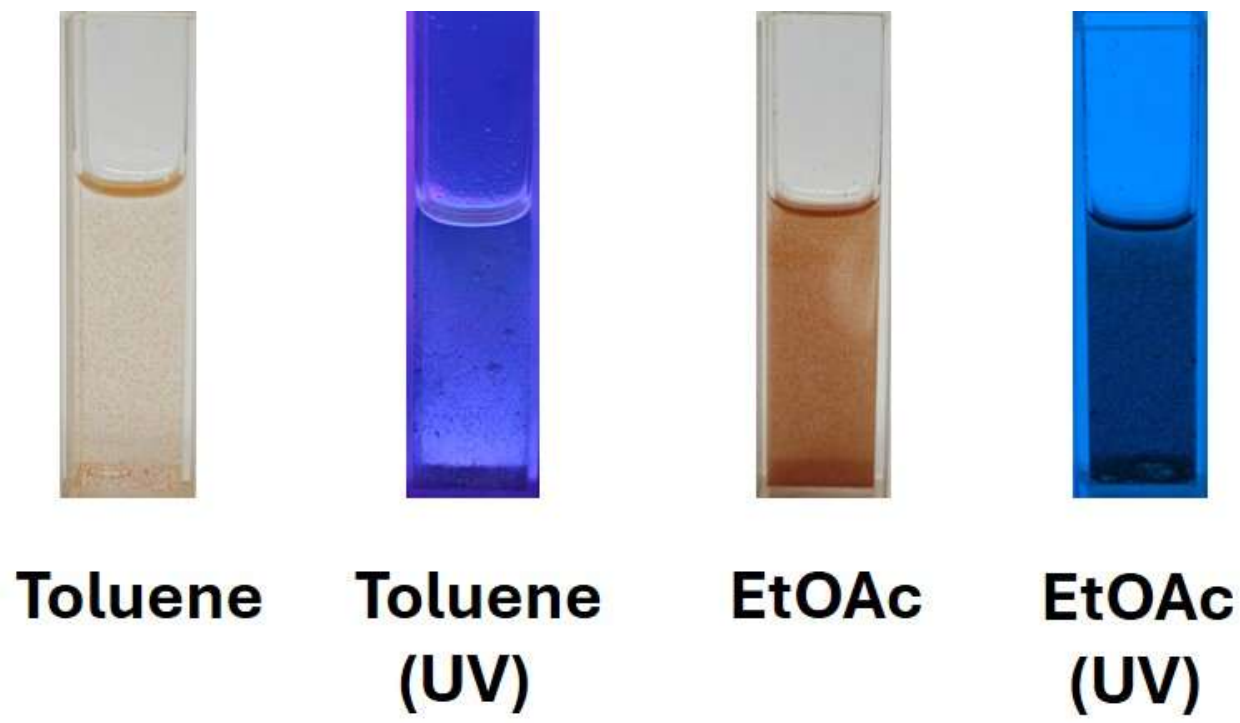

Figure S32. Image of *Im-POP* dispersions under daylight and UV light (365 nm) in different solvents.

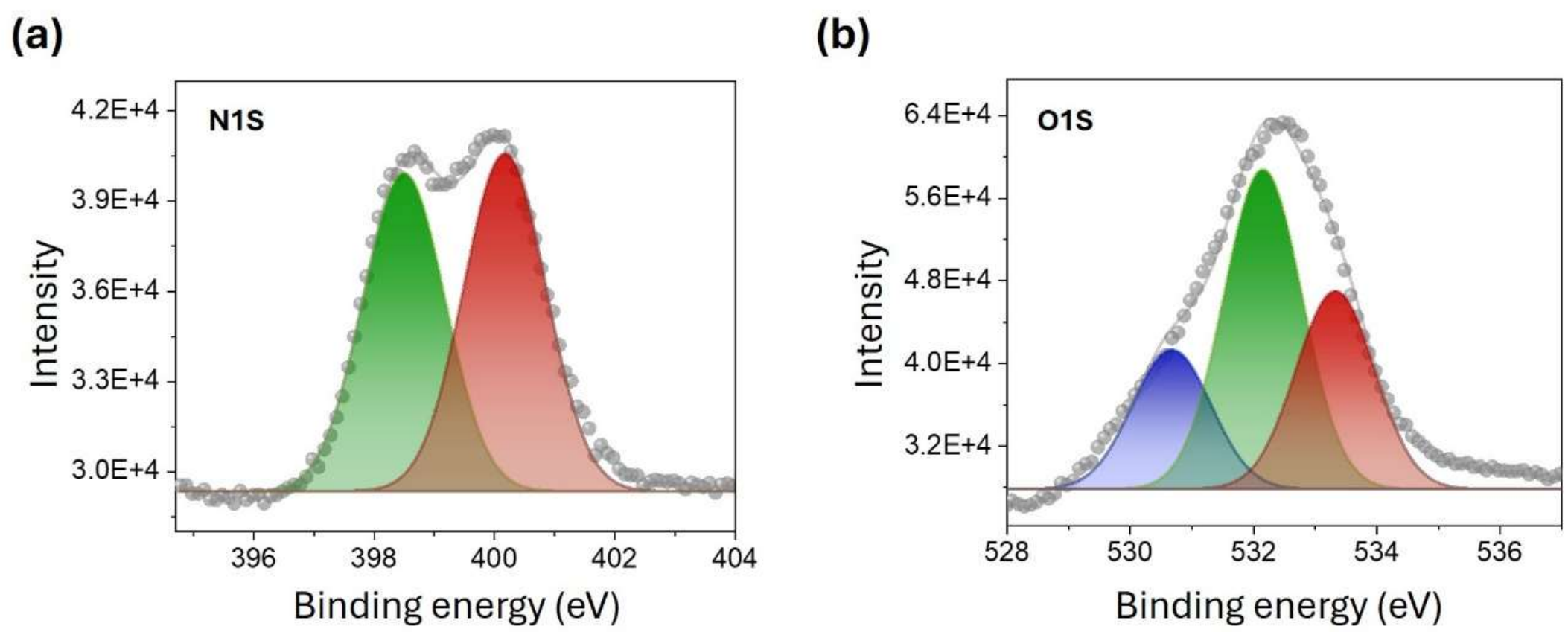

Figure S33. Deconvoluted XPS spectra of (a) N1s, and (b) O1s of UO_2^{2+}@*Sal-POP*.

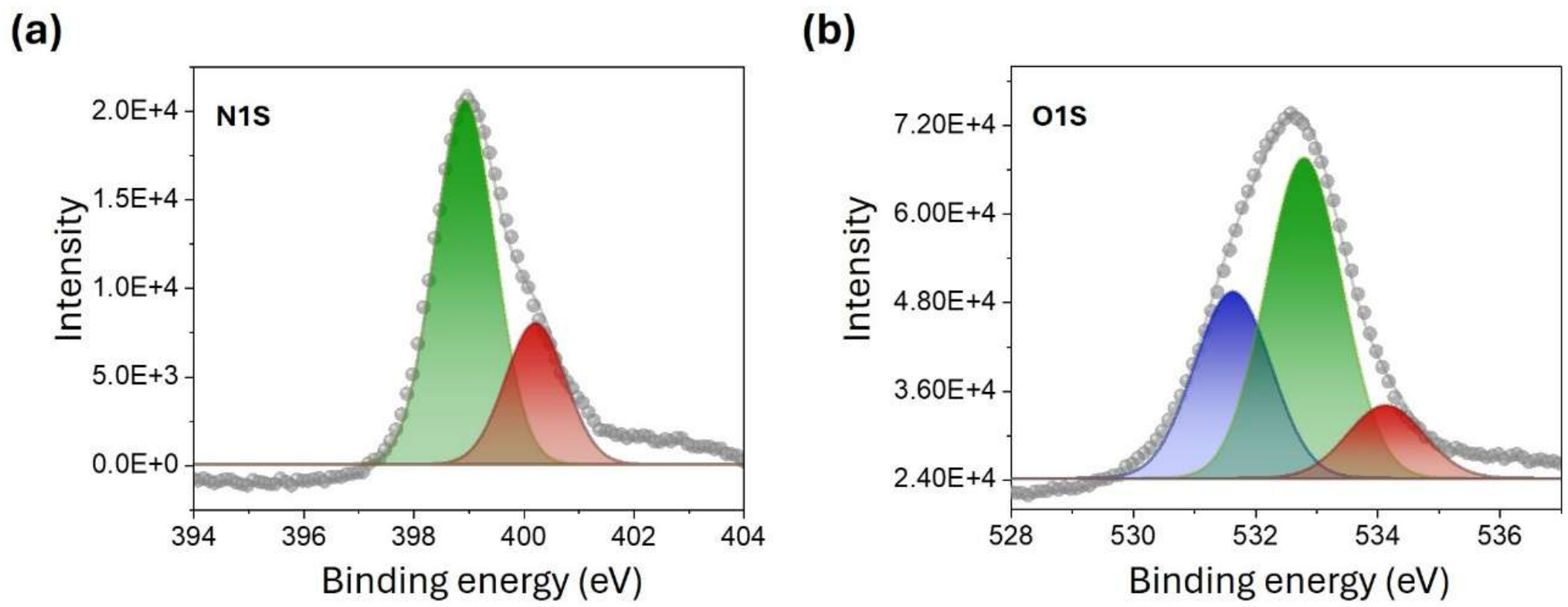

Figure S34. Deconvoluted XPS spectra of (a) N1s, and (b) O1s of UO_2^{2+}@*Im-POP*.

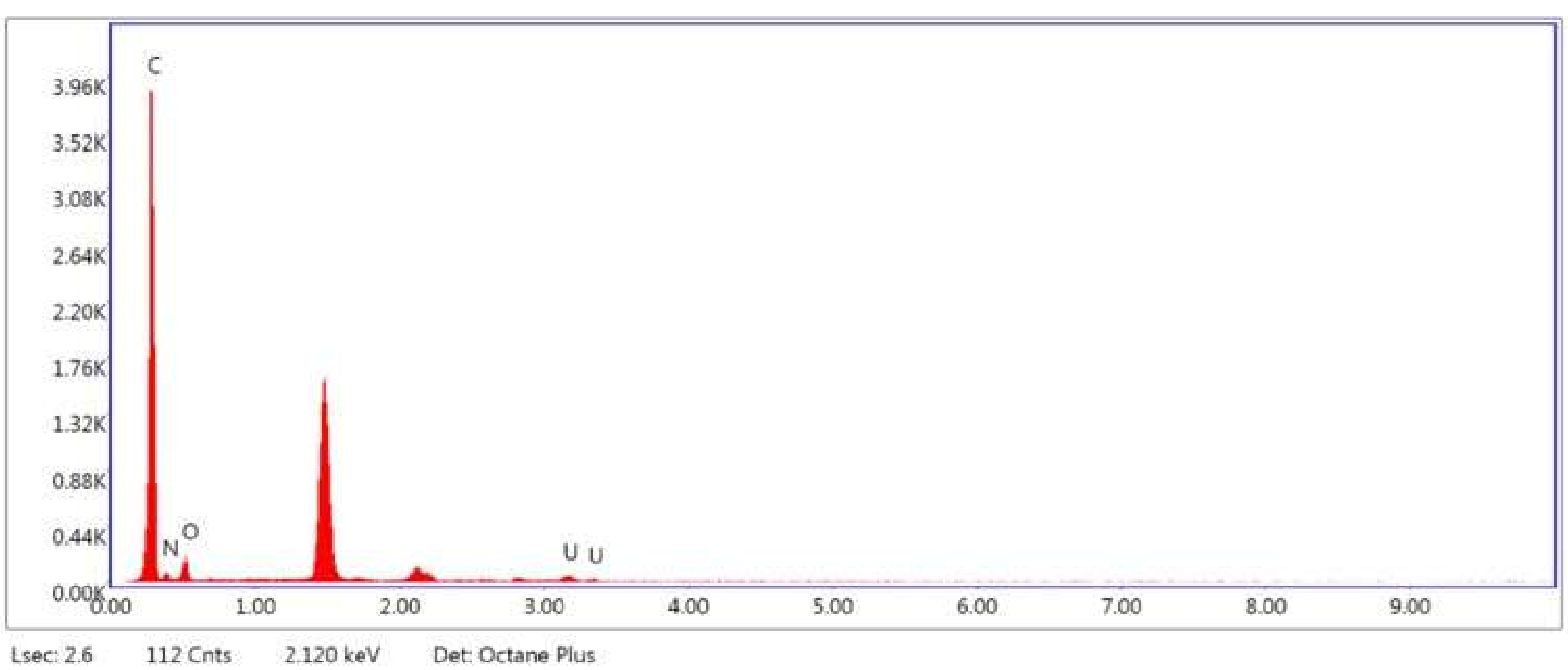

Figure S35. EDAX spectrum of UO_2^{2+}@*Sal-POP*. The sample was drop-cast onto an Al substrate for imaging.

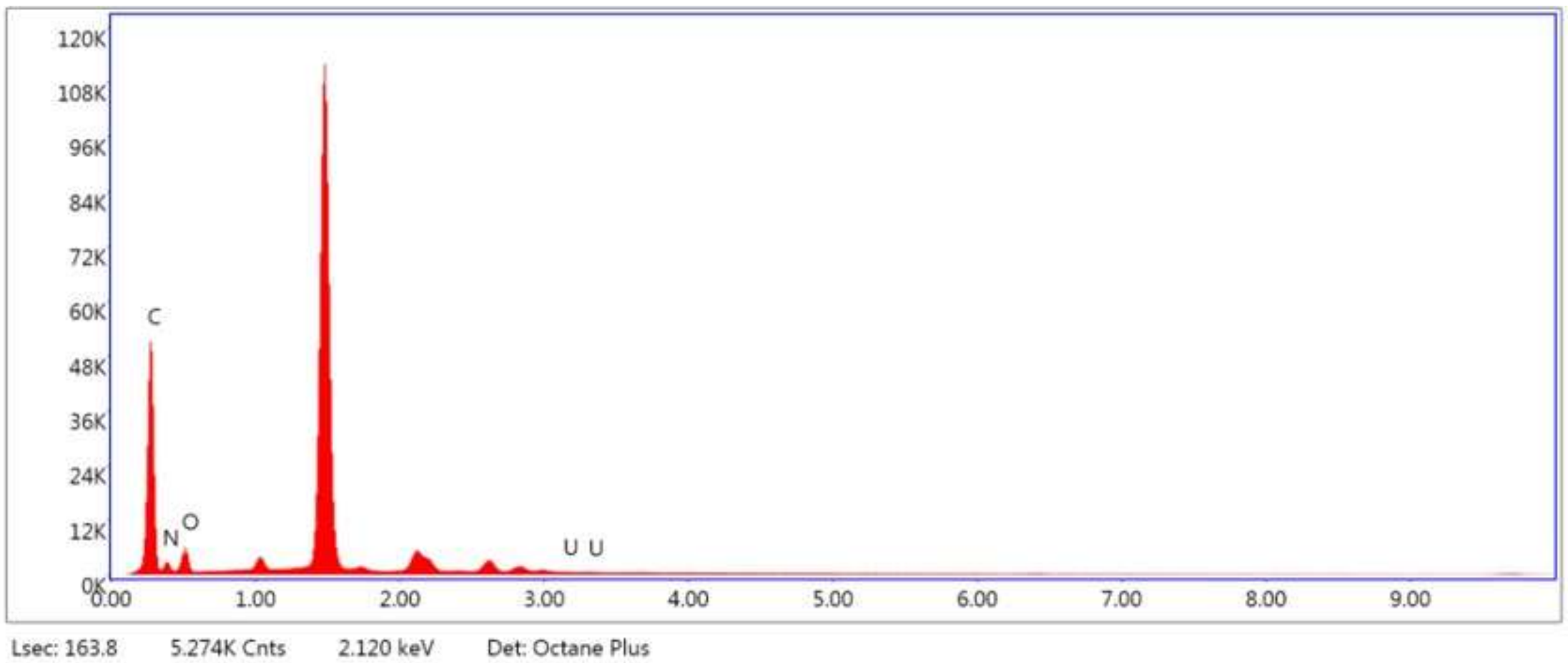

Figure S36. EDAX spectrum of UO_2^{2+}@*Im-POP*. The sample was drop-cast onto an Al substrate for imaging.

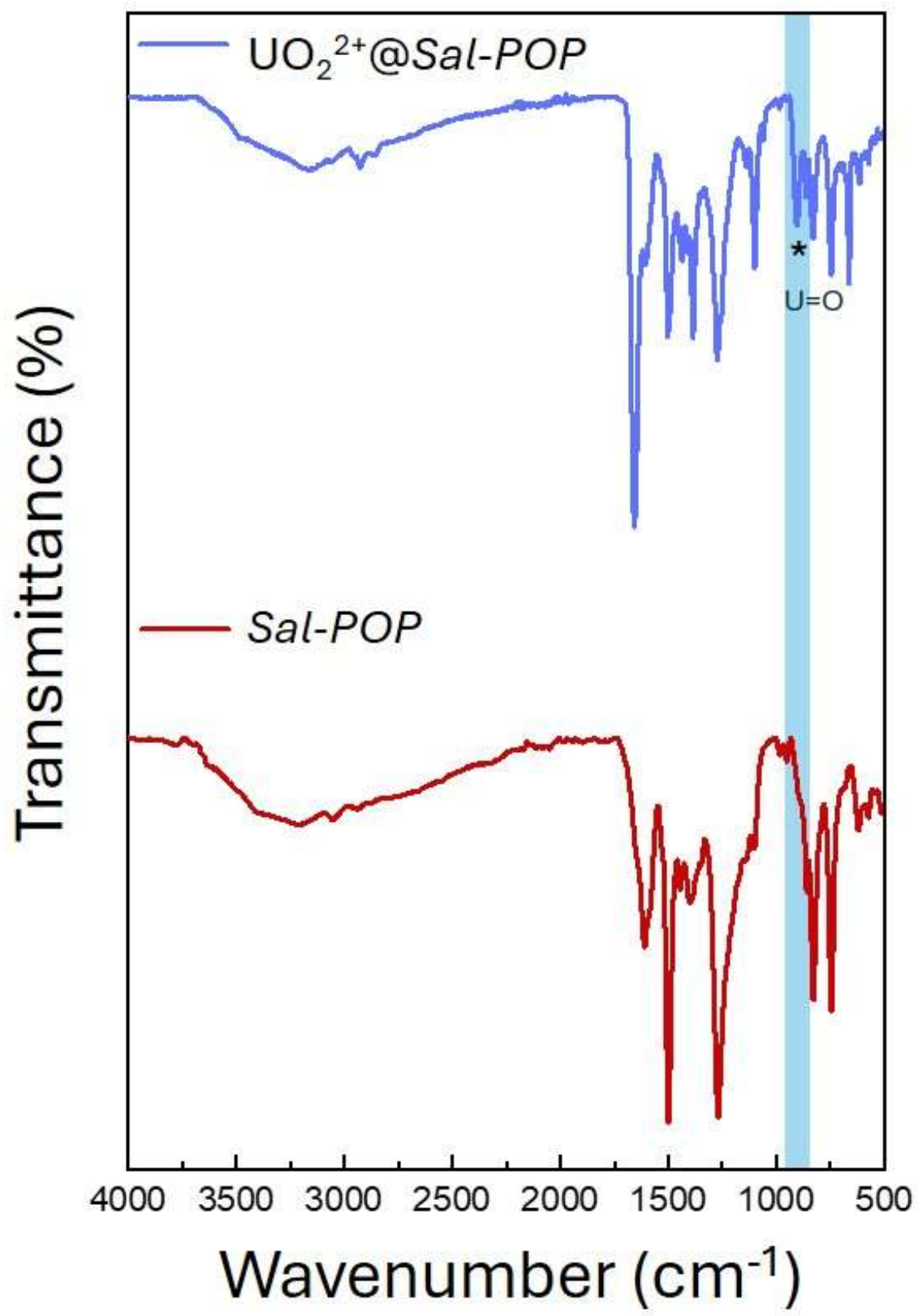

Figure S37. FTIR spectra of UO_2^{2+}@*Sal-POP* (blue), compared with *Sal-POP* (red).

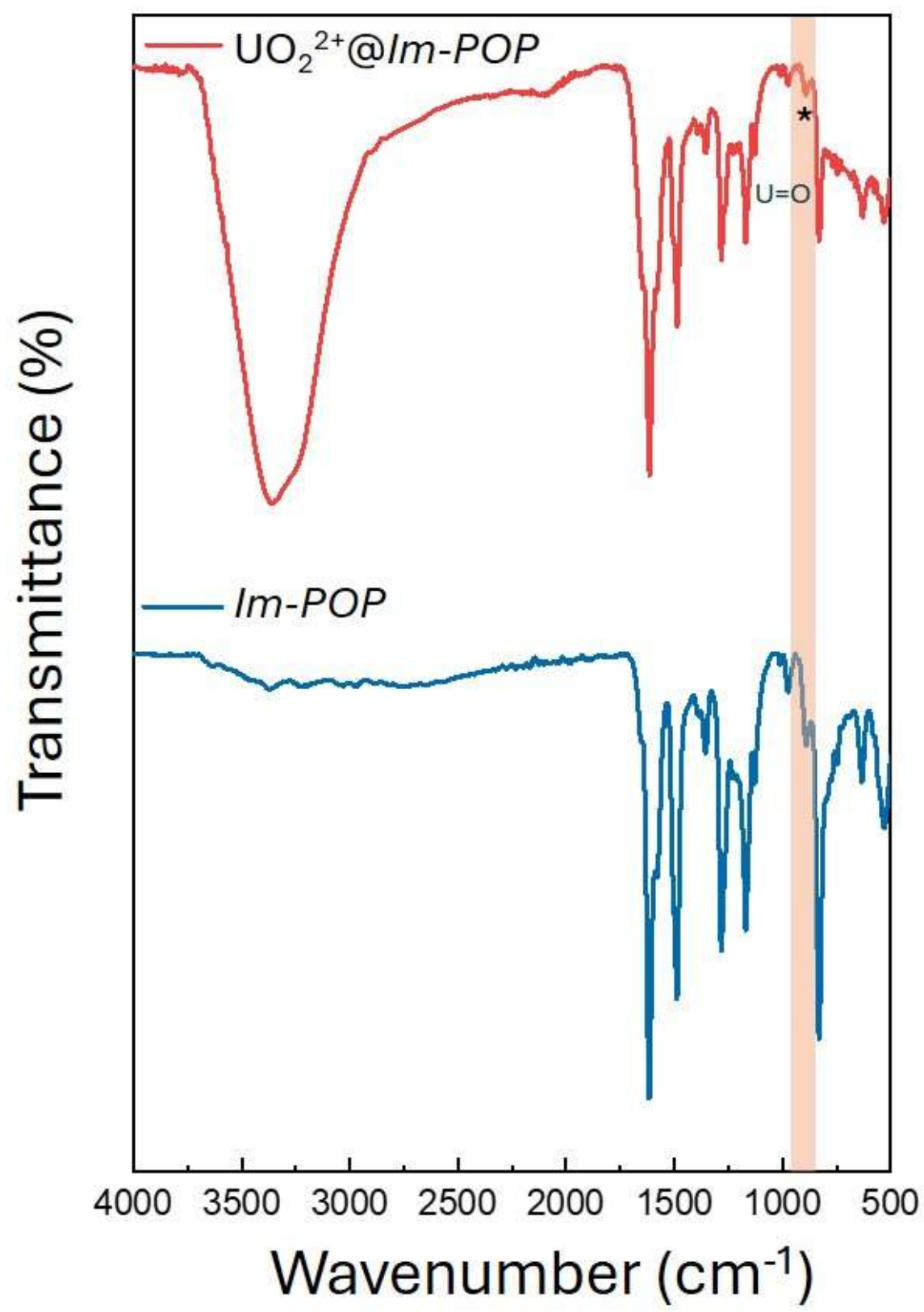

Figure S38. FTIR spectra of UO_2^{2+}@*Im-POP* (red), compared with *Im-POP* (blue).

4. DFT Studies (quantum chemical calculations)

For understanding the binding characteristics of UO_2^{2+} with *Sal-POP* and *Im-POP* density functional theory (DFT) studies were carried out using the plane-wave based DFT code, Vienna Ab-initio Simulation Package (VASP). Since the systems of interest are polymers, we considered the model systems of *Sal-POP* with 120 atoms and *Im-POP* with 114 atoms as reported in **Figure 6 and S39-41** and both the structures were optimized at PBE+D3 level of theory. In the optimized structure of *Sal-POP*, the C-O(OH) distance is found to be 1.34 Å while the N-C(aromatic) and N-C(aliphatic) distances are measured to be 1.40 Å and 1.302 Å, respectively. In the optimized uranyl ion bound *Sal-POP,* i.e., UO_2^{2+}@*Sal-POP* structure reported in **Figure 6a**, U atom is bound to 2 N and 2 O sites of *Sal-POP* along with 2 water molecules. The U-N bond distances are found to be 2.65 Å and 2.77 Å while the U-O(OH) bond distances are found to be 2.60 Å and 2.96 Å. The calculated binding energy of UO_2^{2+}@*Sal-POP* is found to be -2.91 eV. In the case of *Im-POP*, uranyl ion is interacting with one N and one O(OH) sites of the *Im-POP* along with 3 water molecules as shown in **Figure S40**. The measured U-N and U-O distances are found to be 2.59 Å and 2.63 Å, respectively, and the calculated binding energy is found to be -3.12 eV. Charge density difference iso-surfaces are generated for uranyl bound to UO_2^{2+}@*Sal-POP and* UO_2^{2+}@*Im-POP* and are reported in **Figure 6b and S40**. Iso-surfaces in both systems clearly show a charge accumulation around the uranyl ion and charge depletion over *Sal-POP*/*Im-POP,* which is delocalized. We have also generated the band decomposed charge density surfaces for highest occupied, second highest occupied, lowest unoccupied and second lowest unoccupied states of the uranyl bound systems and reported in **Figure 6c-f & S41**. These band decomposed charge density surfaces clearly indicate a strong *f*-π conjugation.[28]

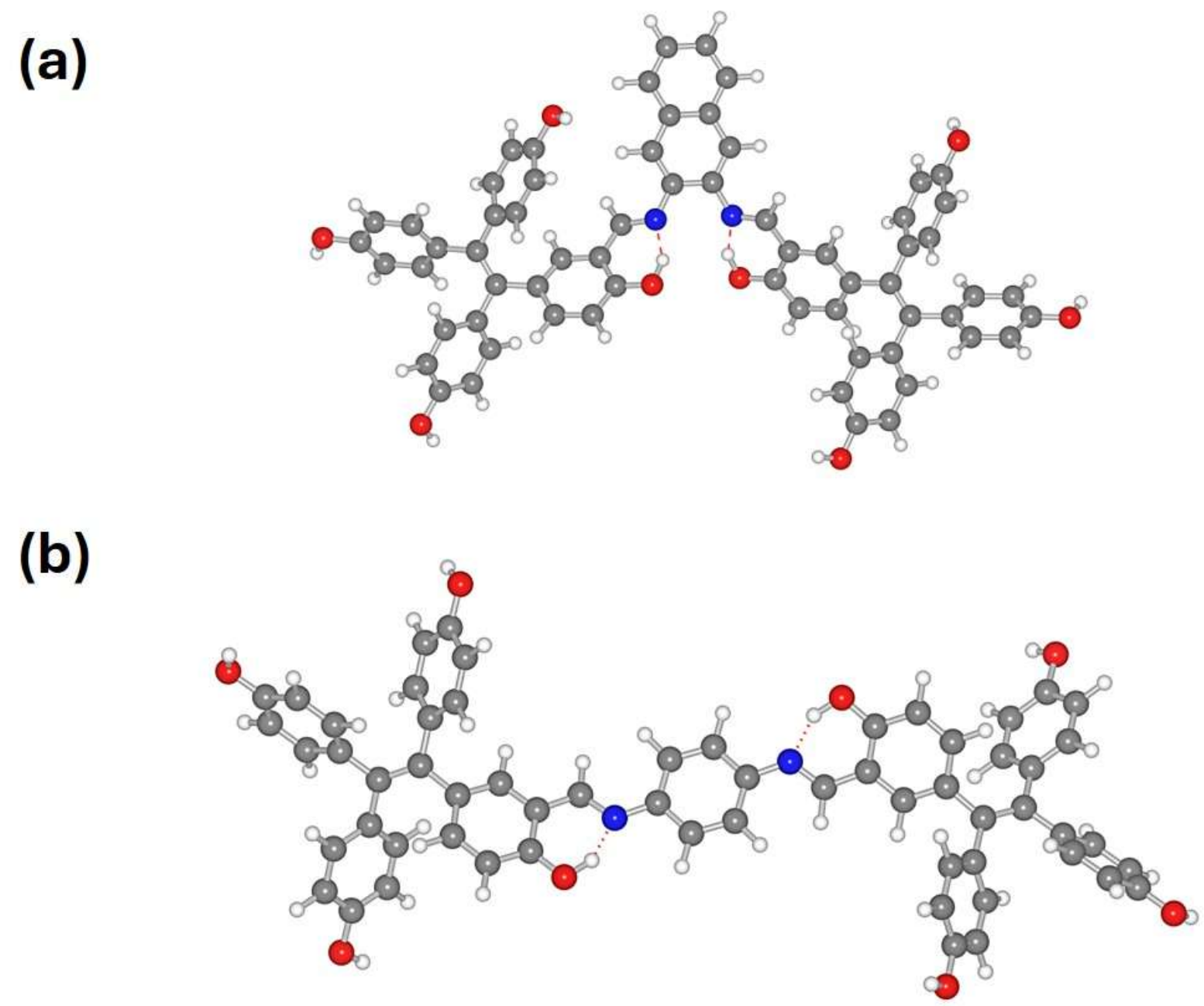

Figure S39. Optimized geometries of (a) *Sal-POP* and (b) *Im-POP* models.

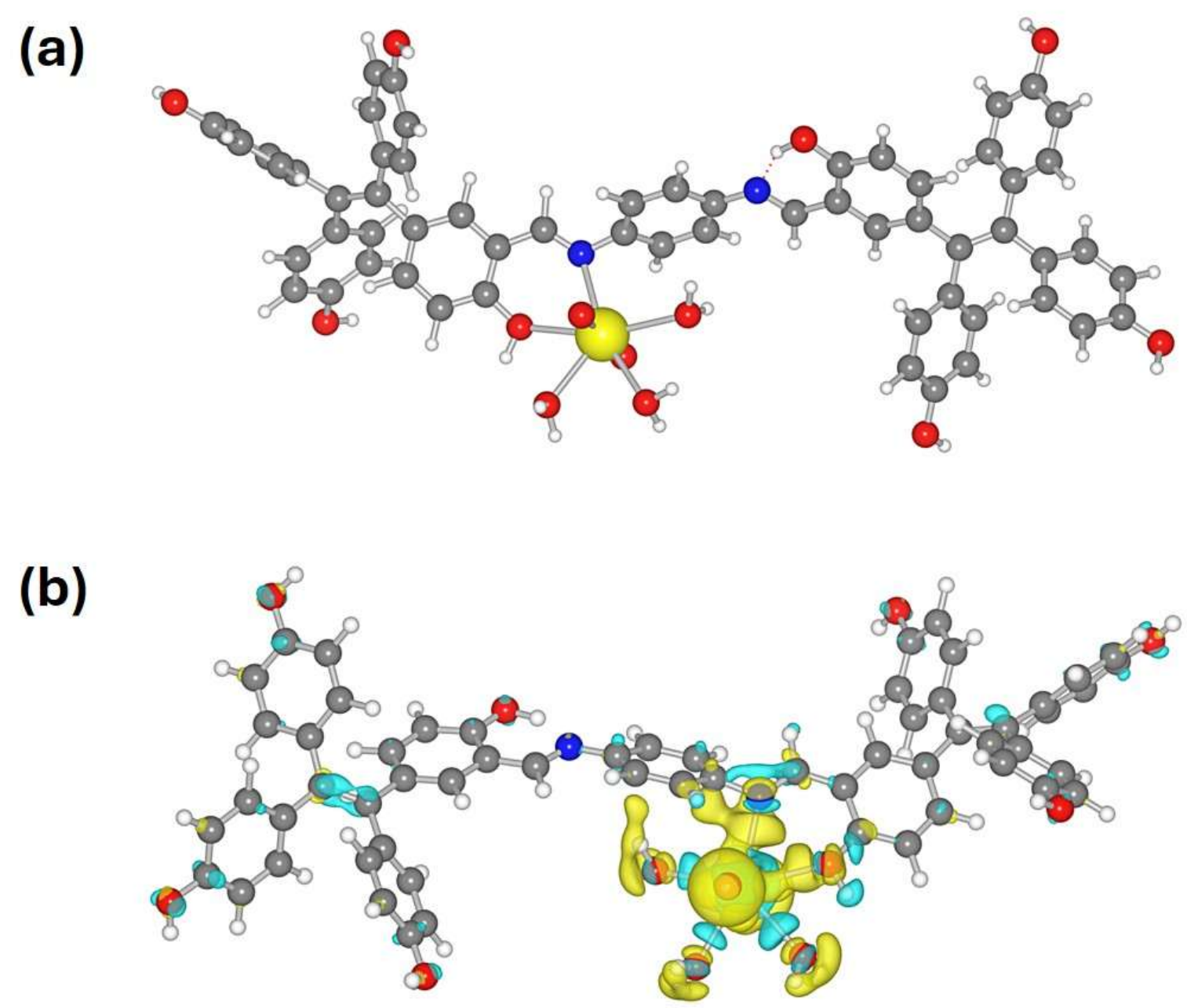

Figure S40. Optimized geometries of (a) UO_2^{2+}@*Im-POP* and (b) Charge density difference iso-surface of UO_2^{2+}@*Im-POP* (Yellow: positive and cyan: negative).

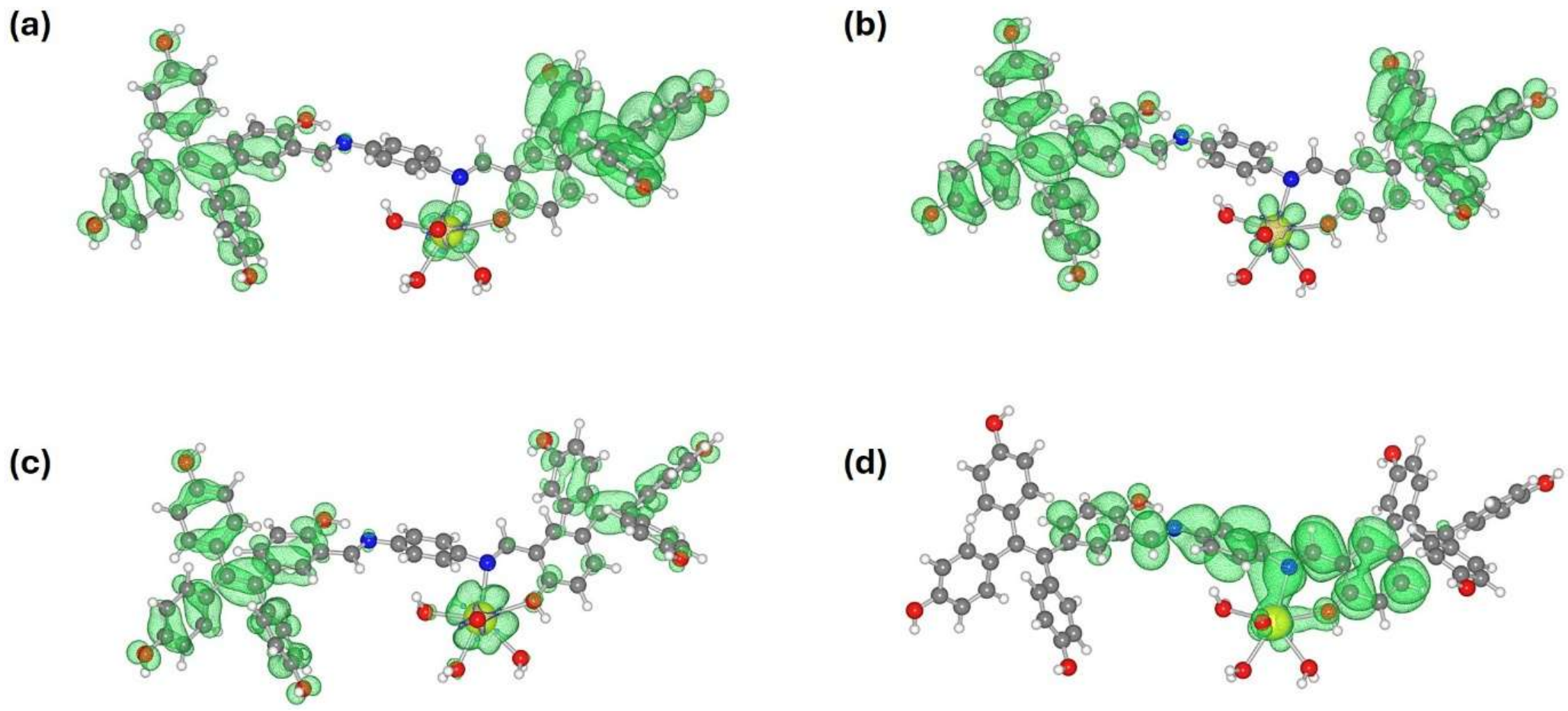

Figure S41. Band decomposed charge density iso-surface of (a) HOMO−1, (b) HOMO, (c) LUMO, and (d) LUMO+1 of UO_2^{+2}@*Im-POP*.

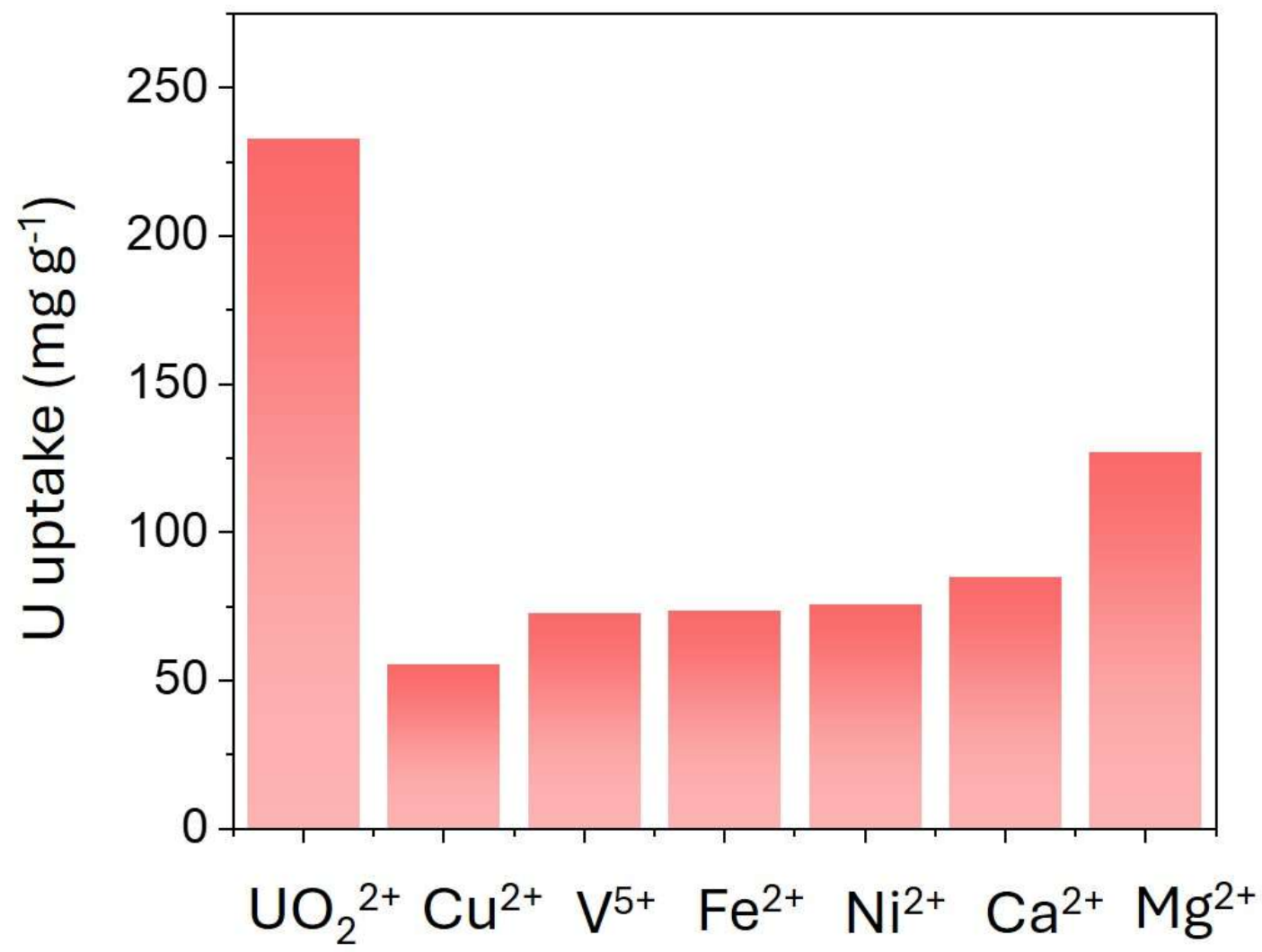

Figure S42. Uranyl uptake capacities of *Im-POP* toward UO_2^{2+} (10 ppm) in the presence of interfering ions under simulated seawater conditions.

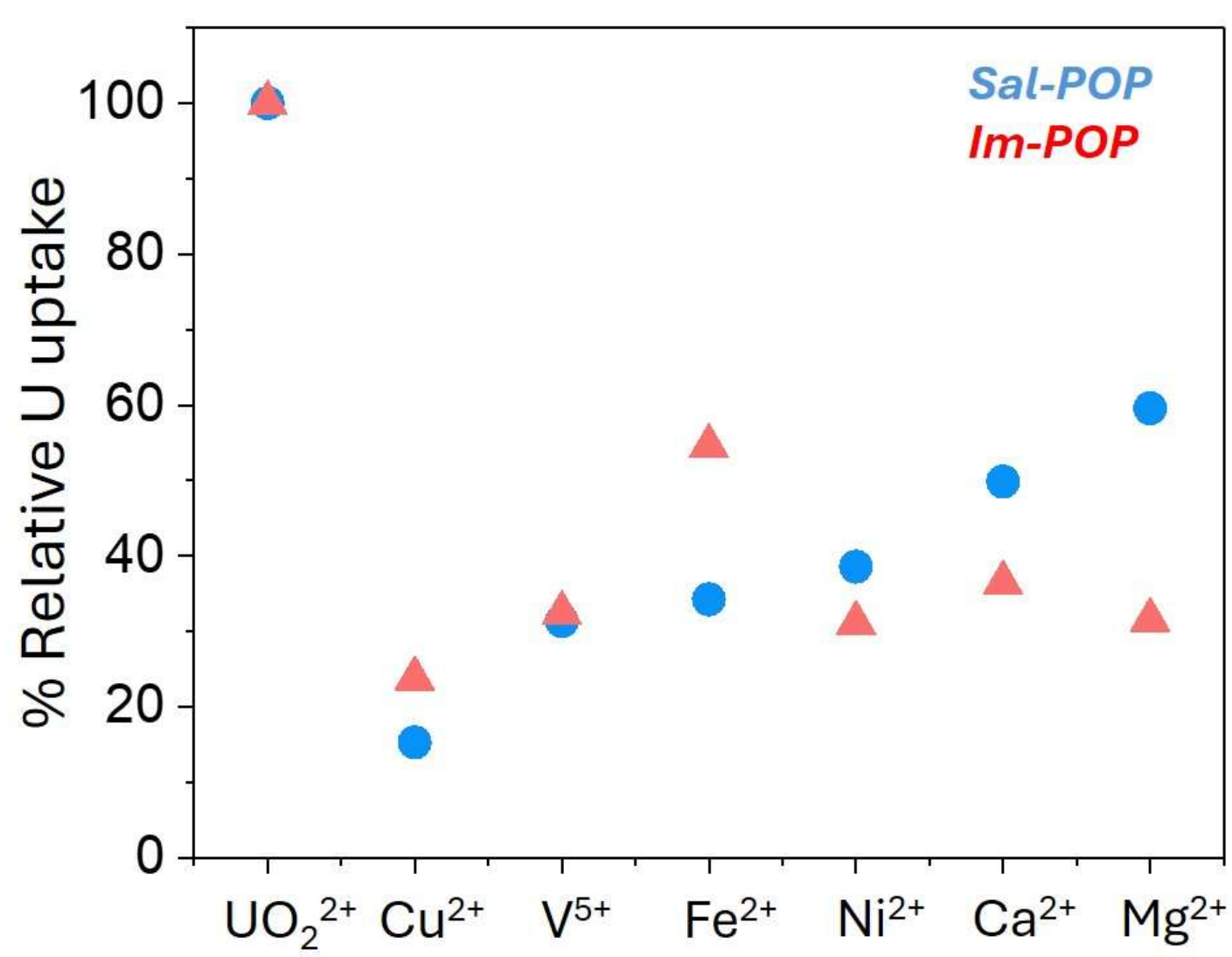

Figure S43. Relative UO_2^{2+} uptake percentage of *Sal-POP* (blue) and *Im-POP* (red) toward UO_2^{2+} in the presence of interfering ions.

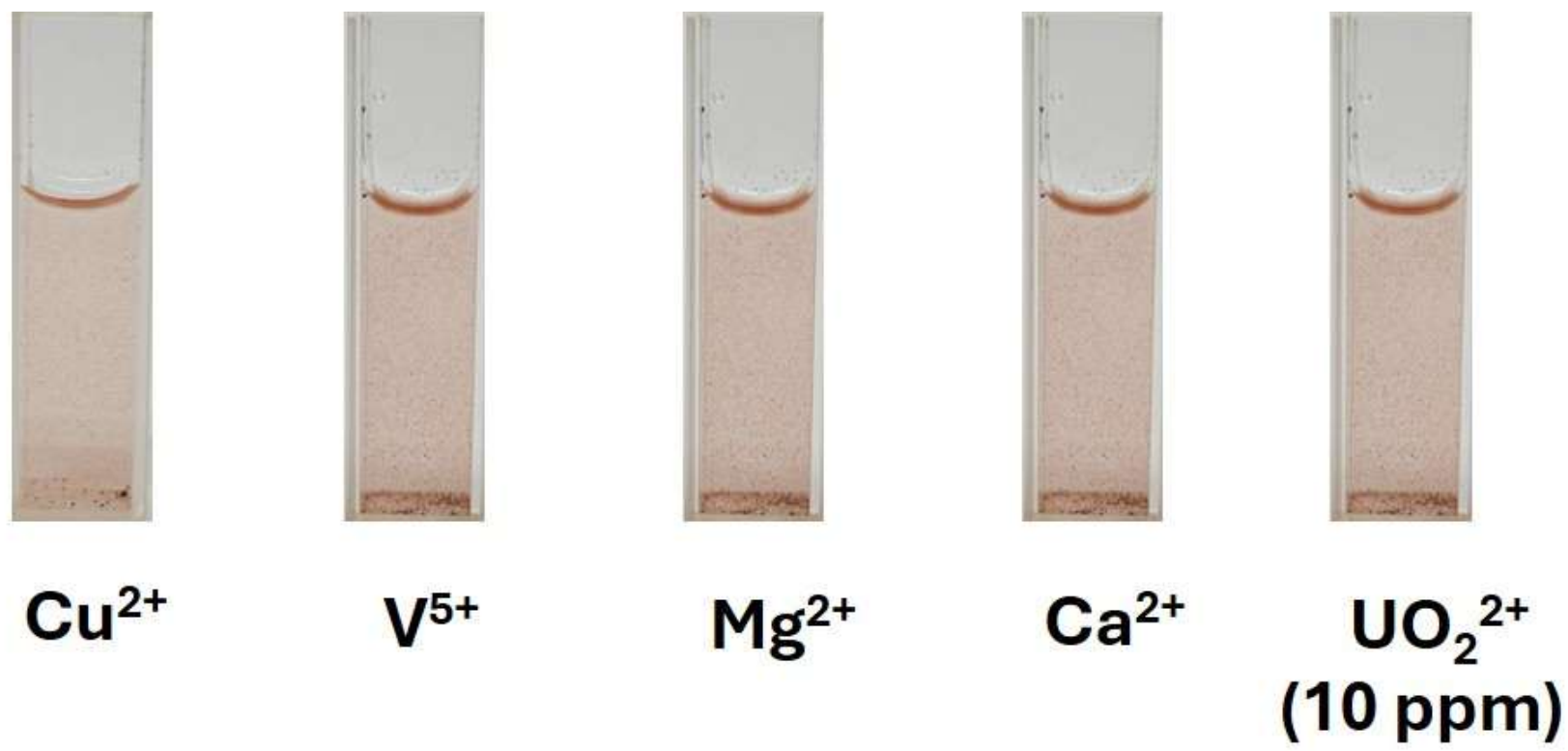

Figure S44. Visible-light images of *Im-POP* dispersions in the presence of different metal ions after 24 h.

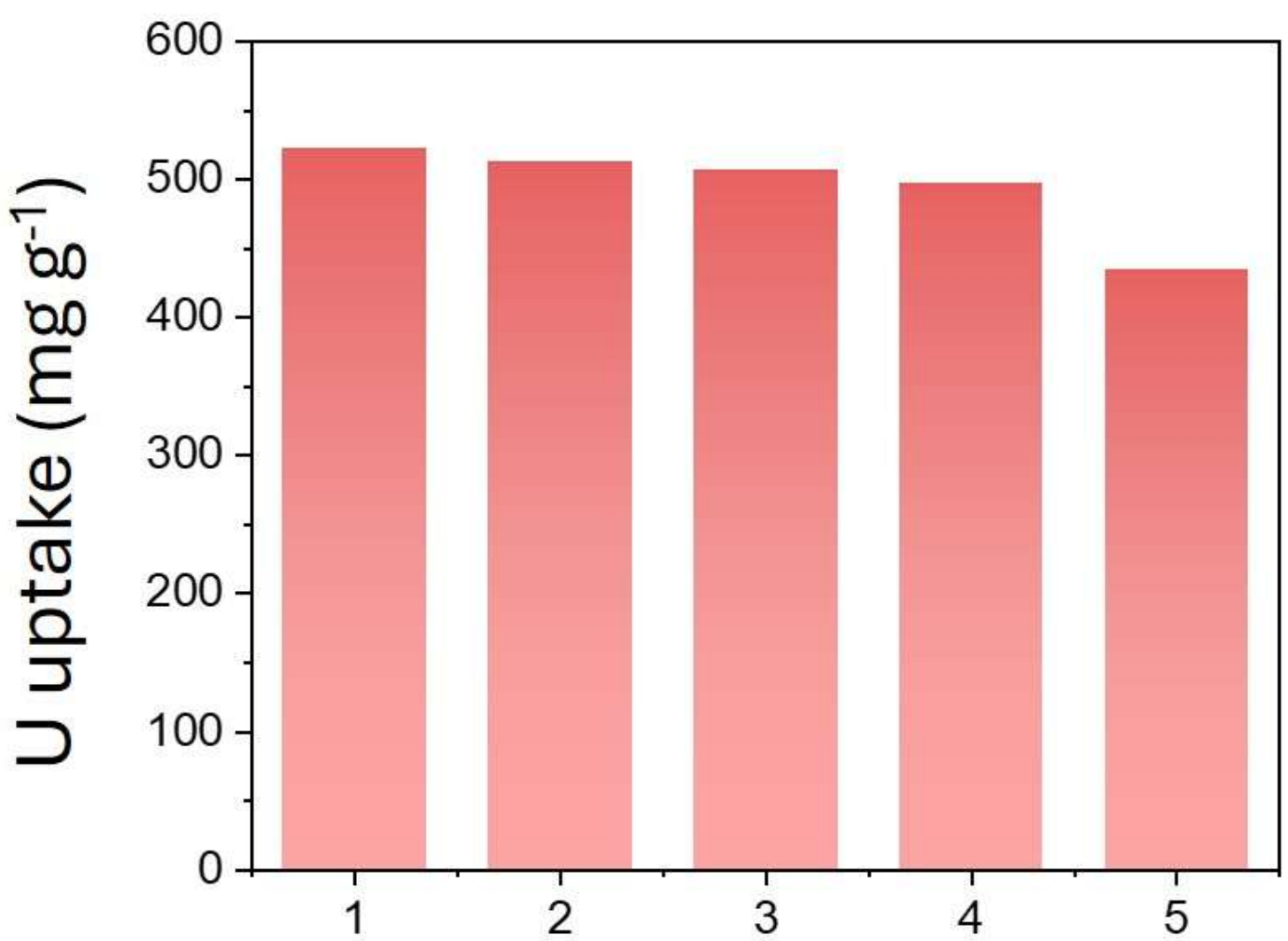

Figure S45. UO_2^{2+} adsorption capacity of *Im-POP* in five successive adsorption–desorption cycles.

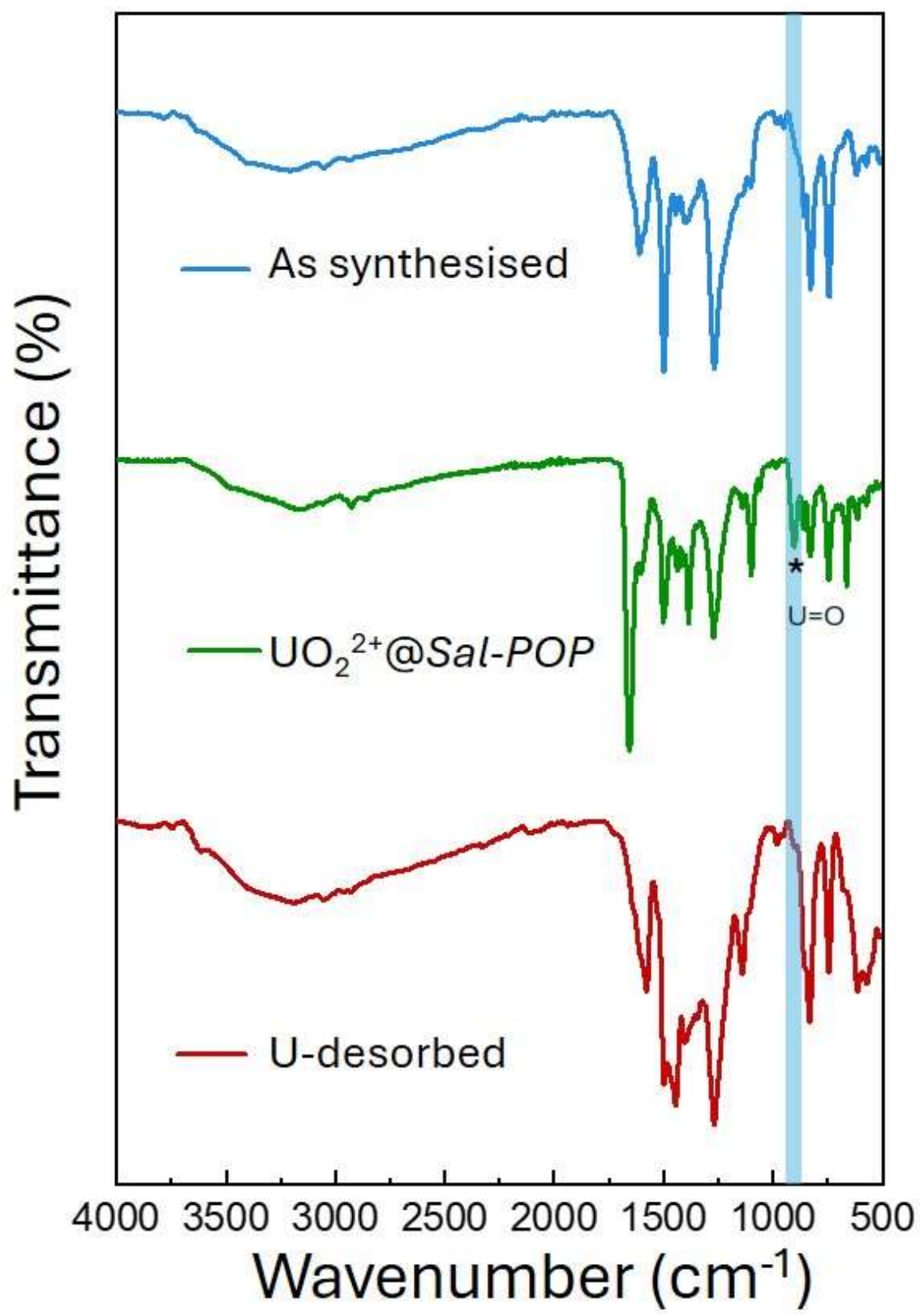

Figure S46. FT-IR spectra of *Sal-POP* (blue), UO_2^{2+}@*Sal-POP* (green), and *Sal-POP* (red) after desorption of UO_2^{2+} by Na_2CO_3 (2 M) aqueous solution.

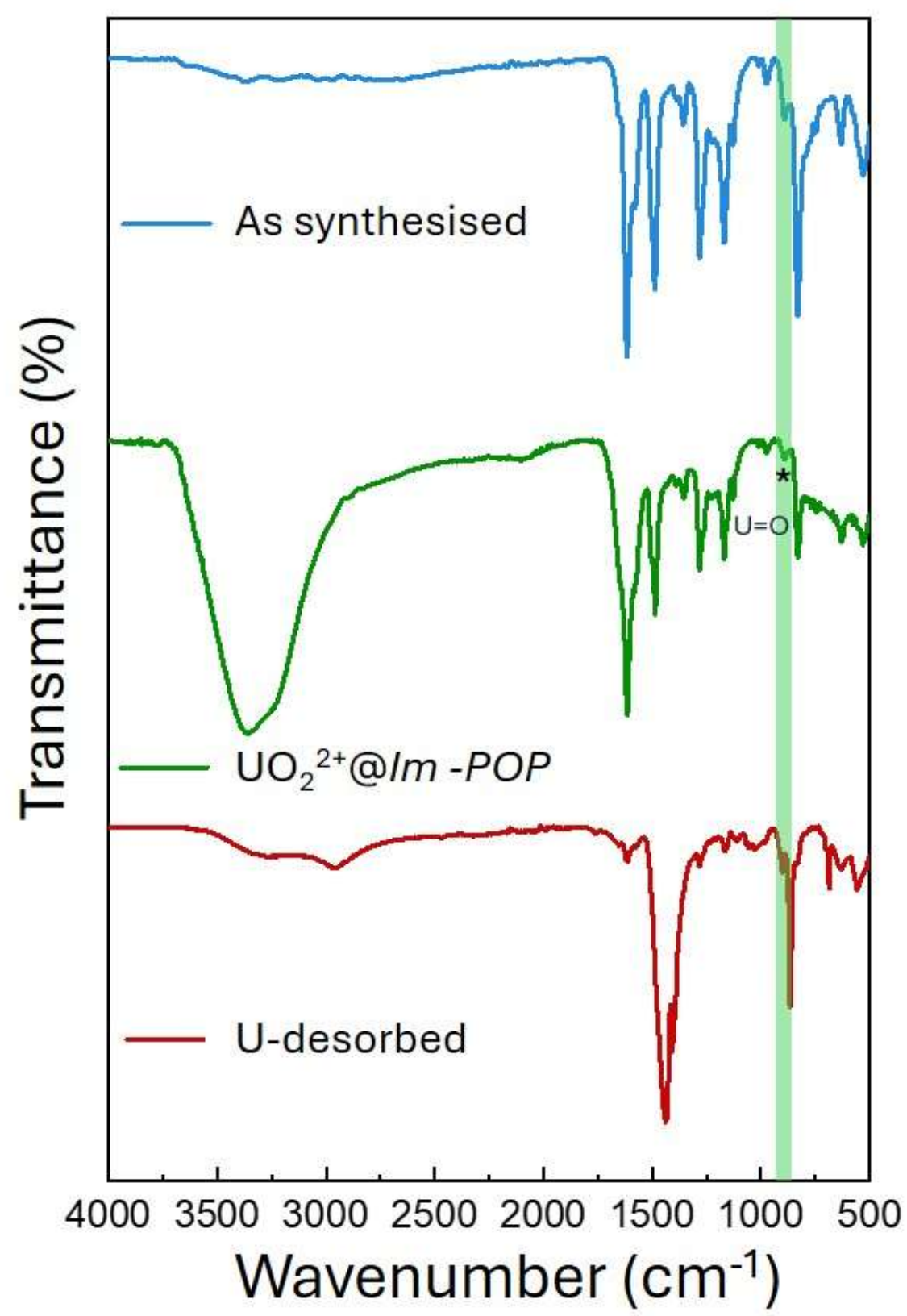

Figure S47. FT-IR spectra of *Im-POP* (blue), UO_2^{2+}@*Im-POP* (green), and *Im-POP* (red) after desorption of UO_2^{2+} by Na_2CO_3 (2 M) aqueous solution.

Table S1: The relative atomic percentage of atoms *of Sal-POP* was identified using XPS survey scan analysis.

**Specific functional group**	**At %**
C	76.36
N	6.07
O	17.57

Table S2: Carbon, nitrogen, and hydrogen content were measured by elemental analysis.

**Sample**	**% Carbon content**	**% Nitrogen content**	**% Hydrogen content**
Sal-POP	75.54	6.95	4.850
Im-POP	78.70	9.26	5.156

Table S3: UO_2^{2+} uptake efficiencies across multiple sorbent systems

Adsorbents	U uptake capacity (mg g^{-1})	Conditions	Reference
TP-COF-AO	436	*m* = 5 mg; *V* = 20 mL; [UO_2^{2+}] ~ 9.25 ppm	15
TFPT-BTAN-AO	427	*m* = 5 mg; *V* = 200 mL; [UO_2^{2+}] ~ 19.9 ppm	16
COF-PDAN-AO	410	*m* = 10 mg; *V* = 20 mL; [UO_2^{2+}] ~ 90.5 ppm	17
GC-TFPB-AO	253.71	*m* = 4 mg; *V* = 20 mL; [UO_2^{2+}] ~ 5 ppm	18
POPAO	251.9	*m: V* ~ 0.25 mg/mL; [UO_2^{2+}] ~ 5×10^{-5} mol/L	7
COF-AO	229.9	*m* = 8 mg; *V* = 200 mL; [UO_2^{2+}] ~ 100 ppm	19
YTU-100	83.43	*m* = 60 mg; *V* = 60 mL; [UO_2^{2+}] ~ 5 ppm	20
TPPa-2OH-Phos	410.9	*m: V* ~ 1/10 g/L; [UO_2^{2+}] ~ 100 ppm	21
UiO-66-3C4N	380.3	*m* = 5 mg; *V* = 500 mL; [UO_2^{2+}] ~ 8 ppm	22
COF-SO_3H	360	*m* = 10 mg; *V* = 20 mL; [UO_2^{2+}] ~ 50-600 ppm	23
POP-TpDb-AO	355	*m* = 4.5 mg; *V* = 400 mL; [UO_2^{2+}] ~ 9.25 ppm	24
FJSM-SnS	338	*V:m* ~ 1000 mL/g; [UO_2^{2+}] ~ 10-603 ppm	25
Anti-UiO-66	295.95	*m* = 10 mg; *V* = 1000 mL; [UO_2^{2+}] ~ 8 ppm	26
MITpBD	206.6	*m* = 5 mg; *V* = 500 mL; [UO_2^{2+}] ~ 10 ppm	27
Sal-POP	450	*m* = 5mg; *V* = 400 mL; [UO_2^{2+}] ~ 30 ppm	This Work
Im-POP	523	*m* = 5 mg; *V* = 400 mL; [UO_2^{2+}] ~ 30 ppm	This Work

Table S4: LOD values across multiple sorbent systems

Adsorbents	Fluorescence/ LOD (nM)	Reference
TP-COF-AO	8.3	15
TFPT-BTAN-AO	6.7	16
COF-PDAN-AO	6.5	17
GC-TFPB-AO	21.25	18
POPAO	1.7	7
COF-AO	14.6	19
YTU-100	4.0	20
Sal-POP	7.65	This Work

References

[1] N. Sahoo, A. Kapoor, M. Yadav, S. K. Rajput, V. S. Mothika, "Reversible Dual-Mode Detection of Cu^{2+} and Tandem Capture of Cysteine in a Salphen-Conjugated Microporous Polymer," *Sensors & Diagnostics* 4 (2025): 846–855.

[2] H. Qu, X. Tang, X. Wang, Z. Li, Z. Huang, H. Zhang, Z. Tian, X. Cao, "Chiral molecular face-rotating sandwich structures constructed through restricting the phenyl flipping of tetraphenylethylene," *Chem. Sci.* 9 (2018): 8814–8818.

[3] J.-B. Xiong, H.-T. Feng, J.-P. Sun, W.-Z. Xie, D. Yang, M. Liu, Y.-S. Zheng, "The Fixed Propeller-Like Conformation of Tetraphenylethylene that Reveals Aggregation-Induced Emission Effect, Chiral Recognition, and Enhanced Chiroptical Property," *J. Am. Chem. Soc.* 138 (2016): 11469–11472.

[4] M. Donnier-Maréchal, S. Abdullayev, M. Bauduin, Y. Pascal, M.-Q. Fu, X.-P. He, E. Gillon, A. Imberty, E. Kipnis, R. Dessein, S. Vidal, "Tetraphenylethylene-based glycoclusters with aggregation-induced emission (AIE) properties as high-affinity

ligands of bacterial lectins," *Org. Biomol. Chem.* 16 (2018): 8804–8809.

[5] K. Patra, S. Mollick, H. Pal, A. Sengupta, R. Saha, S. Dey, J. Selvakumar, "Highlighting the Advancement of Nuclear Waste Water Treatment with Modular Porous Scaffolds," *ACS Appl. Mater. Interfaces* 17 (2025): 61571.

[6] S. A. Darda, H. A. Gabbar, V. Damideh, M. Aboughaly, I. Hassen, "A comprehensive review on radioactive waste cycle from generation to disposal," *J. Radioanal. Nucl. Chem.* 329 (2021): 15.

[7] M. Xu, T. Wang, P. Gao, L. Zhao, L. Zhou, D. Hua, "Highly fluorescent conjugated microporous polymers for concurrent adsorption and detection of uranium," *J. Mater. Chem. A* 7 (2019): 11214.

[8] G. Kresse, J. Furthmüller, "Efficiency of ab-initio total energy calculations for metals and semiconductors using a plane-wave basis set," *Comput. Mater. Sci.* 6 (1996): 15–50.

[9] G. Kresse, J. Furthmüller, "Efficient iterative schemes for ab initio total-energy calculations using a plane-wave basis set," *Phys. Rev. B.* 54 (1996): 11169–11186.

[10] G. Kresse, D. Joubert, "From ultrasoft pseudopotentials to the projector augmented-wave method," *Phys. Rev. B.* 59 (1999): 1758–1775.

[11] P. E. Blöchl, "Projector augmented-wave method," *Phys. Rev. B.* 50 (1994): 17953–17979.

[12] J. P. Perdew, K. Burke, M. Ernzerhof, "Generalized Gradient Approximation Made Simple," *Phys. Rev. Lett.* 77 (1996): 3865–3868.

[13] S. Grimme, S. Ehrlich, L. Goerigk, "Effect of the damping function in dispersion corrected density functional theory," *J. Comput. Chem.* 32 (2011): 1456–1465.

[14] K. Momma, F. Izumi, "VESTA 3 for three-dimensional visualization of crystal, volumetric and morphology data," *J. Appl. Crystallogr.* 44 (2011): 1272–1276.

[15] C.-R. Zhang, W.-R. Cui, W. Jiang, F.-F. Li, Y.-D. Wu, R.-P. Liang, J.-D. Qiu, "Simultaneous sensitive detection and rapid adsorption of UO_2^{2+} based on a post-modified sp^2 carbon-conjugated covalent organic framework," *Environ. Sci. Nano*. 7 (2020): 7842.

[16] W. R. Cui, C. R. Zhang, W. Jiang, F. F. Li, R. P. Liang, J. Liu, J. D. Qiu, "Regenerable and stable sp^2 carbon-conjugated covalent organic frameworks for selective detection and extraction of uranium," *Nat. Commun.* 11 (2020): 436.

[17] F.-F. Li, W.-R. Cui, W. Jiang, C.-R. Zhang, R.-P. Liang, J.-D. Qiu, "Stable sp^2 carbon-conjugated covalent organic framework for detection and efficient adsorption of uranium from radioactive wastewater," *J. Hazard. Mater.* 392 (2020): 122333.

[18] D. Zhen, C. Liu, Q. Deng, L. Li, C. A. Grimes, S. Yang, Q. Cai, Y. Liu, "Novel Olefin-Linked Covalent Organic Framework with Multifunctional Group Modification for the Fluorescence/Smartphone Detection of Uranyl Ion," *ACS Appl. Mater. Interfaces* 16 (2024): 27804.

[19] Y. Luo, N. Li, J. Tian, J. Pan, R. Su, V. Turkevich, L. Wang, "Two birds with one stone strategy: selective uranium extraction and detection by a fluorescent covalent organic framework with high-affinity binding sites," *J. Mater. Chem. A.* 13 (2025): 37458.

[20] M. Lei, Y. Jia, W. Zhang, J. Xie, Z. Xu, Y. Wang, W. Du, W. Liu, "Ultrasensitive and Selective Detection of Uranium by a Luminescent Terbium–Organic Framework," *ACS Appl. Mater. Interfaces* 13 (2021): 51086.

[21] D. Mei, B. Yan, "β-Ketoenamine and Benzoxazole-Linked Covalent Organic Frameworks Tailored with Phosphorylation for Efficient Uranium Removal from Contaminated Water Systems," *Adv. Funct. Mater.* 34 (2024): 2313314.

[22] Y. Yuan, S. Feng, L. Feng, Q. Yu, T. Liu, N. Wang, "A Bio-inspired Nano-pocket Spatial Structure for Targeting Uranyl Capture," *Angew. Chemie - Int. Ed.* 59 (2020): 4262.

[23] X. H. Xiong, Z. W. Yu, L. Le Gong, Y. Tao, Z. Gao, L. Wang, W. H. Yin, L. X. Yang, F. Luo, "Ammoniating Covalent Organic Framework (COF) for High-Performance and Selective Extraction of Toxic and Radioactive Uranium Ions," *Adv. Sci.* 6 (2019): 1900547.

[24] Q. Sun, B. Aguila, L. D. Earl, C. W. Abney, L. Wojtas, P. K. Thallapally, S. Ma, "Covalent Organic Frameworks as a Decorating Platform for Utilization and Affinity Enhancement of Chelating Sites for Radionuclide Sequestration," *Adv. Mater.* 30 (2018): 1705479.

[25] M.-L. Feng, D. Sarma, X.-H. Qi, K.-Z. Du, X.-Y. Huang, M. G. Kanatzidis, "Efficient Removal and Recovery of Uranium by a Layered Organic–Inorganic Hybrid Thiostannate," *J. Am. Chem. Soc.* 138 (2016): 12578.

[26] Q. Yu, Y. Yuan, J. Wen, X. Zhao, S. Zhao, D. Wang, C. Li, X. Wang, N. Wang, "A Universally Applicable Strategy for Construction of Anti-Biofouling Adsorbents for Enhanced Uranium Recovery from Seawater," *Adv. Sci.* 6 (2019): 1900002.

[27] Y. Song, Y. Zheng, J. Cao, S. Chen, Y. Song, F. Wang, "High-capacity uranium extraction from seawater through constructing synergistic multiple dynamic bonds," *Nat. Water 3* (2025): 89.

[28] Q. Y. Zhang, L. J. Zhang, J. Q. Zhu, L. Le Gong, Z. C. Huang, F. Gao, J. Q. Wang, X. Q. Xie, F. Luo, "Ultra-selective uranium separation by in-situ formation of *π*-

f conjugated 2D uranium-organic framework," *Nat. Commun.* 15 (2024): 453.
